# Risk factors of nonalcoholic fatty liver disease in lean body mass population: A systematic review and meta‐analysis

**DOI:** 10.1002/jgh3.12658

**Published:** 2021-10-04

**Authors:** Shahinul Alam, Mohammad Eslam, Nazmul SKM Hasan, Kamrul Anam, Mohammad Abdul Baker Chowdhury, Md Abdullah Saeed Khan, Mohammad J Hasan, Rosmawati Mohamed

**Affiliations:** ^1^ Department of Hepatology Bangabandhu Sheikh Mujib Medical University Dhaka Bangladesh; ^2^ Storr Liver Centre, Westmead Institute for Medical Research Westmead Hospital and University of Sydney Sydney New South Wales Australia; ^3^ Department of Hepatology Shaheed Syed Nazrul Islam Medical College Kishoreganj Bangladesh; ^4^ Department of Medical Gastroenterology Sheikh Russel National Gastroliver Institute and Hospital Dhaka Bangladesh; ^5^ Department of Emergency Medicine University of Florida College of Medicine Gainesville Florida USA; ^6^ Meta analysis Division Pi Research Consultancy Center Dhaka Bangladesh; ^7^ Department of Pharmacology Shaheed Sayed Nazrul Islam Medical College Kishoreganj Bangladesh; ^8^ Department of Medicine University Malaya Medical Centre Kuala Lumpur Malaysia

**Keywords:** lean, meta‐analysis, nonalcoholic fatty liver disease, nonalcoholic steatohepatitis, nonlean, nonobese, risk factors, systematic review

## Abstract

The pathophysiology and risk factors of nonalcoholic fatty liver disease (NAFLD) among lean patients is poorly understood and therefore investigated. We performed a meta‐analysis of observational studies. Of 1175 articles found through searching from Medline/PubMed, Banglajol, and Google Scholar by two independent investigators, 22 were selected. Data from lean (*n* = 6768) and obese (*n* = 9253) patients with NAFLD were analyzed; lean (*n* = 43 398) and obese (*n* = 9619) subjects without NAFLD served as controls. Age, body mass index, waist circumference, systolic blood pressure, and diastolic blood pressure (DBP) had significantly higher estimates in lean NAFLD patients than in lean non‐NAFLD controls. Fasting blood sugar [MD(mean difference) 5.17 mg/dl, 95% CI(confidence interval) 4.14–6.16], HbA1c [MD 0.29%, 95% CI 0.11–0.48], and insulin resistance [HOMA‐IR] [MD 0.49 U, 95% CI 0.29–0.68]) were higher in lean NAFLD patients than in lean non‐NAFLD controls. All components of the lipid profile were raised significantly in the former group except high‐density lipoprotein. An increased uric acid (UA) level was found to be associated with the presence of NAFLD among lean. Cardio‐metabolic profiles of nonlean NAFLD patients significantly differs from the counter group. However, the magnitude of the difference of lipid and glycemic profile barely reached statistical significance when subjects were grouped according to lean and nonlean NAFLD. But DBP (slope: 0.19, *P* < 0.037), HOMA‐IR (slope: 0.58, *P* < 0.001), and UA (slope: 0.36, *P* = 0.022) were significantly higher if NAFLD was present compared to that of non‐NAFLD group. Lean and nonlean NAFLD patients are metabolically similar and share common risk factors.

## Graphical abstract

A meta‐analysis of 22 articles comprising data from lean (*n* = 6768) and obese (*n* = 9253) patients with nonalcoholic fatty liver disease (NAFLD), and lean (*n* = 43398) and obese (*n* = 9619) subjects without NAFLD (healthy) revealed that both lean and obese patients shared similar risk factors associated with NAFLD including age, body mass index, waist circumference, systolic blood pressure, diastolic blood pressure, HbA1c, insulin resistance (HOMA‐IR), total cholesterol, low‐density lipoprotein, high‐density lipoprotein, triglycerides, and uric acid.

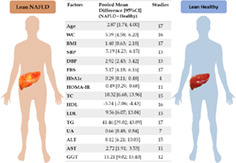

## Introduction

Nonalcoholic fatty liver disease (NAFLD) is an emerging public health problem.^1^, ^2^ It encompasses a spectrum of diseases from nonalcoholic fatty liver to nonalcoholic steatohepatitis (NASH) and fibrosis.^3^ It is the most common cause of chronic liver disease^4^ and has been identified as the leading etiology of liver transplantation worldwide.^5^

The pathogenesis of the NAFLD is multifactorial and the underlying mechanism yet to be fully understood. Most mechanisms for developing NAFLD are linked with changes in lipid metabolism and development of insulin resistance.^6^, ^7^, ^8^ Former thought about underlying pathophysiology was based on excess body fat or obesity but recent trends of NAFLD in lean patients with a body mass index (BMI) <25 kg/m^2^ has changed that thought.^9^, ^10^, ^11^ The role of factors like diet, ethnicity, derangement of gut liver axis, and gut microbiota came into light.^12^, ^13^, ^14^, ^15^ While this phenomenon was initially observed in Asian population,^16^ it has now been recognized as a global health issue.^17^ In addition, due to increased incidence of NAFLD in lean (nonobese) patients, research focus has recently been emphasized to this population.^16^ However, obesity, age, advanced insulin resistance, or type 2 diabetes mellitus have repeatedly been reported as risk factors for progression from NAFLD to NASH.^6^, ^11^, ^12^, ^14^, ^18^

Overall prevalence of lean NAFLD among general population was 10.2%, and among the NAFLD population the prevalence of lean NAFLD was 19.2% with a broad countrywide variation.^19^, ^20^ In an average around 40% of people with NAFLD are not obese but they have severe histological phenotype like that of obese people. Mortality rate is higher and almost 40% of nonobese people with NAFLD have NASH and almost 30% have significant fibrosis.^13^, ^18^, ^19^, ^20^ In addition, the management plan of both group of patients has a distinct difference. For example: successful weight reduction strategy has potential benefits in nonlean patients but there are limited benefits in lean NAFLD patients.^21^ Based on the previous literature and considering the differences in the underlying mechanisms of lean and nonlean NAFLD, we hypothesized that risks factors of NAFLD might differ between those groups and needs to be assessed. Therefore, the aim of this study was to systematically evaluate the risk factors of lean NAFLD and to aid in management strategies for the prevention and control of NAFLD and related diseases.

## Methods

This work was delineated on the basis of the Preferred Reporting Items for Systematic Reviews and Meta‐Analysis Protocols (PRISMA‐P) guidelines^22^ for evaluating the risk factor responsible for development of NAFLD in lean individuals. A PRISMA‐P checklist for this work is attached (Table S1, Supporting information). The protocol was registered in the International Prospective Register of Systematic Reviews (PROSPERO registration number: CRD42019136129).

Ethical clearance was waivered as there was no involvement of live subjects. However, formal notification and permission was taken from Institutional Review Board (IRB) of Bangabandhu Sheikh Mujib Medical University (BSMMU), Dhaka, Bangladesh. IRB registration no: BSMMU/2020/1012.

### Search strategy and selection criteria

This meta‐analysis was based on systematic searches in several electronic databases comprising Medline/PubMed, BanglaJOL, and Google Scholar from inception to 30 March 2020. The keywords used during searching were made before final searching in collaboration with an experienced medical librarian so that as many relevant articles as could be retrieved. To enhance the sensitivity, bibliographic search of the selected article was also performed for additional article. Our search term for PubMed (from inception to 30 March 2020) with details of our search strategy and data collection, including terms for other databases, are described in the Table S2.

We included original research articles that defined their population as nonobese or lean individuals aged 18‐years or older and defined NAFLD with stratification according to weight status (nonobese or lean). In this review, lean was defined as patients with a BMI <25 kg/m^2^ and nonlean was defined when BMI ≥25 kg/m^2^. Hence only studies using this cut‐off point for differentiating nonlean (obese) from lean (nonobese) were included. Studies that described possible risk factors of NAFLD or lean NAFLD/NASH patients were considered for initial inclusion and there was no restriction in accordance with study setting either hospital or community. We included prospective comparative cohort studies of which baseline data is available, case–control studies, and cross‐sectional studies which detailed the risk factors of lean NAFLD.

The exclusion criteria for studies were: reporting population <18 years of age including children, or people with mental disorders or studies concerning patients with other liver diseases or causes for steatosis, and alcoholic fatty liver disease or articles including patients who have a daily alcohol consumption ≥30 g for men and ≥20 g for women.^23^ Studies unable to ascertain how NAFLD was diagnosed were also removed. Randomized controlled trials (RCTs), systematic reviews, review articles, trial protocols, ongoing trials, editorials, letters, and conference papers were excluded from the analysis. Articles which do not have full texts available or lack of information regarding age, sex, country of origin, study design, method of assessment of fatty liver infiltration, anthropometric variables (waist circumference [WC]) or exclusive to one gender and duplicate publication were excluded. Gray literatures were also excluded. Inclusion of the studies were restricted to those published in English.

In order to exclude articles irrelevant to the systematic review, two investigators (Mohammad Jahid Hasan and Md Abdullah Saeed Khan) initially independently reviewed the title and abstract of each the references using rigorous inclusion criteria. Any dispute between two investigators were resolved through discussion with the principal investigator (Shahinul Alam). Then search results passed on to Mendeley (reference management software) which excluded the duplicates. In the second stage, the two investigators independently read the full texts of the articles that were included in the initial stage, and then selected the articles that met the inclusion criteria. Differences of ideas regarding the selection of articles were resolved through group discussion. In cases where details were missing on study design, population, intervention, or outcomes, the authors of included studies were contacted by email. After the first contact attempt, if no response received, the study authors were contacted two more times approximately 3 to 4 weeks apart. The searches were re‐run just before the final analyses and further studies retrieved for inclusion were checked. The literature search, data review, and data extraction were done with a case report form to provide consistency throughout the data collection process. Data were extracted independently by two investigators (any of two Mohammad Jahid Hasan, Md Abdullah Saeed Khan, Kamrul Anam, and S K M Nazmul Hasan). Discordance and disagreements were resolved by consensus between the two investigators or by consultation with the principal investigator (Shahinul Alam).

### Assessment of study quality

We used a quality assessment scale based on the Newcastle‐Ottawa Scale (NOS) for this study, ranging from 0 to 9, with 7–9 representing high quality scores, 4–6 representing medium scores, and 1–3 representing low scores.^24^ For cross‐sectional study, adapted version of the NOS was used. More details are depicted in Table S3.

### Data management

The data extraction was done by two researchers (Mohammad Jahid Hasan, Md Abdullah Saeed Khan) using a standardized and pretested format. Data extraction included: title, first author, publication year, study design, settings (hospital‐based or population‐based), sample size, ethnicity, participant groups (lean *vs* nonlean, NAFLD *vs* non‐NAFLD), effect estimates for age, WC, BMI, systolic blood pressure, diastolic blood pressure, fasting blood sugar (FBS), HbA1c, insulin resistance (HOMA‐IR), total cholesterol (TC), high‐density lipoprotein (HDL), low‐density lipoprotein (LDL), triglycerides (TG), and uric acid (UA). Besides this, the search results and necessary notes were uploaded and managed using Microsoft Excel 2016.

### Statistical analysis

To summarize effects sizes a random effect model was adopted to allow for the variability of measurements across the studies included in the analysis. To specifically provide measures of the absolute difference between the mean values of each variable of interest calculated for any two groups (e.g. lean‐NAFLD *vs* lean non‐NAFLD patients, or lean‐NAFLD *vs* nonlean NAFLD, or lean non‐NAFLD *vs* nonlean non‐NAFLD), we used the difference in means. All the data presented as median (range), and median (interquartile range [IQR]) were converted to mean (SD) using an online calculator^25^ particularly using equations by Luo *et al*.^26^ and Wan *et al*.^27^ We measured the variables of interest on the same scale/unit. Results from studies that report laboratory data on SI units were converted to conventional units using appropriate conversion factors. For each analysis, a forest plot was generated to display results. Subgroup analysis across ethnicity, and study population were conducted to assess sources of heterogeneity. Corresponding forest plots with estimates of effects sizes across groups are presented in Supporting Information. Funnel plots were drawn to assess publication bias for risk factors which was reported in 10 or more studies. Details regarding subgroup analyses, meta‐regression, heterogeneity, and publication bias are fully disclosed in Supporting Information. The meta‐analysis was conducted using the statistical software “R” version 3.6.0 for Windows version 10 (Microsoft corporation, Redmond, WA, USA).

## Results

### Study identification and selection

**Figure 1:**
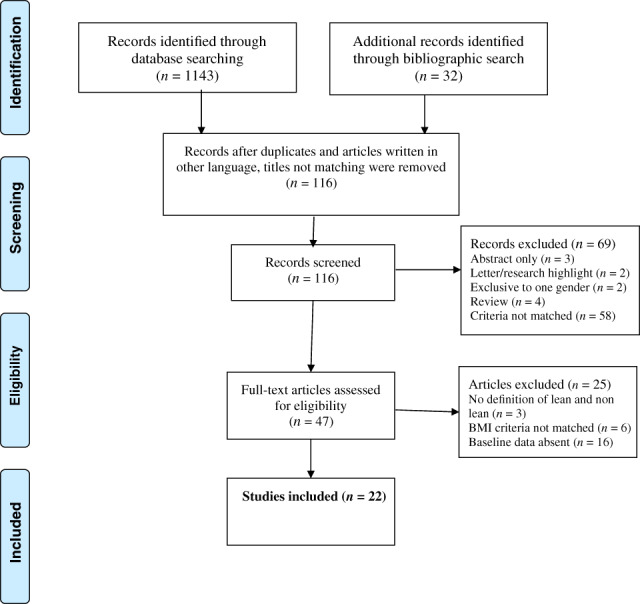
PRISMA 2009 flow diagram. BMI, body mass index.

The PRISMA flow chart visualizes the overall study screening process (Fig. 1). Initially 1175 articles were identified through search strategy which was narrowed down to 47 articles that matched the purpose of the study. After further scrutiny, 22 articles were finally selected for inclusion (Table S5 enlists the 25 articles which were excluded with main reasons for exclusion). Among the included articles, there were 19 cross‐sectional studies,^11^, ^16^, ^17^, ^28^, ^29^, ^30^, ^31^, ^32^, ^33^, ^34^, ^35^, ^36^, ^37^, ^38^, ^39^, ^40^, ^41^, ^42^, ^43^ 1 case–control study,^44^ and 2 cohort studies.^12^, ^45^

### Study characteristics

Detailed study characteristics for the 22 included studies are outlined in Table S4. Total sample size was 69 038 (lean NAFLD = 6768, lean non‐NAFLD = 43 398, nonlean NAFLD = 9253, and nonlean non‐NAFLD = 9619). All the studies included adults of both sexes.

Fifteen studies were conducted in hospital^11^, ^12^, ^29^, ^30^, ^31^, ^33^, ^36^, ^37^, ^39^, ^40^, ^41^, ^42^, ^43^, ^45^ and seven studies were conducted in the community setting.^16^, ^17^, ^28^, ^32^, ^34^, ^35^, ^38^, ^44^

Fatty liver was assessed by ultrasonography (USG) of liver in 15 studies^11^, ^17^, ^28^, ^29^, ^32^, ^33^, ^34^, ^35^, ^36^, ^37^, ^38^, ^39^, ^41^, ^42^, ^43^ by computed tomography scan in 1 study,^44^ by magnetic resonance spectroscopy in 1 study,^16^ and by percutaneous liver biopsy in 5 studies.^13^, ^30^, ^31^, ^40^, ^45^

Eight studies were of Caucasian origin^11^, ^17^, ^30^, ^31^, ^38^, ^39^, ^44^, ^45^ and 14 studies were of East‐Asian origin.^11^, ^12^, ^16^, ^17^, ^28^, ^29^, ^32^, ^33^, ^34^, ^36^, ^37^, ^38^, ^39^, ^41^, ^42^, ^43^, ^44^

Two studies^4^, ^6^ presented values as median (range), five studies^14^, ^15^, ^16^, ^19^, ^21^ presented as median (IQR), and rest of the studies^1^, ^2^, ^3^, ^5^, ^7^, ^8^, ^9^, ^10^, ^11^, ^12^, ^13^, ^17^, ^18^, ^20^, ^22^ presented as mean (SD). Median values were converted to mean using procedures described in Methods section.

### Meta‐analysis results

#### Age and risk of NAFLD in lean people

**Figure 2:**
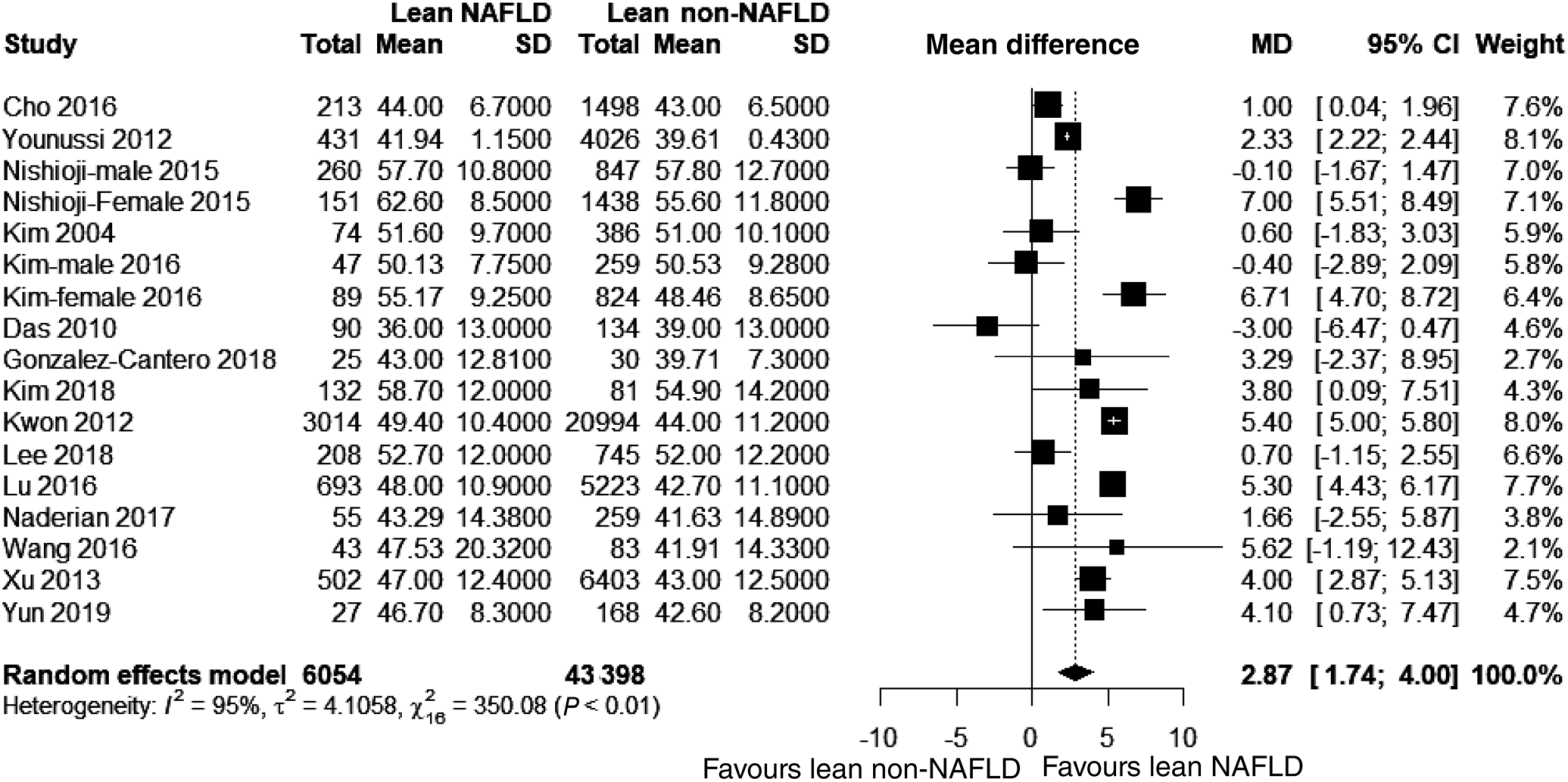
Forest plot for age. CI, confidence interval; MD, mean difference; NAFLD, nonalcoholic fatty liver disease.

For risk of NAFLD in lean individual, 15 studies^17^, ^28^, ^29^, ^30^, ^32^, ^33^, ^34^, ^35^, ^36^, ^37^, ^38^, ^39^, ^40^, ^41^, ^42^, ^43^, ^44^ compared age of lean NAFLD and non‐NAFLD participants. Among these, two studies^33^, ^35^ presented comparison separately for male and female. Only one study was case–control study,^44^ and rest of the studies were cross‐sectional studies.^11^, ^12^, ^16^, ^17^, ^28^, ^29^, ^30^, ^31^, ^32^, ^33^, ^34^, ^35^, ^36^, ^37^, ^38^, ^39^, ^40^, ^41^, ^42^, ^43^, ^45^ Figure 2 shows that lean NAFLD patients were significantly older than lean non‐NAFLD participants (mean difference [MD] 2.87 years, 95% confidence interval [CI] 1.74–4.00). The studies were considerably heterogenous (*I* ^2^ = 95%). Hence, a random effect model was used. Subgroup analysis across ethnicity showed that studies of Korean, Japanese, and Chinese origin (*I* ^2^ *=* 94, 98 and 85% respectively) were more heterogenous than that of Caucasian origin (*I* ^2^ *=* 68%) (Figure S2A). Studies stratified by study population (population‐based *vs* hospital‐based) did not show any significant change in heterogeneity (Figure S2B). However, a stratification across geographic location (Eastern *vs* other) showed less heterogeneity in other studies (*I* ^2^ *=* 93 *vs* 68% respectively) (Figure S2C).

#### BMI and risk of NAFLD in lean people

**Figure 3:**
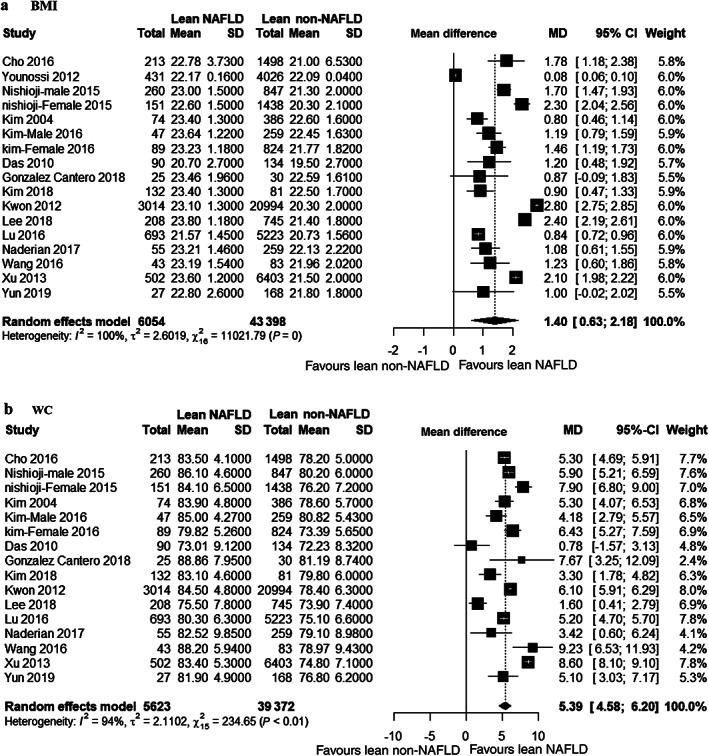
Forest plot for body mass index (BMI) (a) and waist circumference (WC) (b). CI, confidence interval; MD, mean difference; NAFLD, nonalcoholic fatty liver disease.

For risk of NAFLD in lean people, same number of studies as that of age compared BMI between lean NAFLD patients and lean non‐NAFLD controls. Figure 3a shows that lean NAFLD patients had significantly higher BMI than lean non‐NAFLD participants (MD 1.40 kg/m^2^, 95% CI 0.63–2.18). The studies were considerably heterogenous (*I* ^2^ = 100%). Subgroup analysis across ethnicity did not show any change in heterogeneity (Figure S3A). Studies stratified by population (population‐based *vs* hospital‐based) and by geographic location (Eastern *vs* other) showed similar heterogeneity (Figure S3B and S3C).

#### WC and risk of NAFLD in lean people

For risk of NAFLD in lean people, same number of studies as that of age compared WC between lean NAFLD patients and lean non‐NAFLD controls except one cross‐sectional study.^17^ Figure 3b shows that lean NAFLD patients had significantly higher WC than lean non‐NAFLD participants (MD 5.39 cm, 95% CI 4.58–6.20). These studies were considerably heterogenous (*I* ^2^ = 94%). Subgroup analysis across ethnicity showed that Chinese studies explained the most heterogeneity (*I* ^2^ = 98%). Korean (*I* ^2^ = 78%) and Caucasian (*I* ^2^ = 74%) studies were less heterogenous than that of Japanese (*I* ^2^ = 89%) and Chinese studies (Figure S4A). Studies stratified by population (population‐based *vs* hospital‐based) had similar high heterogeneity (Figure S4B). But, a stratification across geographic location (Eastern *vs* other) showed less heterogeneity in other studies (*I* ^2^ *=* 94 *vs* 74%, respectively) (Figure S4C).

#### Blood pressure and risk of NAFLD in lean people

**Figure 4:**
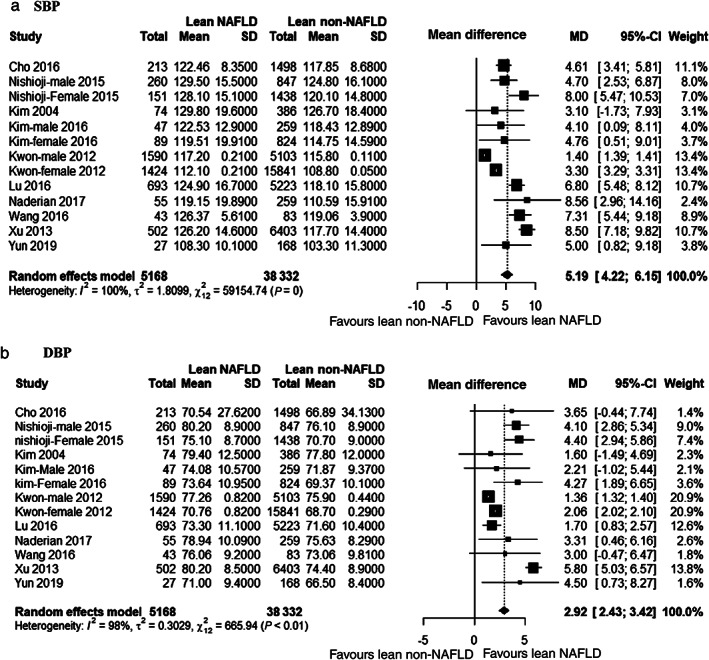
Forest plot for systolic blood pressure (SBP) (a) and diastolic blood pressure (DBP) (b).

Ten studies^28^, ^29^, ^32^, ^33^, ^34^, ^35^, ^36^, ^37^, ^38^, ^43^ compared blood pressure (BP) (both systolic blood pressure [SBP] and diastolic blood pressure [DBP]) of lean NAFLD and lean non‐NAFLD participants. Among those, three studies^29^, ^33^, ^35^ presented comparison separately for male and female. All the studies were cross‐sectional. Figure 4a,b shows that lean NAFLD patients had significantly higher BP than lean non‐NAFLD participants (SBP: MD 5.39 mmHg, 95% CI 4.58–6.20; DBP: MD 2.92 mmHg, 95% CI 2.43–3.42) with considerable heterogeneity (*I* ^2^ = 94% for SBP and 98% for DBP). Ethnicity showed that Chinese studies were relatively homogenous for SBP (*I* ^2^ = 39%, *P* = 0.19) than other. Heterogeneity was mostly explained by Korean studies (*I* ^2^ = 100%) (Figure S5A). Heterogeneity of DBP was explained by both Korean (*I* ^2^ = 99%) and Chinese (*I* ^2^ = 96%) studies (Figure S6A). Studies stratified by settings showed population‐based studies were less heterogenous than hospital‐based (*I* ^2^ = 77 *vs* 100% respectively for SBP and 63 *vs* 99% respectively for DBP) (Figure S5B and S6B). However, stratification across geographic location (Eastern *vs* others) did not reveal any difference as only one study in the latter group reported BP (Figure S5C and S6C).

#### FBS and risk of NAFLD in lean people

**Figure 5:**
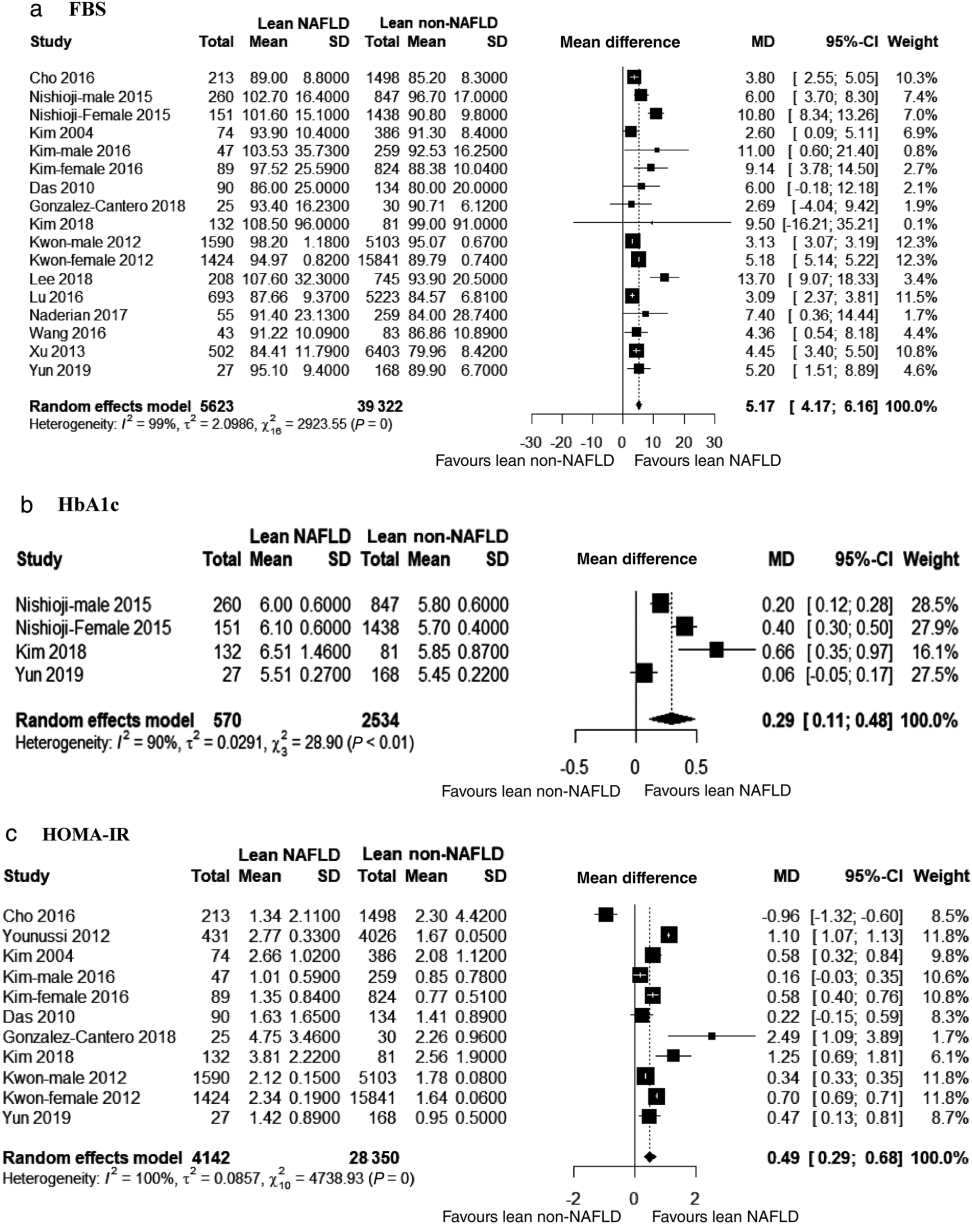
Forest plot for fasting blood sugar (FBS) (a), HbA1c (b), and (c) insulin resistance (HOMA‐IR).

Fifteen studies compared FBS between lean NAFLD patients and lean non‐NAFLD controls. Figure 5a shows that lean NAFLD patients had significantly higher FBS than lean non‐NAFLD controls (MD 5.17 mg/dL, 95% CI 4.17–6.16). These studies were considerably heterogenous (*I* ^2^ = 99%). Subgroup analysis across ethnicity showed that heterogeneity was mostly explained by Korean (*I* ^2^ = 100%), Japanese (*I* ^2^ = 87%), and Chinese (*I* ^2^ = 87%) studies (Figure S7A). Heterogeneity of the studies can be explained by hospital‐based studies and Eastern studies (Figure S7B and S7C).

#### HbA1c and risk of NAFLD in lean people

Only three studies^33^, ^40^, ^43^ compared HbA1c between lean NAFLD and lean non‐NAFLD controls. One study^33^ compared HbA1c by sex. Figure 5b shows that lean NAFLD patients had significantly higher HbA1c than lean non‐NAFLD controls (MD 0.29%, 95% CI 0.11–0.48). These studies were considerably heterogenous (*I* ^2^ = 90%). Subgroup analysis by ethnicity showed similar heterogeneity (Figure S8A).

#### HOMA‐IR and risk of NAFLD in lean people

Nine studies^17^, ^28^, ^29^, ^34^, ^35^, ^39^, ^40^, ^43^, ^44^ compared HOMA‐IR of lean NAFLD and non‐NAFLD participants. One study^35^ presented gender difference, and one^44^ was case–control study. Figure 5c shows that lean NAFLD patients had significantly higher HOMA‐IR than lean non‐NAFLD controls (MD 0.49 U, 95% CI 0.29–0.68). These studies were considerably heterogenous (*I* ^2^ = 100%). Subgroup analysis did not show any difference in heterogeneity across any stratification (Figure S9A–C).

#### Serum lipid profile and risk of NAFLD in lean people

**Figure 6:**
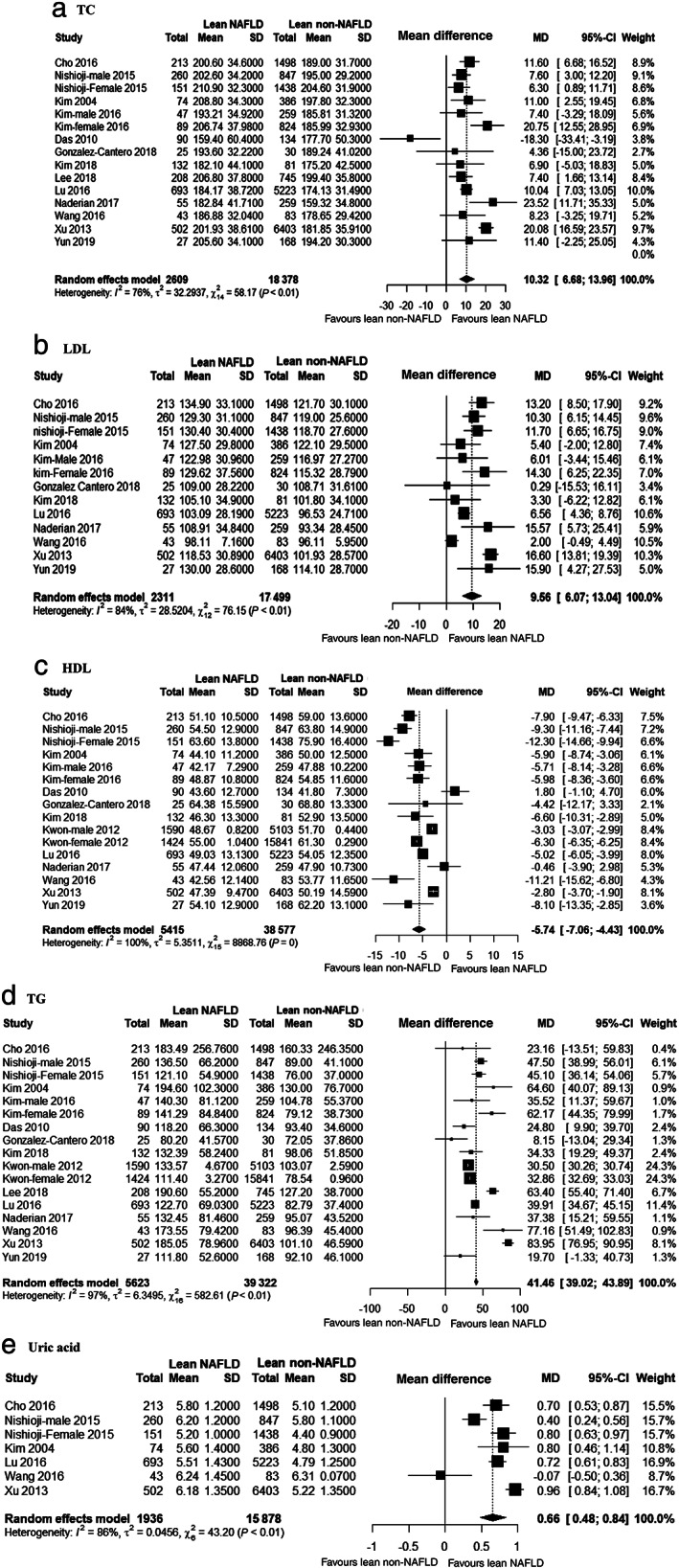
Forest plot for total cholesterol (TC) (a), low‐density lipoprotein (LDL) (b), high‐density lipoprotein (HDL) (c), triglyceride (TG) (d), and uric acid (e).

For risk of NAFLD in lean people, 13 studies^28^, ^32^, ^33^, ^34^, ^35^, ^36^, ^37^, ^38^, ^39^, ^40^, ^41^, ^43^, ^44^ compared TC of lean NAFLD and non‐NAFLD participants. Same studies except two,^41^, ^44^ except one,^41^ and plus one^29^ study also compared LDL, HDL, and TG between those groups, respectively. Only two studies^33^, ^35^ presented comparison separately for male and female and one study was case–control.^44^ Figure 6a–d show that lean NAFLD patients had significantly higher TC, higher LDL, lower HDL, and higher TG than lean non‐NAFLD controls (TC: MD 10.32 mg/dL, 95% CI 6.68–13.96; LDL: MD 9.56 mg/dL, 95% CI 6.07–13.04; HDL: MD −5.74 mg/dL, 95% CI −7.06 to 4.43; and TG: MD 41.46 mg/dL, 95% CI 39.02–43.89). These studies were substantially heterogenous (*I* ^2^ = 76%, 76, 100 and 97% respectively for TC, LDL, HDL, and TG). Subgroup analysis by ethnicity is shown in Figures S10A, S11A, S12A, and S13A. Studies stratified by population (population‐based *vs* hospital‐based) showed that heterogeneity in TC is completely explained by population‐based studies (*I* ^2^ = 83%), but heterogeneity in LDL and HDL is explained by hospital‐based studies (*I* ^2^ *=* 74% each) (Figure S10B, S11B, and S12B). However, it was same across study population in case of TG (Figure S13B). Stratification across geographic location (Eastern *vs* other) showed that Eastern studies were overall more heterogenous than other studies for LDL, HDL, and TG and other way around for TC (Figures S10C, S11C, S12C, and S13C).

#### Serum UA and risk of NAFLD in lean people

For risk of NAFLD in lean people, only six studies^28^, ^32^, ^33^, ^34^, ^36^, ^38^ compared UA between lean NAFLD patients and lean non‐NAFLD controls. One study^33^ compared UA by sex. All were cross‐sectional studies. Figure 6e showed that lean NAFLD patients had significantly higher UA than lean non‐NAFLD controls (MD 0.66 mg/dL, 95% CI 0.48–0.84). These studies were considerably heterogenous (*I* ^2^ = 86%). Subgroup analysis by ethnicity and study settings are shown in Figures S14A and S14B.

#### *Liver function tests in lean NAFLD* versus *non‐NAFLD*

Alanine aminotransferase (ALT) was reported in 13 studies.^17^, ^28^, ^29^, ^33^, ^34^, ^35^, ^36^, ^37^, ^38^, ^39^, ^40^, ^41^, ^43^ Aspartate aminotransferase (AST) was reported in 10 studies^17^, ^28^, ^29^, ^33^, ^34^, ^37^, ^38^, ^39^, ^40^, ^41^, ^43^ and gamma glutamyl transferase (GGT) was reported in 10 studies.^28^, ^29^, ^32^, ^33^, ^34^, ^35^, ^36^, ^37^, ^39^, ^40^, ^41^, ^43^ Compared to non‐NAFLD controls lean NAFLD patients had significantly higher mean values of ALT (MD 8.12 U/L, 95% CI 6.21–10.02, Figure S15A), AST (MD 2.72 U/L, 95% 1.91–3.63, Figure S16A), and GGT (MD 11.21 U/L, 95% CI 9.02–13.40, Figure S17A). Analysis for all three variables were considerably heterogenous. Subgroup analysis showed that heterogeneity is mostly explained by Korean studies for ALT, Caucasian studies for AST, and Chinese studies for GGT (Figures S15B, S16B, and S17B). Whereas subgroup analysis by population (population‐based *vs* hospital‐based) showed that heterogeneity was mainly due to hospital‐based studies (Figures S15C, S16C, and S17C). Subgroup analysis by Eastern *versus* other studies showed that heterogeneity was higher among Eastern studies for ALT and higher among other studies for AST (Figures S15D, S16D, and S17D).

#### Summary of the findings

A summary of the meta‐analysis results along with subgroup analysis for individual demographic, anthropometric, and cardiometabolic risk factors for NAFLD in lean people are given in Tables S6‐S8. All the studies were considerably heterogenous and heterogeneity varied by ethnicity, study population, and risk factor under consideration. Random effect model analysis showed that all of the factors had significantly different effects estimates between lean NAFLD patients and non‐NAFLD controls.

#### Publication bias

For risk factors with 10 or more studies, funnel plot analysis was conducted (Figure S18). Almost all the plots showed asymmetry which could be explained mostly by heterogeneity of the studies and partly by biased reporting of studies with significant effect sizes. A visual inspection of the funnel plots shows that studies reporting SBP, DBP, GGT, TC, and LDL were moderately asymmetric. While other risk factors showed a high asymmetry.

#### Comparison of risk factors of nonlean and lean NAFLD

A summary of differences in effect size between lean and nonlean NAFLD patients, and lean and nonlean controls are given in Table 1. Nonlean NAFLD patients had significantly different effect estimates than lean NAFLD patients in cardio metabolic profiles: BMI (mean difference, *P*‐value: 5.62 kg/m^2^, *P* < 0.001), WC (16.44 cm, *P* < 0.001), SBP (4.97 mmHg, *P* < 0.001), DBP (3.45 mmHg, *P* < 0.001), FBS (3.37 mg/dL, *P* < 0.001), HbA1c (0.14%, *P* = 0.02), HOMA‐IR (1.07, *P* < 0.001), HDL (−3.12 mg/dL, *P* < 0.001), TG (13.07 mg/dL, *P* = 0.001), UA (0.37 mg/dL, *P* = 0.003), ALT (5.66 U/L, *P* < 0.001), AST (3.31 U/L, *P* < 0.001), and GGT (4.58 U/L, *P* = 0.027). Similarly nonlean controls had significantly different values in cardiometabolic risk than lean control: BMI (5.25 kg/m^2^, *P* < 0.001), WC (11.36 cm, *P* < 0.001), SBP (6.71 mmHg, *P* < 0.001), DBP (3.64 mmHg, *P* < 0.001), FBS (2.91 mmol/L, *P* = 0.003), HbA1c (0.06%, *P* = 0.044), HOMA‐IR (0.53, *P* = 0.009), TC (5.13 mg/dL, *P* = 0.001), LDL (6.25 mg/dL, *P* < 0.001), HDL (−5.67, *P* < 0.001), TG (22.36, *P* < 0.001), UA (0.36 mg/dL, *P* = 0.007), ALT (2.71 U/L, *P* < 0.001), AST (3.31, *P* < 0.001), and GGT (4.58, *P* = 0.027). In addition, nonlean controls were significantly older than lean controls (2.63 years, *P* < 0.001).

**Table 1:** Meta‐regression analysis to examine the impact of nonalcoholic fatty liver disease (NAFLD) on the risk factors

Outcome	NAFLD lean *versus* nonlean				Non‐NAFLD lean *versus* nonlean				Meta‐regression analysis	
	Mean difference (95% CI)	*P*‐value	Sample size	No of sub studies	Mean difference (95% CI)	*P*‐value	Sample size	No of sub studies	Slope	*P*‐value
Age	0.12 [−2.72; 2.96]	0.934	9212/5385	19	2.63 [1.71; 3.55]	<0.001	9576/29717	10	−0.06	0.839
BMI	5.62 [3.66; 7.58]	<0.001	8565/5231	17	5.25 [4.58; 5.93]	<0.001	9576/29717	10	1.12	**<0.001**
WC	16.44 [13.87; 19.00]	<0.001	7192/5044	20	11.36 [9.72; 13.00]	<0.001	1470/4697	8	1.15	**<0.001**
SBP	4.97 [3.25; 6.69]	<0.001	6142/4543	14	6.71 [3.68; 9.73]	<0.001	3683/24688	8	0.09	0.459
DBP	3.45 [2.76; 4.13]	<0.001	6142/4543	14	3.64 [2.21; 5.07]	<0.001	3683/24866	8	0.19	**0.037**
FBS	3.37 [2.44; 4.30]	<0.001	7151/4954	19	2.91 [1.34; 4.47]	0.003	4481/25641	10	0.19	0.064
HbA1c	0.14 [0.02; 0.25]	0.021	1661/814	7	0.06 [0.00; 0.13]	0.044	205/2453	3	0.00	0.993
HOMA‐IR	1.07 [0.57; 1.57]	<0.001	8155/4675	15	0.53 [0.13; 0.92]	0.009	8625/26637	8	0.58	**<0.001**
TC	0.49 [−1.71; 2.70]	0.660	4126/1940	17	5.13 [2.08; 8.18]	0.001	1470/4697	8	0.13	0.226
LDL	0.58 [−0.99; 2.14]	0.470	3841/1732	16	6.25 [2.96; 9.54]	<0.001	700/3952	7	0.08	0.244
HDL	−3.12 [−3.74; −2.49]	<0.001	6866/4746	18	−5.67 [−6.94; −4.40]	<0.001	3711/24896	9	−0.01	0.964
TG	13.07 [5.36; 20.77]	0.001	7151/4954	19	22.36 [18.99; 25.73]	<0.001	4481/25641	10	0.02	0.910
UA	0.37 [0.13; 0.61]	0.003	1846/1022	6	0.36 [0.10; 0.63]	0.007	383/2671	3	0.36	**0.022**
ALT	5.66 [3.59; 7.72]	<0.001	9212/5385	19	2.71 [1.03; 4.40]	0.002	9576/29717	10	0.11	0.233
AST	3.31 [2.32; 4.30]	<0.001	8716/5041	16	0.33 [−0.81; 1.48]	0.569	8541/27889	7	0.27	**0.003**
GGT	4.58 [0.52; 8.65]	0.027	5096/4123	12	6.75 [2.30; 11.21]	0.003	3509/24560	7	0.32	**0.019**

ALT, alanine aminotransferase; AST, aspartate aminotransferase; BMI, body mass index; CI, confidence interval; DBP, diastolic blood pressure; FBS, fasting blood sugar; GGT, gamma glutamyl transferase; HDL, high‐density lipoprotein; HOMA‐IR, insulin resistance; LDL, low‐density lipoprotein; SBP, systolic blood pressure; TC, total cholesterol; TG, triglycerides; UA, uric acid; WC, waist circumference.

The differences in values of individual risk factors between lean and nonlean patients and controls could be explained by the significant differences in BMI and WC. However, the meta‐regression analysis showed that the magnitude of the difference in the value of the lipid profile and glycemic profile barely reached statistical significance when subjects were grouped according to the presence or absence of NAFLD (Table 1). But for cardio metabolic variables, the magnitude of effect was significantly higher if NAFLD was present compared to that of non‐NAFLD group. These are: DBP (slope: 0.19, *P* < 0.037), HOMA‐IR (slope: 0.58, *P* < 0.001), UA (slope: 0.36, *P* = 0.022). Also, the presence of NAFLD is associated with significantly higher values of AST (slope: 0.27, *P* = 0.003) and GGT (slope: 0.32, *P* = 0.019) in blood.

## Discussion

NAFLD in lean persons is now a widely recognized problem. Initially, described in Asian populations and considered as a “third world phenotype,” this subset of NAFLD has since been described in other populations, including in Europe and the United States.^20^, ^46^ Therefore, risk factors associated with lean NAFLD are being studied worldwide. A previous metanalysis, which synthesized evidence based on research studies published up to 2016, found that risk factors of NAFLD are shared between lean and obese individuals.^47^ Since then more data have been generated, especially the importance of uric acid, which was found to have a significant positive nonlinear association with risk of cardiovascular disease (CVD) mortality (Hazard Ratio [HR] 1.45, 95% CI 1.33–1.58, *I* ^2^ = 79%)^48^ as a risk factor of NAFLD was being studied. Hence, an updated metanalysis was warranted.

Our study found that lean NAFLD patients were significantly older than lean non‐NAFLD controls. This is different from that found by Sookoian *et al*.^47^ The difference in age varied across the studies. Usually, studies with small sample sizes^28^, ^35^, ^37^, ^38^, ^39^, ^40^, ^41^, ^44^ reported small nonsignificant difference in age while studies with large sample sizes^17^, ^32^, ^33^, ^44^ found a significant difference. Finally, a pooled estimate produced a significant effect size. Hamaguchi *et al*. studied the effect of aging on NAFLD in detail and found that aging is a significant nonmodifiable risk factor for NAFLD in premenopausal women independent of weight gain and metabolic syndrome.^49^ We found that lean NAFLD patients were 2.87 years (95% CI 1.74–4.00, *P* < 0.01) older than lean non‐NAFLD individuals, while this difference was reported to be 3.79 (95% CI 2.38–5.20) years in the previous study.^47^ In addition, we found that the differences were more in hospital‐based studies (3.86; 95% CI 2.29–5.43) in comparison to population‐based studies (1.99; 95% CI 0.74–3.25). Interestingly, the difference was mainly due to Eastern studies, because other non‐Eastern studies did not find a significant difference of age between these two groups. These suggest that lean individuals are more likely to develop NAFLD with an increasing age. Although age cannot be modified or treated, lean individuals who have other concomitant risk factors of NAFLD should be actively monitored and sought for presence of fatty liver with an increasing age, so that they can be included in management strategies as early as possible.

All components of metabolic syndrome (high BP, hyperglycemia, insulin resistance, visceral adiposity, and lipid profile) along with UA were present in both lean and nonlean NAFLD patients. Even in lean patients, NAFLD was characterized by the presence of significantly higher BMI and WC than non‐NAFLD controls. WC is a surrogate of visceral obesity.^50^ Excess free fatty acid (FFA) released from visceral adipose tissue leads to over‐exposure of FFA to hepatic and extra‐hepatic tissue promoting aberrations in insulin action and dynamics.^51^ Moreover, FFA was found to be significantly higher in NAFLD patients than non‐NAFLD controls irrespective of BMI.^52^ This also explains the benefit of weight reduction in lean NAFLD patients.^21^

FBS showed less heterogeneity among Caucasian people and in population‐based studies. Most of the heterogeneity in FBS was explained by Asian, and hospital‐based studies. HbA1c, BP (SBP and DBP), lipid profiles (TC, LDL, HDL, and TG), and UA showed considerable heterogeneity across different ethnicity and study design. The random effect model analysis showed that all these variables were significantly higher in lean NAFLD patients than non‐NAFLD controls (except HDL which was significantly lower). These findings are consistent with that of Sookoian and Pirola^14^ reported nearly similar differences in risk factors between lean NAFLD and non‐NAFLD individuals. Unlike them, we did a subgroup analysis based on the population and geographic region (Eastern *vs* others) where some interesting findings were noted. A significantly higher mean difference was noted in BMI, WC, HOMA‐IR, ALT, and AST for hospital‐based studies and in BP, lipid profiles, uric acid, and GGT for population‐based studies. Several possible reasons could explain this difference. As some of the participants in hospital were more likely to have some sorts of illness or comorbidities, they might have been taking medications for BP, lipid profile, or even uric acid. This may explain the lower values of these pooled parameters in the hospital‐based population. While the anthropometric parameters, which do not change immediately on medication and which are expected to be higher in symptomatic NAFLD individuals coming to take health care service, were found high in the hospital‐based population. On the other hand, nearly all the parameters showed a higher mean difference in Eastern studies rather the non‐Eastern ones. Although overall population of the Eastern studies were highly heterogenous, the ethnic characteristics shared by Japanese, Chinese, and Korean population might explain the difference than other studies comprising Indian, Iranian, and American population of mixed ethnicity. Overall, our findings suggest that if lean people have any suspicious rise of these risk factors' parameters, they should be screened for NAFLD. Also, lean‐NAFLD patients with symptoms and/or comorbidities need to be treated aggressively and monitored closely for compliance, and lean people of Eastern origin if screened positive for NAFLD should be given proper attention as required.

Uric acid was found to be an independent risk factor for NAFLD in lean person by several other studies conducted in Iran^53^ and China.^54^, ^55^ It is reportedly responsible for lipid metabolism impairment and inflammation.^56^, ^57^, ^58^ Thus, there may be interaction between high serum UA and increased weight in the pathogenesis of NAFLD. Hence, all suspected or diagnosed lean NAFLD patients should undergo UA assay and specified management for UA. Further studies on lean NAFLD patients must include UA as a part of laboratory tests.

The meta‐regression analysis showed that the presence of higher DBP, HOMA‐IR, and UA in both lean and nonlean patients were independently associated with NAFLD irrespective of BMI and WC. Therefore, NAFLD could be considered an important but separate component within the spectrum of metabolic syndrome which interacts with other components and influences each other. This hypothesis is strengthened by several other studies where the authors suggest that NAFLD works as a precursor of metabolic syndrome^59^ independent of central obesity and insulin resistance.^60^ Besides our findings suggest that treatment of NAFLD, particularly in lean patients, should involve uncompromising management of diastolic blood pressure, insulin resistance, and uric acid.

Taken together, patients with lean NAFLD, who do not have any infectious‐inflammatory and drug cause^46^ may have additional pathogenetic mechanisms other than metabolic causes of NAFLD. Some of these mechanisms are as follows. At least four genetic variants have shown robust association with development and progression of NAFLD.^61^ These are: *PNPLA3* (rs738409 C > G), *TM6SF2* (rs58542926 C > T), *MBOAT7* (rs641738 C > T), and *GCKR* (rs1260326 C > T). However, there is a paucity of literature regarding specific genetic markers among lean NAFLD patients. Mixed findings are reported regarding frequency of PNPLA3 and TM6SF2 variants when compared between lean and nonlean NAFLD patients.^62^, ^63^ Polymorphism in *IFNL3*, *CETP*, and *PEMT* has been shown to be associated with the progression of NAFLD among lean or nonobese individuals by several studies.^46^ Further studies are needed to find any strong association of genetic variants with NAFLD among lean population.

Fecal, gut, and blood microbiome has recently been brought into focus as probable risk factors for NAFLD in lean patients.^37^, ^43^, ^62^, ^64^, ^65^ It was considered because dysbiosis between beneficial and pathogenic bacteria may lead to obesity, insulin resistance, and NAFLD^65^ particularly in nonobese individual.^37^ Yun *et al*. found that NAFLD patients showed a distinct bacterial community with a lower biodiversity and a far distant phylotype compared to control group, and fecal and blood microbiota profiles showed different patterns between subjects with obese and lean NAFLD^43^ However, relationship between microbiota, and metabolic and inflammatory response is complex which warrants extensive investigation.

Several studies also found significant associations of serum sialic acid,^36^ bile acid,^62^ and apolipoproteins (apoB1 level and apoB/A1 ratio),^66^ with lean NAFLD which need further research for confirmation.

The meta‐regression results also indicated that serum levels of AST and GGT, but not ALT were markedly modulated by the presence of NAFLD. Sookoian and Pirola^14^ previously noted a similar finding except that it was only for AST. Accumulation of fat in liver causes increased energy demand and in response to that synthesis of transaminase, particularly the mitochondrial isoform AST, increases.^47^ On the other hand, Hossain *et al*.^67^ proposed that GGT is an independent determinant of association of insulin resistance with nonalcoholic fatty liver disease in adults. Our result endorses their findings.

Finally, the comparison of risk factors between lean *versus* nonlean NAFLD in our study found that almost all the components were present in significantly higher amount among nonlean subjects. A higher BMI and visceral obesity may explain this difference. It is obvious that nonlean (overweight/obese) NAFLD patients are in need of stringent management of weight and other metabolic parameters. However, our study results emphasize the importance of this strategy for both lean and nonlean NAFLD patients.

To summarize, lean and nonlean NAFLD patients share similar cardiovascular and metabolic risk factors. Genetic, microbiological, and other metabolic factors warrant further investigation to identify unique determinants of NAFLD in lean individuals. Regardless of the underlying mechanisms, lean NAFLD needs to be given appropriate clinical attention similar to that of nonlean NAFLD.

### Limitations and strengths

The current study was limited in several aspects. Selected articles were mostly heterogenous and mostly it was due to the inclusion of the studies from different ethnic origin as well as inclusion of both hospital‐ and population‐based studies. Even within a unique ethnic setting, separate studies were considerably heterogenous with respect to different variables under consideration. This indicates remarkable individual differences in risk factors within same ethnic population. Also, a separate analysis for different BMI cut‐off points used in definition of lean and nonlean was not possible. Therefore, implication of the risk factors of NAFLD with respect to Asian population, where a different BMI cut‐off point for overweight and obesity often used could not be determined. Many authors did not report separate analysis for male and female. Therefore, a sex‐based subgroup analysis was not possible. Multivariate analysis to determine independent associations between risk factors and lean‐NAFLD could not be done, because of unavailability of data, leading to an inference of only general associations among different risk factors and NAFLD.

The strength of the study was its large sample size of 69 038 individuals, comprising 6768 lean NAFLD patients, 9253 nonlean NAFLD patients, 43 398 lean controls, and 9619 nonlean controls. Also, consistent findings across different cardiovascular and metabolic risk factors strengthened our results. Hope that the study could be used as an update of previous metanalysis in this topic and will be evident for future clinicians as well as policy makers.

### Recommendations for future studies

NAFLD could be considered a part of an interactome of metabolic syndrome components working in a coordinated fashion to develop chronic diseases and complications involving different systems of the body. Therefore, to incorporate NAFLD within the definition of metabolic syndrome extensive research is necessary. Well‐designed case–control and cohort studies for risk factor analysis of both lean and non‐NAFLD are lacking. Multivariate analysis of risk factors is also warranted. Further explorative research on unique determinants of lean‐NAFLD are recommended.

In conclusion, Age, BMI, WC, BP, FBS, HbA1c, insulin resistance, uric acid, TC, LDL, HDL, and TG are the important risk factors of NAFLD shared equally among lean and nonlean. In other words, lean and nonlean NAFLD are anthropometrically different but metabolically similar entities. The findings provide a scientific basis for a further understanding of the nature of NAFLD in lean individual, and might provide a guide to determine the management strategies for lean‐NAFLD.

## Supporting information


**Appendix** **S1.** Supporting information.
