# Supplementary material for: Risk factors of nonalcoholic fatty liver disease in lean body mass population: A systematic review and meta‐analysis
Source: JGH Open. 2021 Oct 4;5(11):1236–49. doi: 10.1002/jgh3.12658 (PMC8593777; doi:10.1002/jgh3.12658)
Supplement: Supplementary file 1 — Appendix S1. Supporting information. [file JGH3-5-1236-s001.docx]

**SUPPLEMENTARY** **MATERIAL**

**Study type: Meta-analysis**

**Title: Risk factors of Non-alcoholic fatty liver disease (NAFLD) in lean body mass population: a systematic review and meta-analysis**

**Contributors:**

Shahinul Alam^1^, Mohammed Eslam^2^, SKM Nazmul Hasan^3^, Kamrul Anam^4^, Muhammad Abdul Baker Chowdhury^5^, Md. Abdullah Saeed Khan^6^, Mohammad Jahid Hasan^7^, Rosmawati Mohamed^8^

| **Table/Figure no.** | **Title of the table/figure** | **Page no.** |
| --- | --- | --- |
| Supplementary Table 1. | PRISMA checklist | 5 |
| Supplementary table 2 | Searching History | 7 |
| Supplementary Table 3 | Study type and quality assessment of the studies | 8 |
| Supplementary Table 4 | Characteristics of studies included in the analysis | 9 |
| Supplementary Table 5 | Characteristics of relevant studies excluded from the analysis with main reasons for exclusion | 17 |
| Supplementary Table 6 | Lean non-NAFLD vs NAFLD- Summary of overall effect and test for heterogeneity for all variables | 27 |
| Supplementary Table 7 | Lean non-NAFLD vs NAFLD- Summary of subgroup analysis across type of study population | 28 |
| Supplementary Table 8 | Lean non-NAFLD vs NAFLD- Summary of subgroup analysis across ethnicity | 30 |
| Supplementary Table 9 | Lean non-NAFLD vs NAFLD- Summary of subgroup analysis across Eastern and other studies. | 32 |
| Supplementary Figure 1 | Forest plot for age. | 35 |
| Supplementary Figure 2A | Forest plot for age- Subgroup Analysis by ethnicity | 36 |
| Supplementary Figure 2B | Forest plot for age- Subgroup analysis by study population | 37 |
| Supplementary Figure 2C | Forest plot for age- Subgroup Analysis by Eastern and other studies | 38 |
| Supplementary Figure 3 | Forest plot for BMI | 39 |
| Supplementary Figure 3A | Forest plot for BMI - subgroup analysis by ethnicity | 40 |
| Supplementary Figure 3B | Forest plot for BMI- subgroup analysis by study population | 41 |
| Supplementary Figure 3C | Forest plot analysis for BMI- Subgroup analysis by Eastern and other studies | 42 |
| Supplementary Figure 4 | Forest plot for waist circumference | 43 |
| Supplementary Figure 4A | Forest plot for waist circumference- Subgroup with respect to ethnicity. | 44 |
| Supplementary figure 4B | Forest plot for waist circumference- Subgroup with respect to population. | 45 |
| Supplementary figure 4C | Forest plot for waist circumference- Subgroup with respect to Eastern and other studies. | 46 |
| Supplementary Figure 5 | Forest plot for systolic blood pressure | 47 |
| Supplementary Figure 5A | Forrest plot analysis for Systolic Blood Pressure- Subgroup with respect to ethnicity. | 48 |
| Supplementary Figure 5C | Forrest plot analysis for Systolic Blood Pressure -Subgroup with respect to Eastern and other studies. | 49 |
| Supplementary Figure 5B | Forrest plot analysis for Systolic Blood Pressure -Subgroup with respect to population. | 50 |
| Supplementary Figure 6 | Forrest plot analysis for diastolic blood pressure | 51 |
| Supplementary Figure 6A | Forrest plot analysis for diastolic blood pressure- Subgroup analysis with respect to ethnicity | 52 |
| Supplementary Figure 6B | Forrest plot analysis for diastolic blood pressure- Subgroup with respect to population. | 53 |
| Supplementary Figure 6C | Forrest plot analysis for diastolic blood pressure- Subgroup with respect to Eastern and other studies. | 54 |
| Supplementary Figure 7 | Forest plot for fasting blood sugar | 55 |
| Supplementary figure 7A | Forrest plot analysis for fasting blood sugar- Subgroup with respect to ethnicity | 56 |
| Supplementary figure 7B | Forrest plot analysis for fasting blood sugar- Subgroup with respect to population | 57 |
| Supplementary figure 7C | Forrest plot analysis for fasting blood sugar- Subgroup with respect to Eastern and other studies. | 58 |
| Supplementary figure 8 | Forest plot for HbA1c | 59 |
| Supplementary figure 8A | Forrest plot for HbA1c- Subgroup analysis with respect to ethnicity | 60 |
| Supplementary figure 9 | Forest plot for HOMA-IR | 61 |
| Supplementary figure 9A | Forest plot for HOMA-IR- Subgroup with respect to ethnicity | 62 |
| Supplementary figure 9B | Forest plot for HOMA-IR-Subgroup with respect to population | 63 |
| Supplementary figure 9C | Forest plot for HOMA-IR-Subgroup with respect to Eastern and other studies. | 64 |
| Supplementary figure 10 | Forest plot for total cholesterol | 65 |
| Supplementary Figure 10A | Forrest plot for total cholesterol- Subgroup with respect to ethnicity. | 66 |
| Supplementary Figure 10B | Forrest plot for total cholesterol- Subgroup with respect to population. | 67 |
| Supplementary Figure 10C | Forrest plot for total cholesterol- Subgroup with respect to Eastern and other studies | 68 |
| Supplementary Figure 11 | Forrest plot for LDL | 69 |
| Supplementary Figure 11A | Forrest plot for LDL- Subgroup with respect to ethnicity | 70 |
| Supplementary Figure 11B | Forrest plot for LDL- Subgroup with respect to population | 71 |
| Supplementary Figure 11C | Forrest plot for LDL- Subgroup with respect to Eastern and other studies. | 72 |
| Supplementary Figure 12 | Forest plot for HDL | 73 |
| Supplementary Figure 12A | Forest plot for HDL - Subgroup with respect to ethnicity | 74 |
| Supplementary Figure 12B | Forest plot for HDL - Subgroup with respect to population | 75 |
| Supplementary Figure 12C | Forest plot for HDL - Subgroup with respect to Eastern and other studies. | 76 |
| Supplementary Figure 13 | Forest plot for TG | 77 |
| Supplementary Figure 13A | Forrest plot for TG - Subgroup with respect to ethnicity. | 78 |
| Supplementary Figure 13B | Forrest plot for TG - Subgroup with respect to population | 79 |
| Supplementary Figure 13C | Forrest plot for TG - Subgroup with respect to Eastern and other studies. | 80 |
| Supplementary Figure 14 | Forest plot for uric acid. | 81 |
| Supplementary Figure 14A | Forrest plot for uric acid- Subgroup with respect to ethnicity. | 82 |
| Supplementary Figure 14B | Forrest plot for uric acid- Subgroup with respect to population. | 83 |
| Supplementary Figure 15A | Forest plot for ALT. | 84 |
| Supplementary figure 15B | Forrest plot for ALT - Subgroup with respect to ethnicity | 85 |
| Supplementary figure 15C | Forrest plot for ALT- Subgroup with respect to population. | 86 |
| Supplementary figure 15D | Forrest plot for ALT- Subgroup with respect to Eastern and other studies. | 87 |
| Supplementary Figure 16A | Forest plot for AST. | 88 |
| Supplementary figure 16B | Forrest plot for AST- Subgroup with respect to ethnicity | 89 |
| Supplementary figure 16C | Forrest plot for AST- Subgroup with respect to population | 90 |
| Supplementary figure 16D | Forrest plot for AST- Subgroup with respect to Eastern and other studies. | 91 |
| Supplementary Figure 17A | Forest plot for GGT. | 92 |
| Supplementary figure 17B | Forrest plot for GGT- Subgroup with respect to ethnicity. | 92 |
| Supplementary figure 17C | Forrest plot for GGT- Subgroup with respect to population | 93 |
| Supplementary figure 17D | Forrest plot for GGT- Subgroup with respect to Eastern and Other studies | 94 |
| Supplementary Figure 18 | Funnel plots for different variables | 95 |
| Supplementary Figure 18 (continued). | Funnel plots different variables | 96 |

**Supplementary Table 1. PRISMA checklist**

| **Section/topic** | | **#** | **Checklist item** | | | **Reported on page #** |
| --- | --- | --- | --- | --- | --- | --- |
| **TITLE** | | | | | |  |
| **Title** | | **1** | **Identify the report as a systematic review, meta-analysis, or both.** | | | **1** |
| **ABSTRACT** | | | | | | **3-4** |
| Structured summary | | 2 | Provide a structured summary including, as applicable: background; objectives; data sources; study eligibility criteria, participants, and interventions; study appraisal and synthesis methods; results; limitations; conclusions and implications of key findings; systematic review registration number. | | | **3-4** |
| INTRODUCTION | | | | | | **4-5** |
| Rationale | | 3 | Describe the rationale for the review in the context of what is already known. | | | **5** |
| Objectives | | 4 | Provide an explicit statement of questions being addressed with reference to participants, interventions, comparisons, outcomes, and study design (PICOS). | | | **5** |
| METHODS | | | | | | **5-8** |
| Protocol and registration | | 5 | Indicate if a review protocol exists, if and where it can be accessed (e.g., Web address), and, if available, provide registration information including registration number. | | | **5** |
| Eligibility criteria | | 6 | Specify study characteristics (e.g., PICOS, length of follow-up) and report characteristics (e.g., years considered, language, publication status) used as criteria for eligibility, giving rationale. | | | **6-7** |
| Information sources | | 7 | Describe all information sources (e.g., databases with dates of coverage, contact with study authors to identify additional studies) in the search and date last searched. | | | **6** |
| Search | | 8 | Present full electronic search strategy for at least one database, including any limits used, such that it could be repeated. | | | **6** |
| Study selection | | 9 | State the process for selecting studies (i.e., screening, eligibility, included in systematic review, and, if applicable, included in the meta-analysis). | | | **6** |
| Data collection process | | 10 | Describe method of data extraction from reports (e.g., piloted forms, independently, in duplicate) and any processes for obtaining and confirming data from investigators. | | | **6,7** |
| Data items | | 11 | List and define all variables for which data were sought (e.g., PICOS, funding sources) and any assumptions and simplifications made. | | | **6,7** |
| Risk of bias in individual studies | | 12 | Describe methods used for assessing risk of bias of individual studies (including specification of whether this was done at the study or outcome level), and how this information is to be used in any data synthesis. | | | **8** |
| Summary measures | | 13 | State the principal summary measures (e.g., risk ratio, difference in means). | | | **8** |
| Synthesis of results | | 14 | Describe the methods of handling data and combining results of studies, if done, including measures of consistency (e.g., I^2^) for each meta-analysis. | | | **8** |
| **Section/topic** | | | **#** | **Checklist item** | **Reported on page #** |  |
| Risk of bias across studies | | | 15 | Specify any assessment of risk of bias that may affect the cumulative evidence (e.g., publication bias, selective reporting within studies). | 8 |  |
| Additional analyses | | | 16 | Describe methods of additional analyses (e.g., sensitivity or subgroup analyses, meta-regression), if done, indicating which were pre-specified. | 8 |  |
| **RESULTS** | | | | | 9-18 |  |
| Study selection | | | 17 | Give numbers of studies screened, assessed for eligibility, and included in the review, with reasons for exclusions at each stage, ideally with a flow diagram. | 9 |  |
| Study characteristics | | | 18 | For each study, present characteristics for which data were extracted (e.g., study size, PICOS, follow-up period) and provide the citations. | 10 |  |
| Risk of bias within studies | | | 19 | Present data on risk of bias of each study and, if available, any outcome level assessment (see item 12). | 10 |  |
| Results of individual studies | | | 20 | For all outcomes considered (benefits or harms), present, for each study: (a) simple summary data for each intervention group (b) effect estimates and confidence intervals, ideally with a forest plot. | 10 |  |
| Synthesis of results | | | 21 | Present results of each meta-analysis done, including confidence intervals and measures of consistency. | 10-18 |  |
| Risk of bias across studies | | | 22 | Present results of any assessment of risk of bias across studies (see Item 15). | 10-18 |  |
| Additional analysis | | | 23 | Give results of additional analyses, if done (e.g., sensitivity or subgroup analyses, meta-regression [see Item 16]). | 10-18 |  |
| **DISCUSSION** | | | | | 18-23 |  |
| Summary of evidence | | | 24 | Summarize the main findings including the strength of evidence for each main outcome; consider their relevance to key groups (e.g., healthcare providers, users, and policy makers). | 18-22 |  |
| Limitations | | | 25 | Discuss limitations at study and outcome level (e.g., risk of bias), and at review-level (e.g., incomplete retrieval of identified research, reporting bias). | 22 |  |
| Conclusions | | | 26 | Provide a general interpretation of the results in the context of other evidence, and implications for future research. | 23 |  |
| **FUNDING** | | | | |  |  |
| Funding | | | 27 | Describe sources of funding for the systematic review and other support (e.g., supply of data); role of funders for the systematic review. | 23 |  |

**Supplementary table 2: Searching History**

| **Name of database** | **Searching key or strategy** | **Total article found** | **Selection of article** |
| --- | --- | --- | --- |
| 1. **Pubmed** | (((((((((((((((((((((((((((((((((((((((((((((((((((((((((((((((((((((((((((population, risk) OR population at risk) OR factors attributed) OR predictors) OR insulin resistance) OR higher ir-homa) OR diet) OR visceral adipose tissue) OR metabolomics) OR intestinal microbiome) OR increased gut permeability) OR systemic inflammation) OR systemic inflammation) OR single nucleotide polymorphism) OR acute phase reaction) OR sitting time) OR risk factors) OR risk population)) OR sedentary life style) OR physical activity) OR metabolic syndrome) OR environmental influence) OR genetic influence) OR ethnicity) OR asia) OR rural) OR urban) OR third world phenotype) OR phosphatidylethanolamine n-methyltransferase) OR type 2 dm) OR type 1 dm) OR diabetes) OR dyslipidemia) OR cholesterol) OR triglyceride) OR low ldl) OR HDL) OR vldl) OR ALT) OR AST) OR ALP) OR sgpt) OR sgot) OR GGT) OR proinflammatory bacterial products) OR cytokines) OR faecalibacterium) OR ruminococcus species) OR lactobacillus) OR biological behavior) OR adipocyte size) OR hypertension) OR systolic blood pressure) OR diastolic blood pressure) OR gamma glutamyl transferase) OR gamma glutamyl transferase) OR lipodystrophy) OR familial hypobetalipoproteinemia,) OR lysosomal acid lipase deficiency) OR polycystic ovary syndrome) OR hypothyroidism) OR growth hormone deficiency) OR amiodarone) OR methotrexate) OR tamoxifen) OR jejunoileal bypass) OR starvation) OR total parenteral nutrition) OR dietary composition) OR fructose intake)) OR waist circumference)) AND ((((((lean) OR non obese) OR non-obese) OR low body mass index) OR lean body mass) OR lean body mass)) AND ((((((((NASH) OR NAFLD) OR Steatohepatitis) OR Fatty liver disease) OR Nonalcoholic Steatohepatitis) OR Nonalcoholic Steatohepatitides) OR Fatty liver) OR fatty liver disease) | **890** | **116** |
| **5.Bangaljol** | NAFLD | **14** | **2** |
| **6. Google scholar** | risk of nonalcoholic fatty liver disease in lean or nonobese factors OR predictors OR determinants OR NAFLD OR nonalcoholic -review -"hepatitis B" -"hepatitis C" -rat -PCOS -intervention -autopsy -childhood -paediatric | **239** | **11** |
| **7. Bibliographic search** |  |  | **32** |
| **Total** |  |  | **161** |

**Supplementary Table 3. Study type and quality assessment of the studies**

|  | **First author, year** | **Quality indicators from Newcastle-Ottawa Scale** | | | | | | | |  | **Study type** |
| --- | --- | --- | --- | --- | --- | --- | --- | --- | --- | --- | --- |
|  |  | **Selection of study groups** | | | | **Comparability of groups** | **Ascertain of exposure (outcome)** | | | **Score** |  |
| Number |  | **1** | **2** | **3** | **4** | **1** | **1** | **2** | **3** | **Total*** |  |
|  | Kim, 2004 | 1 | 1 |  | 1 | 2 | 2 | 1 |  | 8 | Cross-sectional |
|  | Das, 2010 | 1 | 1 | 1 | 1 | 2 | 1 | 1 |  | 8 | Case-control |
|  | Kwon, 2012 | 1 | 1 |  | 1 | 2 | 1 | 1 |  | 7 | Cross-sectional |
|  | Margariti, 2012 | 1 | 1 |  | 1 |  | 2 | 1 |  | 6 | Cross-sectional |
|  | Younossi, 2012 | 1 | 1 |  | 1 | 2 | 2 | 1 |  | 8 | Cross-sectional |
|  | Kumar, 2013 | 1 | 1 |  | 1 |  | 2 | 1 |  | 6 | Cross-sectional |
|  | Xu, 2013 | 1 | 1 |  |  | 1 | 2 | 1 |  | 6 | Cross-sectional |
|  | Alam, 2014 | 1 | 1 |  | 1 |  | 2 | 1 |  | 6 | Cross-sectional |
|  | Akyuz, 2015 | 1 | 1 |  |  | 2 | 1 | 1 |  | 6 | Cohort |
|  | Nishioji, 2015 | 1 | 1 |  | 1 | 2 | 2 | 1 |  | 8 | Cross-sectional |
|  | Wei, 2015 | 1 | 1 |  | 1 | 2 | 2 | 1 |  | 8 | Cross-sectional |
|  | Cho, 2016 | 1 | 1 |  | 1 | 2 | 1 | 1 |  | 8 | Cross-sectional |
|  | Kim, 2016 | 1 | 1 |  | 1 | 2 | 2 | 1 |  | 8 | Cross-sectional |
|  | Leung, 2016. | 1 | 1 |  |  | 2 | 1 | 1 | 1 | 7 | Cohort |
|  | Lu, 2016 | 1 | 1 |  | 1 | 2 | 2 | 1 |  | 8 | Cross-sectional |
|  | Wang, 2016 | 1 | 1 |  | 1 |  | 2 | 1 |  | 6 | Cross-sectional |
|  | Naderian, 2017 | 1 | 1 |  | 1 |  | 2 | 1 |  | 6 | Cross-sectional |
|  | Gonzalez-Cantero, 2018 | 1 | 1 |  | 2 | 1 | 2 | 1 |  | 8 | Cross-sectional |
|  | Kim, 2018 | 1 | 1 |  | 1 |  | 2 | 1 |  | 6 | Cross-sectional |
|  | Lee, 2018 | 1 | 1 |  | 1 | 1 | 1 | 1 |  | 7 | Cross-sectional |
|  | Shao, 2019 | 1 | 1 |  |  | 2 | 2 | 1 |  | 7 | Cross-sectional |
|  | Yun, 2019 | 1 | 1 |  | 1 |  | 2 | 1 |  | 6 | Cross-sectional |

Note: A study can be awarded a maximum of one star * for each numbered item within the Selection and Outcome categories. A maximum of two stars** can be given for comparability.

**Supplementary Table 4. Characteristics of studies included in the analysis**

|  | **Author, year. Country** | **Study design / Diagnosis of NAFLD** | **Definition of lean / non-lean** | **Sample size** | | **BMI**  **mean±SD** | | | |
| --- | --- | --- | --- | --- | --- | --- | --- | --- | --- |
|  |  |  |  | **NAFLD** | **Non-NAFLD** | **NAFLD** | | **Non-**  **NAFLD** | |
| **Serial** |  |  |  | **Lean / Non-lean** | **Lean/ Non-lean** | **Lean** | **Non-lean** | **Lean** | **Non-lean** |
|  | Kim, 2004  Korea | Population- based/  Hepatic steatosis assessed by liver ultrasound. | Lean BMI <25  Non-lean BMI ≥25 | 74/106 | 386/202 | 23.4±1.3 | 27.1±1.3 | 22.6±1.6 | 26.5±1.2 |
|  | Das,  2010  India | Community based study/  Hepatic steatosis assessed by liver ultrasound and CT scan | Lean BMI <25  Non-lean BMI >25 | 90/41 | 134/2 | 20.7±2.7 | NA | 19.5±2.7 | NA |
|  | Kwon,  2012  Korea | Hospital based/  Hepatic steatosis assessed by liver ultrasound | Lean BMI < 25  Non-lean BMI ≥ 25 | 3014/3025 | 20994/3011 | 23.1±1.3 | 27.4±2.1 | 21.3±2.0 | 26.7±1.6 |
|  | Margariti, 2012.  Greece. | Hospital-based/  Patients with hepatic steatosis assessed by liver ultrasound and/or liver histology | Lean BMI <25  Non-lean BMI ≥25 | 19/143 | NA | NA | NA | NA | NA |
|  | Younossi, 2012.  USA | Population- based/  Hepatic steatosis assessed by liver ultrasound. | Lean BMI <25  Non-lean BMI ≥25 | 431/2061 | 4026/5095 | 22.17±3.32 | 32.37±10.89 | 22.09±2.5 | 29.41±7.85 |
|  | Kumar, 2013.  India | Hospital-based/ Hepatic steatosis assessed by ultrasonography and liver biopsy. | Lean BMI <23  Non-lean BMI>25 | 27/141 | 131/NA | 21.3±1.9 | 28.3±3.2 | 22.0±0.76 | NA |
|  | Xu,  2013  China | Population-based/  NAFLD was diagnosed by ultrasonography. | Lean BMI  <25 | 502/NA | 6403/NA | 23.6 ±1.2 | NA | 21.5 ±2.0 | NA |
|  | Alam, 2014.  India | Hospital-based/ Patients with hepatic steatosis assessed by liver ultrasound | Lean BMI <25  Non-lean BMI≥25 | 119/346 | NA | 23±1.5 | 28.7±3.2 | NA | NA |
|  | Akyuz, 2015.  Turkey | Hospital-based/  Biopsy-proven NAFLD patients | Lean BMI <25  Non-lean BMI ≥25 | 37/446 | NA | 23.6±1.3 | 31.7±5.7 | NA | NA |
|  | Nishioji, 2015  Japan | Hospital based/  Hepatic steatosis assessed by liver ultrasound. | Lean BMI <25  Non-lean BMI ≥25 | 411/394 | 2285/181 | 23±1.5 (males)  22.6±1.5 (females) | 27.7±2.6 (males)  28.6±3.5 (females) | 21.3±2.0  (males)  20.3±2.1  (females) | 26.3±1.4  (males)  26.5±1.5  (females) |
|  | Wei, 2015.  Hong Kong | Population-based/  Liver fat assessed by proton-magnetic resonance spectroscopy | Lean BMI <25  Non-lean BMI ≥25 | 135/127 | NA | NA | NA | NA | NA |
|  | Cho, 2016.  South Korea | Population- based/  Hepatic steatosis assessed by liver ultrasound. | Lean BMI <25  Non-lean BMI>=25 | 213/347 | 1498/NA | 23.7±3.7 | 26.9±11.48 | 22.2±6.52 | NA |
|  | Kim, 2016  Seoul, Korea | Population- based/  Hepatic steatosis assessed by liver ultrasound. | Lean BMI <25  Non-lean BMI ≥25 | 136/211 | 1083/265 | 23.64±1.22 (males)  23.23±1.18 (females) | 27.04±1.58 (males)  27.79±2.35 (females) | 22.45±1.63  (males)  21.77±1.82  (females) | 26.8±1.71  (males)  26.79±1.46  (females) |
|  | Leung, 2016.  Hong Kong | Hospital-based /  Biopsy-proven NAFLD patients | Lean BMI <25  Non-lean BMI ≥25 | 72/235 | NA | 23.5±1.33 | 28.9±3.41 | NA | NA |
|  | Lu,  2016  China | Hospital-based/ hepatic steatosis assessed by ultrasonography | Lean BMI  ≤23 | 693/NA | 5223/NA | 21.57±1.45 | NA | 20.73±1.56 | NA |
|  | Wang,  2016  China | Hospital-based/  NAFLD diagnosed by liver ultrasound. | Lean BMI <25  Vs  Healthy control | 43/NA | 83/NA | 23.19 (22.19–24.22) | NA | 21.77 (20.7–23.38) | NA |
|  | Naderian, 2017  Iran | Population based/ Hepatic steatosis assessed by liver ultrasound | Lean BMI < 25 | 55/NA | 259/NA | Male:  23.28±1.24  Female:  23.14±1.67  Total:  23.21±  1.46 | NA | Male:  22.23±2.09  Female:  22.05±2.33  Total:  22.13±2.22 | NA |
|  | Gonzalez Cantero,2018  Spain | Hospital-based/  Hepatic steatosis assessed by liver ultrasound | Lean BMI 18.5-24.9  Non-lean BMI 25-29.9 | 25/30 | 30/28 | 23.46±1.96 | 28.00±1.46 | 22.59±1.61 | 27.06±1.41 |
|  | Kim,  2018  Korea | Hospital-based/ Biopsy-proven NAFLD patients | Lean BMI <25  Non-lean BMI ≥25 | 132/410 | 81/41 | 23.4 ± 1.3 | 29.2 ± 3.3 | 22.5 ± 1.7 | NA |
|  | Lee,  2018  China | Hospital based/ Hepatic steatosis assessed by ultrasonography | Lean BMI < 25  Non-lean BMI ≥25 | 208/285 | 745/770 | 23.8 ± 1.8 | 26.5 ± 2.3 | 21.4 ± 1.8 | 26.7 ± 2.2 |
|  | Shao,  2019  China | Hospital-based.  NAFLD is diagnosed by Ultrasound. | Lean BMI <23  Non-lean BMI ≥25 | 305/856 | NA | 21.6 ± 1.2 | 28.3 ± 2.7 | NA | NA |
|  | Yun,  2019  South Korea | Hospital-based .  NAFLD diagnosed by Ultrasonography | Lean BMI < 25  Non-lean BMI ≥ 25 | 27/49 | 168/24 | 22.8±2.6 | 27.3±1.6 | 21.8±1.8 | 26.2±1.1 |
|  | **Total sample size** | **69038** | - | **Lean**  **n=****6768**  **Non-lean n=****9253** | **Lean**  **n=****43398**  **Non-lean n=****9619** | - | - | - | - |

**Supplementary Table 5. Characteristics of relevant studies excluded from the analysis with main reasons for exclusion**

|  | **Author, year. Country** | **Study design / Diagnosis of NAFLD** | **Definition of lean / non-lean** | **Sample size** | | **BMI**  **mean±SD** | | | |  |
| --- | --- | --- | --- | --- | --- | --- | --- | --- | --- | --- |
|  |  |  |  | **NAFLD** | **Non-NAFLD** | **NAFLD** | | **Non-**  **NAFLD** | | **Main reason for exclusion** |
| **Serial** |  |  |  | **Lean / Non-lean** | **Lean/ Non-lean** | **Lean** | **Non-lean** | **Lean** | **Non-lean** |  |
|  | Alferink,  2019  Netherlands | Population- based.  Hepatic steatosis assessed by liver ultrasound | Lean BMI <25  Non-lean BMI ≥25 | 161/1462  67(male)+94(Female)/659(male)+803(female) | 432(male)+746(Female)/822(male)+986(female) | NA | NA | NA | NA | Baseline comparison of desired variables absent (including WC, SBP and DBP) |
|  | Bhat, 2013.  India | Hospital-based.  Patients with hepatic steatosis assessed by liver ultrasound | Lean BMI 18.6-22.9  Non-lean BMI > 23 | 30/120 | NA | 21.7±1.3 | 27.7±3.3 | NA | NA | BMI criteria do not match |
|  | Chen,  2006  Taiwan | Community based.  Hepatic steatosis assessed by liver ultrasound | Lean BMI <25  Non-lean BMI ≥25 | 24(female)+37(Male)/113(Female)+178(male) | 803(female)+580(Male)/368(Female)+286(male) | NA | NA | NA | NA | Baseline comparison of desired variables absent |
|  | Chen,  2019  Australia | Hospital-based.  Biopsy-proven NAFLD patients | Lean BMI <25  Non-lean BMI ≥25 | 99/439 | 30/NA | 23.2 ± 1.5 | 30.8 ± 4.7 | 22.8±1.9 | NA | WC, SBP, DBP absent |
|  | Eshraghian,  2019  Iran | Hospital based study.  Biopsy-proven NAFLD patients | Lean BMI < 25  Non-lean BMI ≥25 | 55/76 | 192/234 | 22.24±1.27 | 25.43±3.38 | 21.70±2.21 | 23.23±3.17 | WC, SBP and DBP absent |
|  | Feldman, 2016. Austria | Hospital-based. Patients with hepatic steatosis assessed by liver ultrasound | Lean BMI ≤ 25  Non-lean BMI ≥30 | 55/61 | 71/NA | 23.6±1.33 | 32.7±3.25 | 22.7±1.5 | NA | BMI criteria do not match |
|  | Feng, 2014.  China | Population-based.  Hepatic steatosis assessed by liver ultrasound. | Lean BMI <24  Non-lean BMI ≥24 | 134/764 | 597/284 | 22.74±1.13 | 27.57±2.63 | 21.37±1.71 | 25.98±1.66 | BMI criteria do not match |
|  | Honda,  2016  Japan | Hospital based study.  Biopsy-proven NAFLD patients | Lean BMI <25  Non-lean BMI ≥25 | 134/406 | 782/230 | 22.8 ± 1.5 | 29.8 ± 4.5 | 21.4 ± 2.1 | 27.0 ± 2.0 | WC, SBP and DBP absent |
|  | Kim,  2014  Korea | Hospital based study.  Hepatic steatosis assessed by liver ultrasound | Lean BMI <25  Non-lean BMI ≥25 | 199/151 | 955/170 | 23.2±1.4 | 26.5±1.3 | 21.8±1.9 | 26.4±1.2 | WC absent |
|  | Kimura,  2015  Japan | Hospital  based cross sectional study, Hepatic steatosis assessed by Liver Ultrasound | Lean BMI  18.5-22.9  Non-lean BMI ≥25 | NA | NA | NA | NA | NA | NA | WC absent |
|  | Li,  2019  China | Hospital based study, hepatic steatosis assessed by Liver Ultrasound | Lean BMI < 25  Non-lean BMI ≥ 25 | 101/ 395 | NA | NA | NA | NA | NA | Baseline comparison of desired variables absent |
|  | Nakamuta,  2008  Japan | Hospital Based Patients, by liver biopsy with histology | Lean BMI < 25  Non-lean BMI > 25 | 11/14 | NA | 23.2±1.5 | 30.6±4.0 | NA | NA | WC, SBP and DBP (quantitative values) absent |
|  | Niriella,  2018  Srilanka | Community based, prospective, cohort follow-up study ; Hepatic steatosis assessed by liver ultrasound on standard USS Criteria | Lean BMI < 23  Non-lean BMI ≥ 23 | 120/816 | NA | NA | NA | NA | NA | WC, SBP and DBP (quantitative values) absent |
|  | Oni,  2015  Brazil | Hospital based study, Hepatic steatosis assessed by liver ultrasound  And | Non-lean BMI ≥ 30  And >25 in who has WC more than normal | 775/1300 | NA | NA | NA | NA | NA | Unconventional BMI criteria.  Comparison of baseline data across BMI category not present. |
|  | Oniki,  2015  Japan | Hospital based both Cross sectional and Retrospective longitudinal studies, Hepatic steatosis assessed by Liver ultrasound | Lean BMI < 25  Non-lean BMI ≥ 25 | 55/64 | 402/70 | NA | NA | NA | NA | Age, WC, SBP and DBP (quantitative values) absent |
|  | Qi,  2015  China | Hospital based observational study, hepatic steatosis assessed by Liver Ultrasound | Lean BMI  ≤ 25 | 96/NA | 53/NA | 24.02 (22.89 – 24.77) | NA | 23.14 (21.56 – 24.17) | NA | Selective group of patients (OSA) and WC absent |
|  | Rotundo,  2018  USA | Population-based.  NAFLD is diagnosed by ultrasound. | Lean BMI <25  Non-lean BMI ≥25 |  |  | NA | NA | NA | NA | Comparison of baseline data across BMI category not present |
|  | Sun,  2016  United Kingdom | Hospital-based.  hepatic steatosis assessed by liver ultrasound. | Lean BMI < 25  Non-lean BMI ≥25 | 158400/NA | 25503/ NA | 23.37±1.24 | NA | 21.12±2.07 | NA | WC absent |
|  | Tobari,  2019  Japan | Hospital-based. biopsy-proven steatosis or steatohepatitis. | Lean BMI < 25  Non-lean BMI ≥ 25 – 30  Severely obese>30 | 256/305 | NA | Male: 22.8 (±1.7),  Female: 22.7 (±1.8) | Male: 27.2 (±1.4), Female: 27.3±1.4 | NA | NA | Non-lean patients were divided into two groups and total effect measures for non-lean patients absent. |
|  | Xu,  2016  China | Hospital-based.  NAFLD diagnosed by liver ultrasound. | Lean BMI  Non-lean | NA/NA | NA/NA | NA | NA | NA | NA | No definition of lean/non-lean. |
|  | Yang,  2017  China | Hospital-based.  NAFLD diagnosed by Ultrasonography | Non-lean BMI ≥25 |  | NA | NA | NA | NA | NA | WC, SBP, DBP absent |
|  | Yang,  2016  Korea | NAFLD diagnosed by ultrasonography. | Lean BMI <23  Non-lean BMI>=23 | 943/1312 | 4426/1646 | NA | NA | NA | NA | Comparison of baseline characteristics of lean patients absent |
|  | Yasutak,  2009  Japan | Hospital-based.  NAFLD diagnosed by Ultrasonography, CT scan and biopsy But not defined who many number was diagnosed by each methods. | Lean BMI  Non-lean BMI  BMI cut of 25  But not defined clearly | 12/44 | NA | NA | NA | NA | NA | No obvious demarcation of lean and non-lean |
|  | Zhang,  2017  China | Hospital-based.  NAFLD diagnosed by Ultrasonography  (age >20 years) | Lean BMI <25 | 5179/NA | 1630/NA | 23.7 (22.7–24.4) | NA | 21.7 (20.1–23.1 | NA | WC absent |
|  | Zheng,  2017  China | Population-based.  NAFLD diagnosed by Ultrasonography | Lean BMI ≤25 | 7503/NA | 84421/NA | Mild – 23.94±0.91  Moderate – 24.08±0.81 | NA | 23.43 ± 1.27 | NA | BMI does not match |

NA: data not available

| **Factors** | **No of Studies** | **Pooled Mean Difference (95% CI)** | **Test for overall effect** | | **Test for heterogeneity** | | | |
| --- | --- | --- | --- | --- | --- | --- | --- | --- |
|  |  |  | **z- value** | **p-value** | **Q statistic** | **p-value** | $\boldsymbol{\tau}^{\boldsymbol{2}}$ | $\boldsymbol{I}^{\boldsymbol{2}}$ |
| **Age** | 17 | 2.87 [1.74- 4.00] | 4.98 | 0.00 | 350.08 | 0.00 | 4.10 | 95.4% |
| **WC** | 16 | 5.39[4.58; 6.20] | 13.07 | 0.00 | 234.65 | 0.00 | 2.11 | 93.6% |
| **BMI** | 17 | 1.40[0.63; 2.18] | 3.54 | 0.00 | 11021.79 | 0.00 | 2.60 | 99.9% |
| **SBP** | 13 | 5.19[4.23; 6.15] | 10.55 | 0.00 | 59154.74 | 0.00 | 1.81 | 100% |
| **DBP** | 13 | 2.92[2.43; 3.42] | 11.61 | 0.00 | 665.94 | 0.00 | 0.30 | 98.2% |
| **FBS** | 17 | 5.17[4.18; 6.16] | 10.19 | 0.00 | 2923.55 | 0.00 | 2.10 | 99.5% |
| **HbA1c** | 4 | 0.29 [0.11; 0.48] | 3.11 | 0.00 | 28.90 | 0.00 | 0.03 | 89.6% |
| **HOMA-IR** | 11 | 0.49[0.29; 0.68] | 4.82 | 0.00 | 4738.93 | 0.00 | 0.08 | 99.8% |
| **TC** | 15 | 10.32[6.68; 13.96] | 5.55 | 0.00 | 58.17 | 0.00 | 32.29 | 75.9% |
| **HDL** | 16 | -5.74[-7.06; -4.43] | -8.55 | 0.00 | 8868.76 | 0.00 | 5.35 | 99.8% |
| **LDL** | 13 | 9.56 [6.07; 13.04] | 5.38 | 0.00 | 76.15 | 0.00 | 28.52 | 84.2% |
| **TG** | 17 | 41.46[39.02; 43.89] | 33.36 | 0.00 | 582.61 | 0.00 | 6.35 | 97.3% |
| **UA** | 7 | 0.66[0.48; 0.84] | 7.25 | 0.00 | 43.20 | 0.00 | 0.05 | 86.1% |
| **ALT** | 15 | 8.12[6.21; 10.03] | 8.33 | 0.00 | 523.65 | 0.00 | 11.30 | 97.3% |
| **AST** | 11 | 2.72[1.91; 3.53] | 6.61 | 0.00 | 99.76 | 0.00 | 1.06 | 90.0% |
| **GGT** | 12 | 11.21[9.02; 13.40] | 10.02 | 0.00 | 57.47 | 0.00 | 8.46 | 80.9% |

**Supplementary Table 6: Lean non-NAFLD vs NAFLD- Summary of overall effect and test for heterogeneity for all variables**

**Supplementary Table 7:** Lean non-NAFLD vs NAFLD- Summary of subgroup analysis across type of study population

| **Factors** | **type** | **Pooled Mean Difference (95% CI)** | **Q- Statistics** | **P-value** | $\boldsymbol{\tau}^{\boldsymbol{2}}$ | $\boldsymbol{I}^{\boldsymbol{2}}$ |
| --- | --- | --- | --- | --- | --- | --- |
| Age | Population based | 1.99[0.74; 3.25] | 49.79 | 0.00 | 2.19 | 85.9% |
|  | Hospital based | 3.86[2.29; 5.43] | 73.70 | 0.00 | 3.95 | 89.1% |
|  | Overall | 2.87[1.74; 4.00] | 350.08 | 0.00 | 4.10 | 95.4% |
| BMI | Population based | 1.21 [0.33; 2.09] | 1337.11 | 0.00 | 1.57 | 1337.11 |
|  | Hospital based | 1.59 [0.87; 2.31] | 1021.73 | 0.00 | 1.15 | 99.2% |
|  | Overall | 1.40 [0.63; 2.18] | 11021.79 | 0.00 | 2.60 | 99.9% |
| WC | Population based | 5.05 [3.30; 6.80] | 124.39 | 0.00 | 4.95 | 95.2% |
|  | Hospital based | 5.48 [4.53; 6.43] | 93.05 | 0.00 | 1.55 | 91.4% |
|  | Overall | 5.39[4.58; 6.20] | 234.65 | 0.00 | 2.110 | 93.6% |
| SBP | Population based | 5.73 [3.45; 7.99] | 22.02 | 0.001 | 4.96 | 77.3% |
|  | Hospital based | 4.81 [3.61; 6.00] | 59051.02 | 0.00 | 1.81 | 100.0% |
|  | Overall | 5.19[4.23; 6.15] | 59154.74 | 0.00 | 1.8099 | 100% |
| DBP | Population based | 3.81 [2.18; 5.44] | 13.60 | 0.02 | 2.36 | 63.2% |
|  | Hospital based | 2.31 [1.78; 2.85] | 552.28 | 0.00 | 0.25 | 98.9% |
|  | Overall | 2.92 [2.43; 3.42] | 665.94 | 0.00 | 0.30 | 98.2% |
| FBS | Population based | 4.33 [3.22; 5.44] | 8.18 | 0.23 | 0.52 | 26.6% |
|  | Hospital based | 5.45 [4.21; 6.69] | 2914.99 | 0.00 | 2.11 | 99.7% |
|  | Overall | 5.17[4.18; 6.16] | 2923.55 | 0.00 | 2.10 | 99.5% |
| HOMA-IR | Population based | 0.29 [-0.23; 0.82] | 267.85 | 0.00 | 0.41 | 98.1% |
|  | Hospital based | 0.68 [ 0.40; 0.96] | 3177.54 | 0.00 | 0.06 | 99.9% |
|  | Overall | 0.49[0.29; 0.68] | 4738.93 | 0.00 | 0.085 | 99.8% |
| TC | Population based | 12.39 [5.63; 19.15] | 34.80 | 0.00 | 62.26 | 82.8% |
|  | Hospital based | 8.54 [6.52; 10.56] | 2.35 | 0.98 | 0.0 | 0.0% |
|  | Overall | 10.32[6.68; 13.96] | 58.17 | 0.00 | 32.29 | 75.9% |
| HDL | Population based | -3.98 [-6.33; -1.64] | 56.23 | 0.00 | 8.48 | 89.3% |
|  | Hospital based | -7.21 [-9.00; -5.41] | 8811.91 | 0.000 | 5.36 | 99.9% |
|  | Overall | -5.74[-7.06; -4.43] | 8868.76 | 0.00 | 5.35 | 99.8% |
| LDL | Population based | 12.61 [8.78; 16.44] | 11.39 | 0.04 | 11.52 | 56.1% |
|  | Hospital based | 7.17 [3.64; 10.70] | 22.72 | 0.001 | 12.97 | 73.6% |
|  | Overall | 9.56 [6.07; 13.04] | 76.15 | 0.00 | 28.52 | 84.2% |
| TG | Population based | 48.55 [25.66; 71.44] | 72.52 | 0.00 | 830.32 | 91.7% |
|  | Hospital based | 36.22 [34.00; 38.44] | 352.80 | 0.00 | 3.86 | 97.4% |
|  | Overall | 41.46[39.02; 43.89] | 582.61 | 0.00 | 6.349 | 97.3% |
| UA | Population based | 0.83 [0.64; 1.02] | 5.97 | 0.05 | 0.02 | 66.5% |
|  | Hospital based | 0.53 [0.27; 0.78] | 24.09 | 0.00 | 0.05 | 87.5% |
|  | Overall | 0.66[0.48; 0.84] | 43.20 | 0.00 | 0.045 | 86.1% |
| ALT | Population based | 5.50 [3.62; 7.37] | 40.10 | 0.00 | 4.08 | 87.5% |
|  | Hospital based | 9.79 [7.39; 12.18] | 119.79 | 0.00 | 9.71 | 93.3% |
|  | Overall | 8.12 [6.21; 10.03] | 523.65 | 0.00 | 11.30 | 97.3% |
| AST | Population based | 2.30[1.02; 3.58] | 10.46 | 0.02 | 1.14 | 71.3% |
|  | Hospital based | 3.23 [1.89; 4.57] | 31.34 | 0.00 | 2.07 | 80.9% |
|  | Overall | 2.72 [1.91; 3.53] | 99.76 | 0.00 | 1.06 | 90.0% |
| GGT | Population based | 12.18 [7.73; 16.63] | 17.35 | 0.001 | 16.05 | 82.7% |
|  | Hospital based | 10.27 [8.24; 12.31] | 17.30 | 0.016 | 3.26 | 59.5% |
|  | Overall | 11.21[9.02; 13.40] | 57.47 | 0.00 | 8.46 | 80.9% |

**Supplementary Table 8:** Lean non-NAFLD vs NAFLD- Summary of subgroup analysis across ethnicity

| **Factors** | **type** | **Pooled Mean Difference (95% CI)** | **Q- Statistics** | **P-value** | $\boldsymbol{\tau}^{\boldsymbol{2}}$ | $\boldsymbol{I}^{\boldsymbol{2}}$ |
| --- | --- | --- | --- | --- | --- | --- |
| Age | Korean | 3.04[ 0.77; 5.31] | 99.96 | 0.00 | 7.95 | 94.0% |
|  | Caucasian | 1.03[-1.69; 3.77] | 9.25 | 0.026 | 4.78 | 67.6% |
|  | Japanese | 3.45[-3.50; 10.41] | 41.51 | 0.00 | 24.60 | 97.6% |
|  | Chinese | 3.64[1.58; 5.70] | 20.06 | 0.00 | 3.14 | 85.0% |
|  | Overall | 2.87[1.74; 4.00] | 350.08 | 0.00 | 4.10 | 95.4% |
| BMI | Korean | 1.43 [0.58; 2.29] | 350.21 | 0.00 | 1.27 | 98.3% |
|  | Caucasian | 0.76[0.04; 1.49] | 29.12 | 0.00 | 0.45 | 89.7% |
|  | Japanese | 1.20 [1.41; 2.58] | 11.49 | 0.001 | 0.16 | 91.3% |
|  | Chinese | 1.65 [0.82; 2.49] | 295.83 | 0.00 | 0.70 | 99.0% |
|  | Overall | 1.40 [0.63; 2.18] | 11021.79 | 0.00 | 2.60 | 99.9% |
| SBP | Korean | 3.37 [2.04; 4.70] | 58971.62 | 0.000 | 1.80 | 100.0% |
|  | Caucasian | 8.56 [2.96; 14.16] | 0.00 | -- | -- | -- |
|  | Japanese | 6.28 [3.05; 9.52] | 3.77 | 0.052 | 4.00 | 73.4% |
|  | Chinese | 7.57 [6.47; 8.66] | 3.28 | 0.194 | 0.36 | 39.1% |
|  | Overall | 5.19[4.23; 6.15] | 59154.74 | 0.00 | 1.81 | 100% |
| DBP | Korean | 1.99 [1.36; 2.61] | 89.2 | 0.00 | 0.25 | 98.9% |
|  | Caucasian | 3.31 [0.46; 6.16] | 0.00 | -- | - | -- |
|  | Japanese | 4.23 [3.28; 5.17] | 0.09 | 0.789 | 0.0 | 0.0% |
|  | Chinese | 3.56 [0.27; 6.84] | 47.69 | 0.00 | 7.48 | 95.8% |
|  | Overall | 2.92 [2.43; 3.42] | 665.94 | 0.00 | 0.30 | 98.2% |
| FBS | Korean | 4.36 [2.96; 5.77] | 2865.93 | 0.00 | 2.09 | 99.8% |
|  | Caucasian | 5.34 [1.52; 9.17] | 0.97 | 0.616 | 0 | 0.0% |
|  | Japanese | 8.38 [3.68; 13.08] | 7.80 | 0.005 | 10.04 | 87.2% |
|  | Chinese | 5.28 [3.03; 7.53] | 22.73 | 0.00 | 3.657 | 86.8% |
|  | Overall | 5.17[4.18; 6.16] | 2923.55 | 0.00 | 2.10 | 99.5% |
| HbA1c | Korean | 0.34 [-0.25; 0.93] | 12.64 | 0.002 | 0.17 | 92.1% |
|  | Japanese | 0.30 [ 0.10; 0.50] | 9.29 | 0.00 | 0.02 | 89.2% |
|  | Overall | 0.29 [0.11; 0.48] | 28.90 | 0.00 | 0.03 | 89.6% |
| HOMA-IR | Korean | 0.38 [0.18; 0.58] | 3242.40 | 0.00 | 0.07 | 99.8% |
|  | Caucasian | 1.01 [0.21; 1.82] | 25.09 | 0.00 | 0.39 | 92.0% |
|  | Overall | 0.49[0.29; 0.68] | 4738.93 | 0.00 | 0.085 | 99.8% |
| TC | Korean | 12.20 [ 8.44; 15.96] | 5.85 | 0.321 | 3.34 | 14.6% |
|  | Caucasian | 3.47 [-23.07; 30.01] | 18.36 | 0.00 | 486.64 | 89.1% |
|  | Japanese | 7.054 [ 3.55; 10.56] | 0.13 | 0.720 | 0 | 0.0% |
|  | Chinese | 11.97 [ 5.25; 18.68] | 23.81 | 0.00 | 37.59 | 87.4% |
|  | Overall | 10.32[6.68; 13.96] | 58.17 | 0.00 | 32.29 | 75.9% |
| LDL | Korean | 9.95 [ 5.90; 14.01] | 7.84 | 0.165 | 9.06 | 36.2% |
|  | Caucasian | 9.24 [-5.52; 23.99] | 2.58 | 0.108 | 71.56 | 61.3% |
|  | Japanese | 10.86 [ 7.66; 14.07] | 0.18 | 0.675 | 0.0 | 0.0% |
|  | Chinese | 8.36 [ 0.49; 16.23] | 60.15 | 0.000 | 46.72 | 96.7% |
|  | Overall | 9.56 [6.07; 13.04] | 76.15 | 0.00 | 28.52 | 84.2% |
| HDL | Korean | -5.99 [ -7.81; -4.18] | 8752.86 | 0.00 | 5.34 | 99.9% |
|  | Caucasian | 0.26 [ -2.33; 2.85] | 2.62 | 0.270 | 1.32 | 23.6% |
|  | Japanese | -10.71 [-13.64; -7.77] | 3.83 | 0.050 | 3.32 | 73.9% |
|  | Chinese | -5.33 [ -7.98; -2.68] | 20.77 | 0.00 | 4.29 | 90.4% |
|  | Overall | -5.74[-7.06; -4.43] | 8868.76 | 0.00 | 5.35 | 99.8% |
| TG | Korean | 32.37 [30.08; 34.66] | 265.89 | 0.00 | 2.92 | 97.4% |
|  | Caucasian | 23.40 [ 8.67; 38.12] | 3.55 | 0.169 | 74.55 | 43.7% |
|  | Japanese | 46.36 [40.19; 52.53] | 0.14 | 0.703 | 0 | 0.0% |
|  | Chinese | 65.40 [41.49; 89.30] | 102.46 | 0.000 | 549.95 | 97.1% |
|  | Overall | 41.46[39.02; 43.89] | 582.61 | 0.00 | 6.349 | 97.3% |
| UA | Korean | 0.72[0.57; 0.87] | 0.26 | 0.611 | 0 | 0.0% |
|  | Japanese | 0.60 [0.21; 1.00] | 11.31 | 0.001 | 0.07 | 91.2% |
|  | Chinese | 0.62 [0.29; 0.95] | 23.97 | 0.000 | 0.07 | 91.7% |
|  | Overall | 0.66[0.48; 0.84] | 43.20 | 0.00 | 0.05 | 86.1% |
| ALT | Korean | 8.45 [5.66; 11.23] | 74.61 | 0.00 | 11.30 | 92.0% |
|  | Caucasian | 3.58 [0.93; 6.23] | 7.93 | 0.019 | 3.28 | 74.8% |
|  | Japanese | 7.85 [6.26; 9.44] | 0.00 | 0.951 | 0 | 0.0% |
|  | Chinese | 11.05 [3.89; 18.21] | 39.58 | 0.00 | 37.35 | 94.9% |
|  | Overall | 8.12 [6.21; 10.03] | 523.65 | 0.00 | 11.30 | 97.3% |
| AST | Korean | 3.45 [1.86; 5.05] | 17.96 | 0.001 | 2.27 | 77.7% |
|  | Caucasian | 3.09 [0.14; 6.05] | 13.91 | 0.001 | 4.71 | 85.6% |
|  | Japanese | 2.54 [0.89; 4.20] | 2.83 | 0.093 | 0.93 | 64.6% |
|  | Chinese | 1.91 [0.09; 3.73] | 0.0 | -- | - | - |
|  | Overall | 2.72 [1.91; 3.53] | 99.76 | 0.00 | 1.06 | 90.0% |
| GGT | Korean | 11.00 [8.87; 13.13] | 7.46 | 0.189 | 2.20 | 33.0% |
|  | Caucasian | 21.52 [3.58; 39.46] | 0.00 | -- | - | -- |
|  | Japanese | 7.96 [5.03; 10.90] | 0.16 | 0.689 | 0 | 0.0% |
|  | Chinese | 14.32 [8.44; 20.21] | 40.82 | 0.000 | 22.05 | 95.1% |
|  | Overall | 11.21[9.02; 13.40] | 57.47 | 0.001 | 8.46 | 80.9% |

**Supplementary Table 9: Lean non-NAFLD vs NAFLD- Summary of subgroup analysis across Eastern and other studies.**

| Factors | type | Pooled Mean Difference (95% CI) | Q- Statistics | P-value | $\boldsymbol{\tau}^{\boldsymbol{2}}$ | $\boldsymbol{I}^{\boldsymbol{2}}$ |
| --- | --- | --- | --- | --- | --- | --- |
| Age | Eastern studies | 3.30[1.91; 4.69] | 164.52 | 0.00 | 2.30 | 92.7% |
|  | Other studies | 1.03[-1.69; 3.76] | 9.25 | 0.46 | 2.19 | 67.6% |
|  | Overall | 2.87[1.74; 4.00] | 350.08 | 0.00 | 4.10 | 95.4% |
| BMI | Eastern studies | 1.59[1.09; 2.10] | 1183.19 | 0.00 | 0.83 | 99.0% |
|  | Other studies | 0.76[0.04; 1.49] | 29.12 | 0.04 | 0.45 | 89.7% |
|  | Overall | 1.40 [0.63; 2.18] | 11021.79 | 0.00 | 2.60 | 99.9% |
| WC | Eastern studies | 5.67[4.84; 6.50] | 211.18 | 0.00 | 1.95 | 94.3% |
|  | Other studies | 3.54[0.05; 7.03] | 7.68 | 0.05 | 6.88 | 73.9% |
|  | Overall | 5.39[4.58; 6.20] | 234.65 | 0.00 | 2.11 | 93.6% |
| FBS | Eastern studies | 5.16[4.13; 6.18] | 2922.38 | 0.00 | 2.10 | 99.6% |
|  | Other studies | 5.34[1.52; 9.17] | 0.97 | 0.01 | 0.00 | 0 |
|  | Overall | 5.17[4.18; 6.16] | 2923.55 | 0.00 | 2.10 | 99.5% |
| HOMA-IR | Eastern studies | 0.38[0.18; 0.58] | 3242.40 | 0.00 | 0.07 | 99.8% |
|  | Other studies | 1.01[0.21; 1.82] | 25.9 | 0.01 | 0.39 | 92% |
|  | Overall | 0.49[0.29; 0.68] | 4738.93 | 0.00 | 0.09 | 99.8% |
| SBP | Eastern studies | 5.10[4.13; 6.08] | 59150.00 | 0.00 | 1.81 | 100% |
|  | Other studies | 8.56[2.96; 14.16] | 0.00 | 0.00 | -- | -- |
|  | Overall | 5.19[4.23; 6.15] | 59154.74 | 0.00 | 1.81 | 100% |
| DBP | Eastern studies | 2.91[2.41; 3.41] | 664.73 | 0.00 | 0.30 | 98.3% |
|  | Other studies | 3.31[0.46; 6.16] | 0.00 | 0.02 | -- | -- |
|  | Overall | 2.92 [2.43; 3.42] | 665.94 | 0.00 | 0.30 | 98.2% |
| TC | Eastern studies | 11.03[7.69; 14.36] | 38.65 | 0.00 | 21.17 | 71.5% |
|  | Other studies | 3.47[-23.07; 30.01] | 18.65 | 0.80 | 486.64 | 89.1% |
|  | Overall | 10.32[6.68; 13.96] | 58.17 | 0.00 | 32.29 | 75.9% |
| LDL | Eastern studies | 9.51[ 5.84; 13.19] | 73.16 | 0.00 | 28.91 | 86.3% |
|  | Other studies | 9.24[-5.52; 23.99] | 2.58 | 0.22 | 71.56 | 61.3% |
|  | Overall | 9.56 [6.07; 13.04] | 76.15 | 0.00 | 28.52 | 84.2% |
| HDL | Eastern studies | -6.62[-8.04; -5.21] | 8847.28 | 0.00 | 5.34 | 99.9% |
|  | Other studies | 0.26[-2.33; 2.85] | 2.62 | 0.84 | 1.32 | 23.6% |
|  | Overall | -5.74[-7.06; -4.43] | 8868.76 | 0.00 | 5.35 | 99.8% |
| TG | Eastern studies | 42.35[39.86; 44.84] | 576.55 | 0.00 | 6.32 | 97.7% |
|  | Other studies | 23.40 [ 8.67; 38.12] | 3.55 | 0.00 | 74.55 | 43.7% |
|  | Overall | 41.46[39.02; 43.89] | 582.61 | 0.00 | 6.35 | 97.3% |
| ALT | Eastern studies | 8.82[6.90; 10.74] | 155.03 | 0.00 | 9.10 | 92.9% |
|  | Other studies | 3.58[0.93; 6.23] | 7.93 | 0.01 | 3.27 | 74.8% |
|  | Overall | 8.12 [6.21; 10.03] | 523.65 | 0.00 | 11.30 | 97.3% |
| AST | Eastern studies | 2.94[1.96; 3.92] | 26.04 | 0.00 | 1.22 | 73.1% |
|  | Other studies | 3.09[0.14; 6.05] | 13.91 | 0.04 | 4.71 | 85.6% |
|  | Overall | 2.72 [1.91; 3.53] | 99.76 | 0.00 | 1.06 | 90.0% |
| GGT | Eastern studies | 11.07[8.86; 13.27] | 56.34 | 0.00 | 8.46 | 82.2% |
|  | Other studies | 21.52[3.58; 39.46] | 0.00 | 0.02 | -- | -- |
|  | Overall | 11.21[9.02; 13.40] | 57.47 | 0.00 | 8.46 | 80.9% |


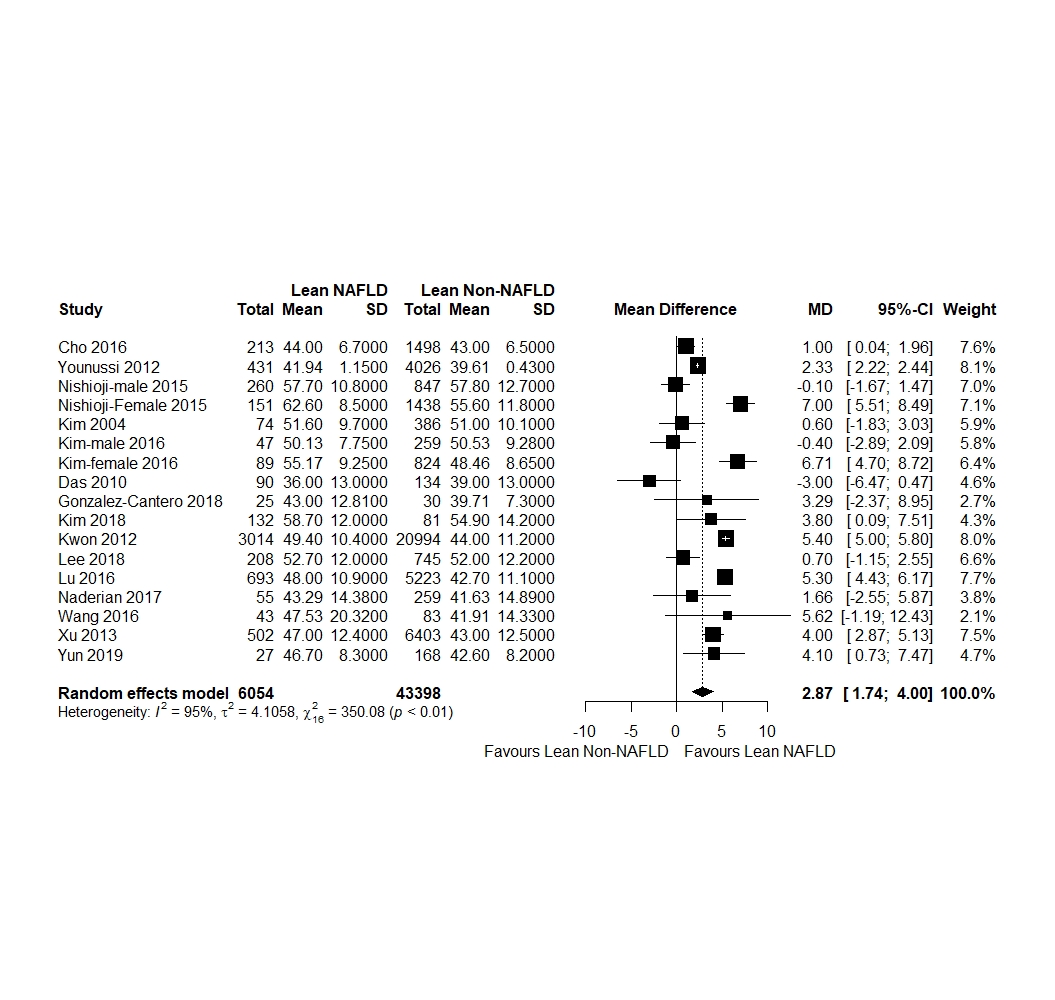


Figure 2. Forest plot for age.


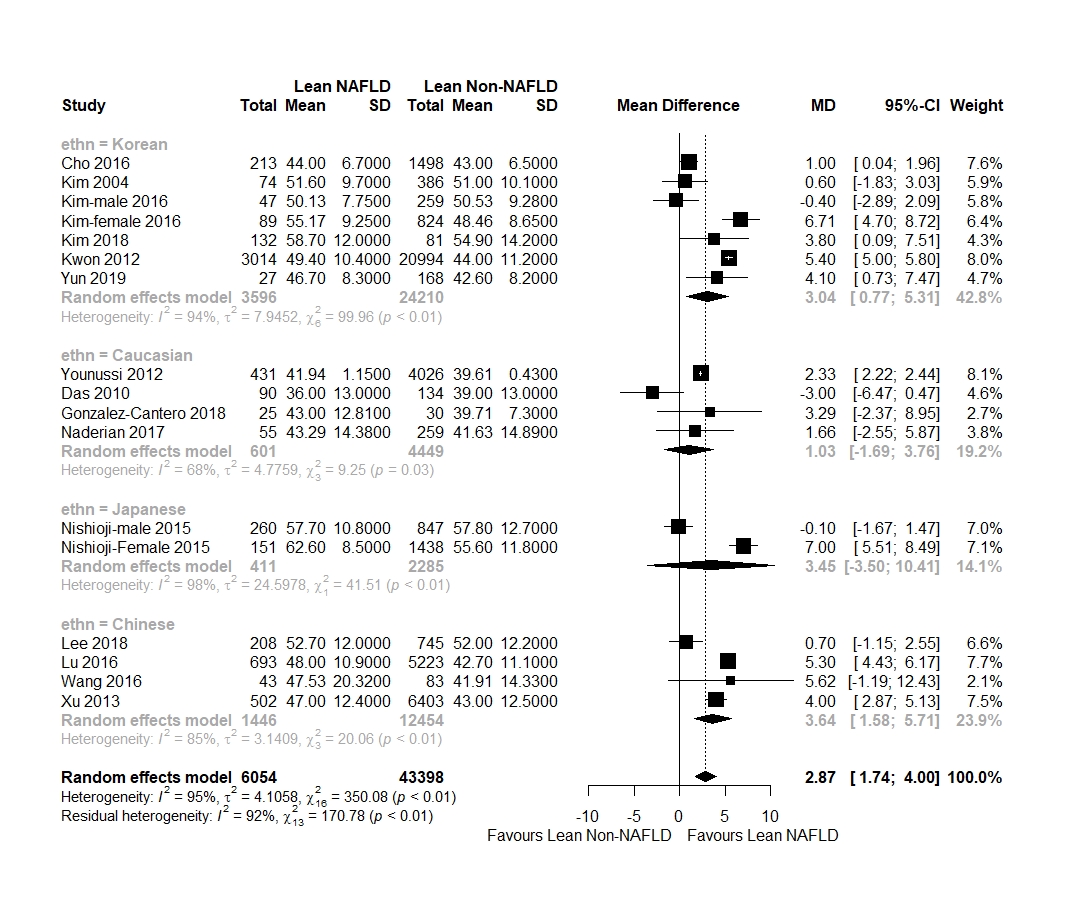


**Supplementary Figure 2A. Forest plot for age- Subgroup Analysis by ethnicity**


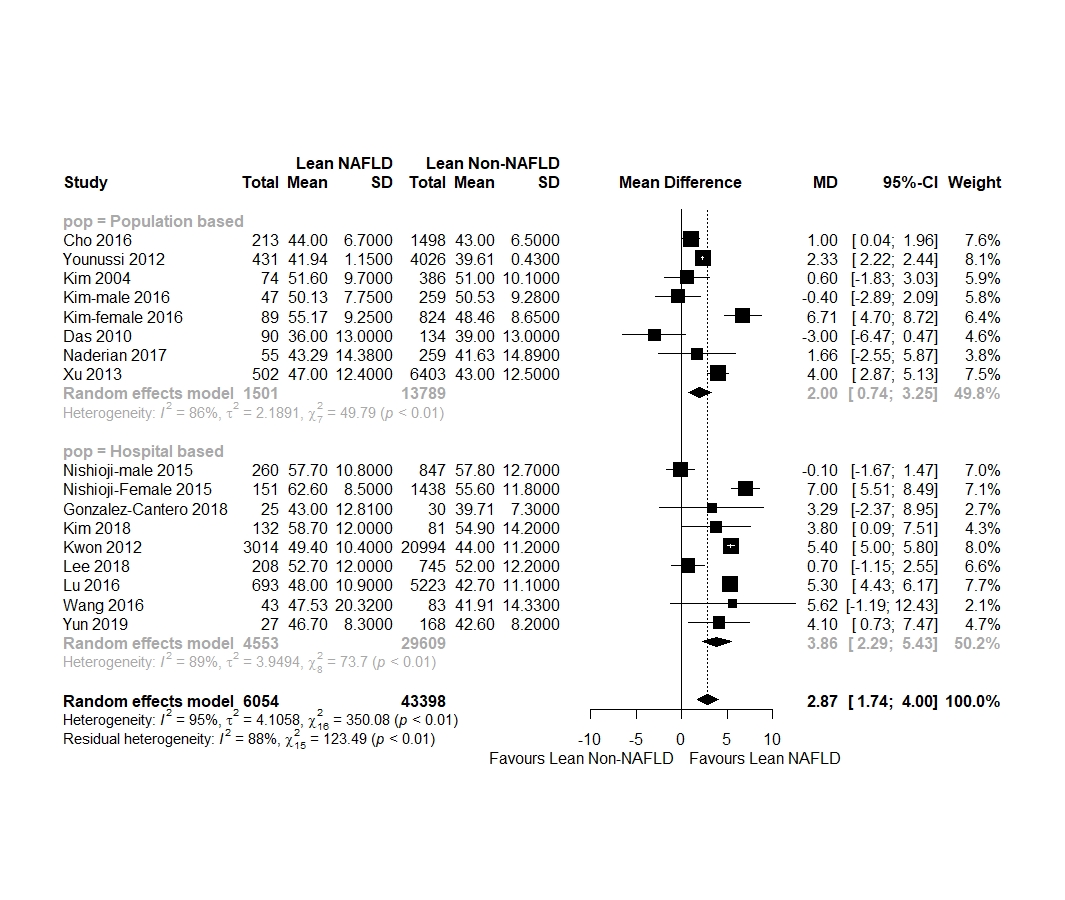


**Supplementary Figure 2B: Forest plot for age- Subgroup analysis by study population**


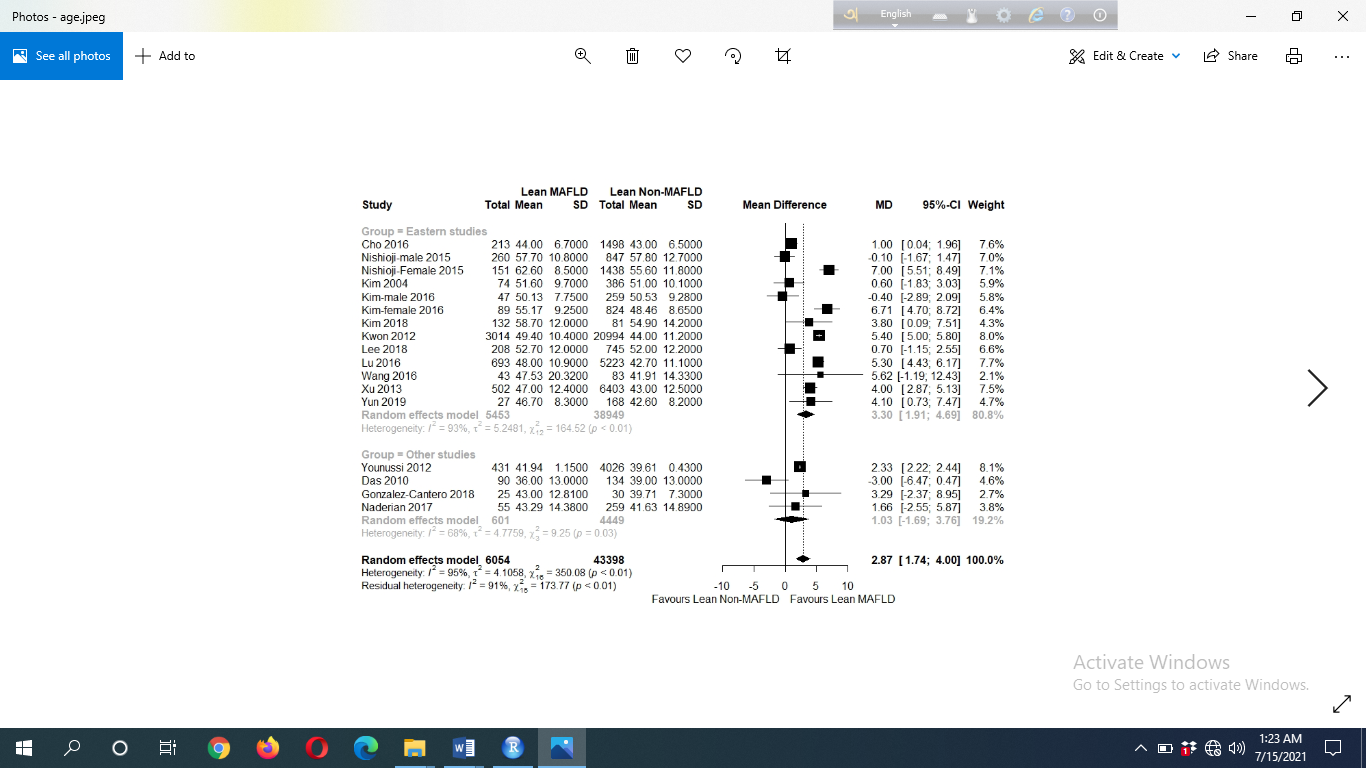


**Supplementary Figure 2C. Forest plot for age- Subgroup Analysis by Eastern and other studies**


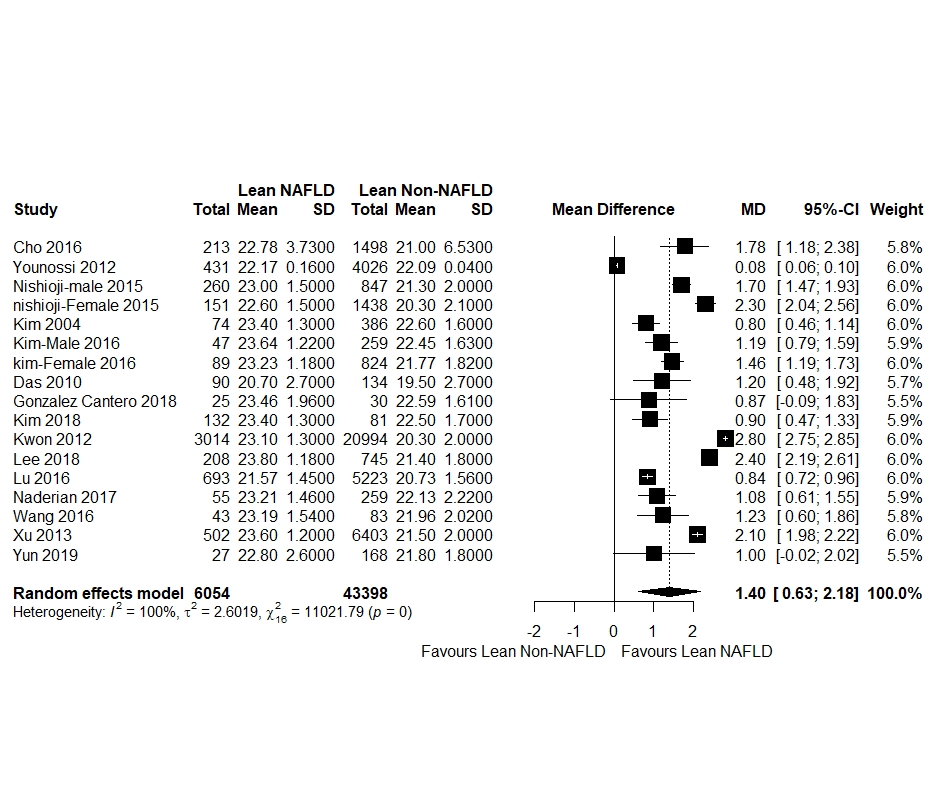


**Figure 3. Forest plot for BMI**


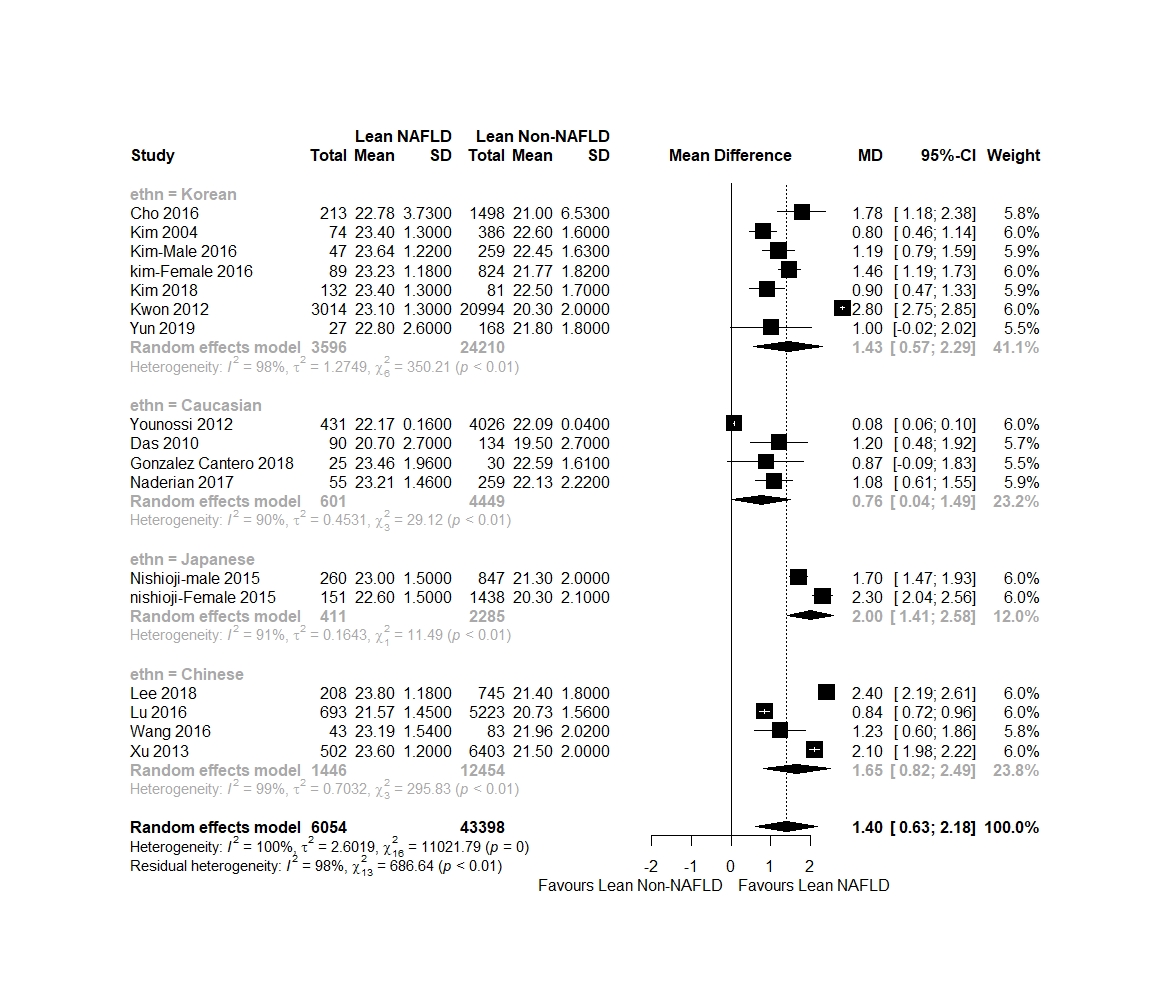


**Supplementary Figure 3A. Forest plot for BMI - subgroup analysis by ethnicity**


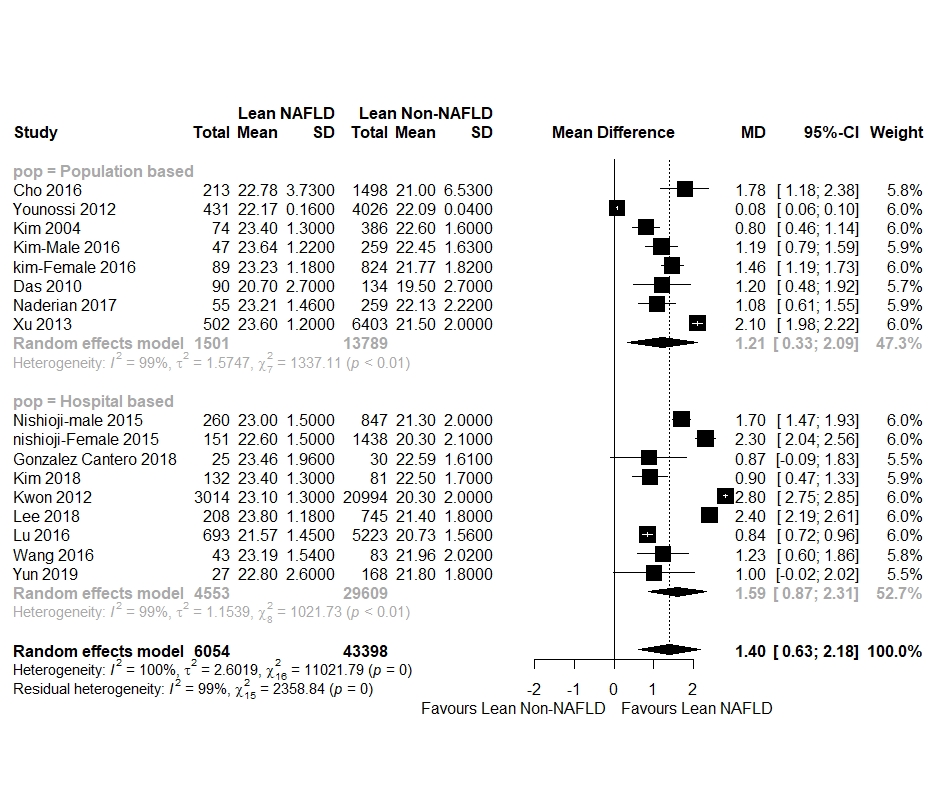


**Supplementary Figure 3B. Forest plot for BMI- subgroup analysis by study population**


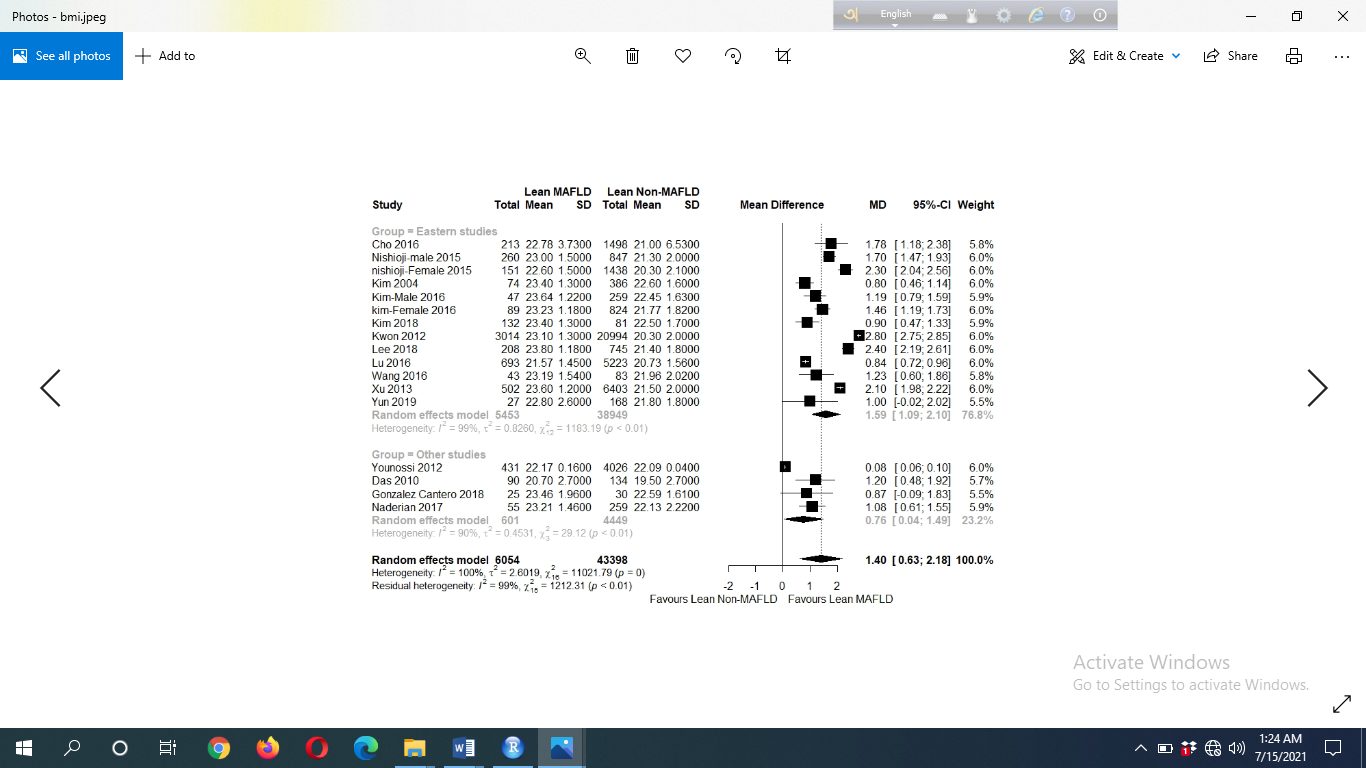


**Supplementary Figure 3C. Forest plot analysis for BMI- Subgroup analysis by Eastern and other studies**


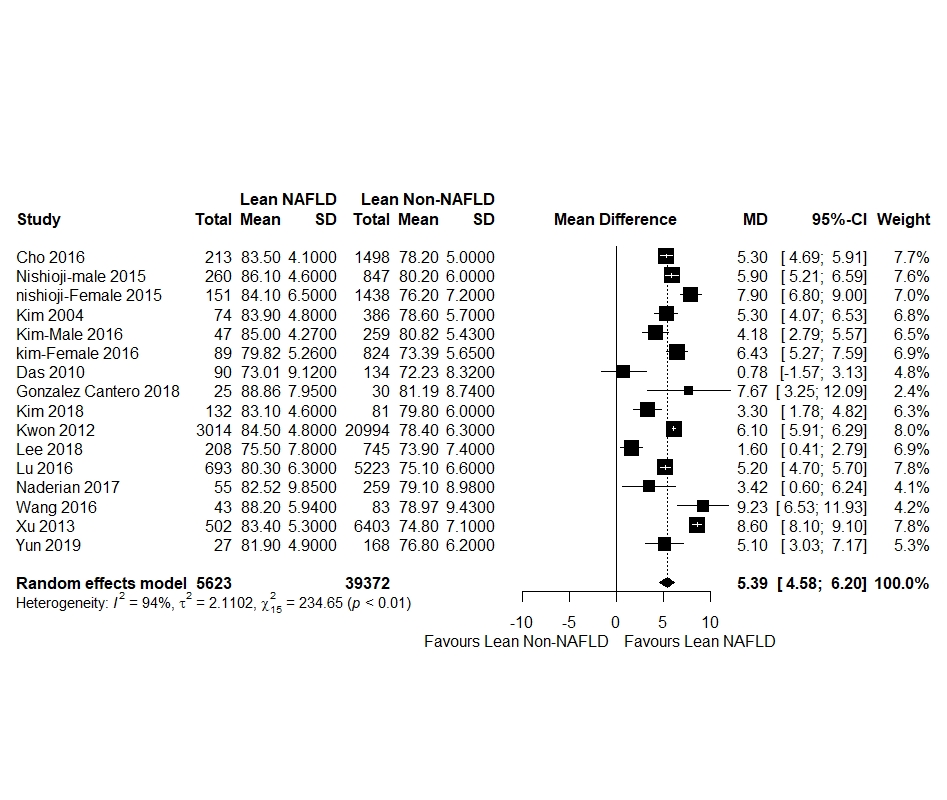


**Figure 4. Forest plot for waist circumference**


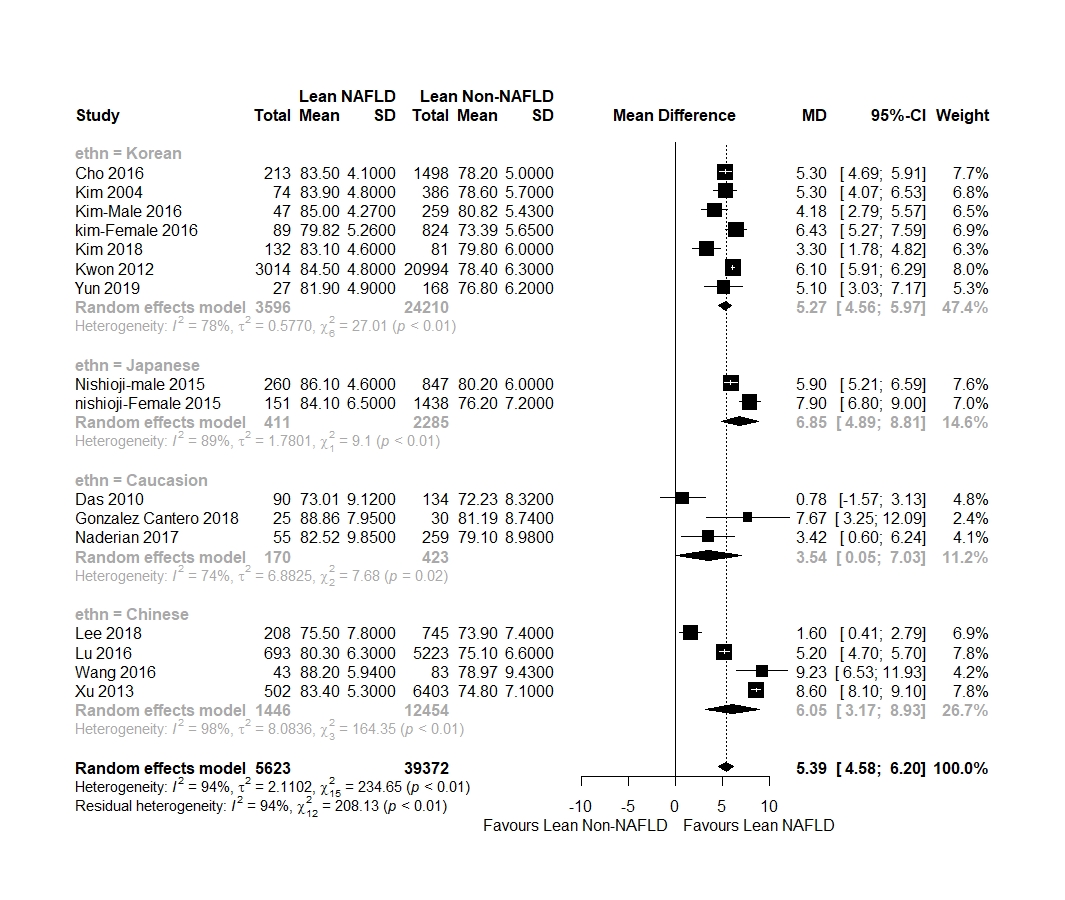


**Supplementary Figure 4A. Forest plot for waist circumference- Subgroup with respect to ethnicity.**


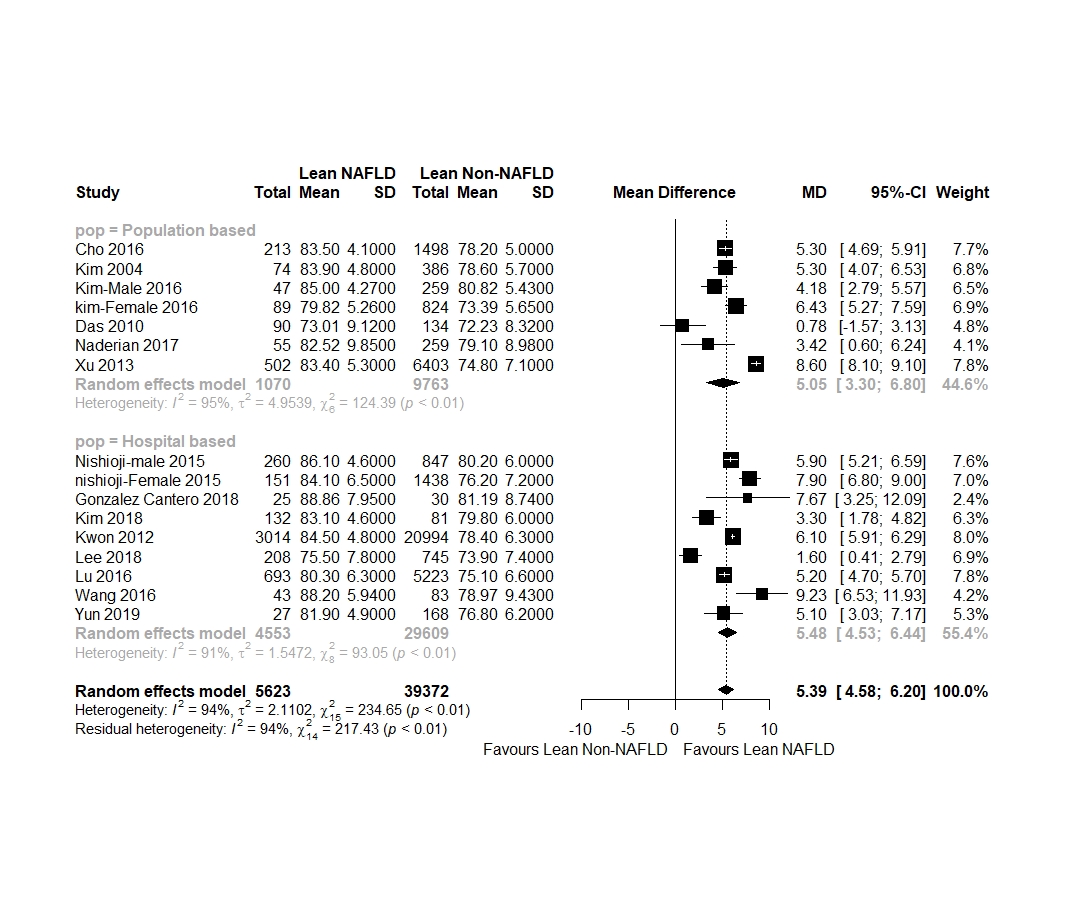


**Supplementary figure 4B. Forest plot for waist circumference- Subgroup with respect to population.**


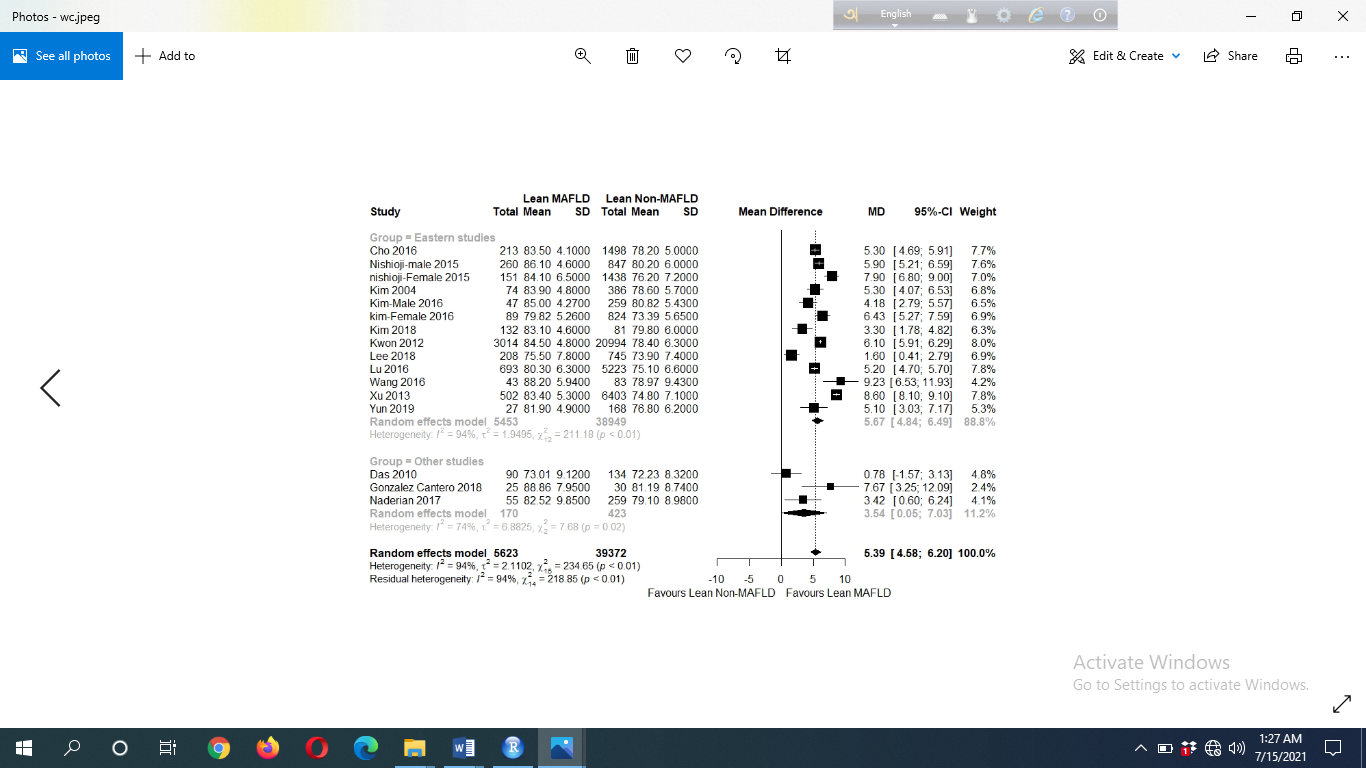


**Supplementary figure 4C. Forest plot for waist circumference- Subgroup with respect to Eastern and other studies.**


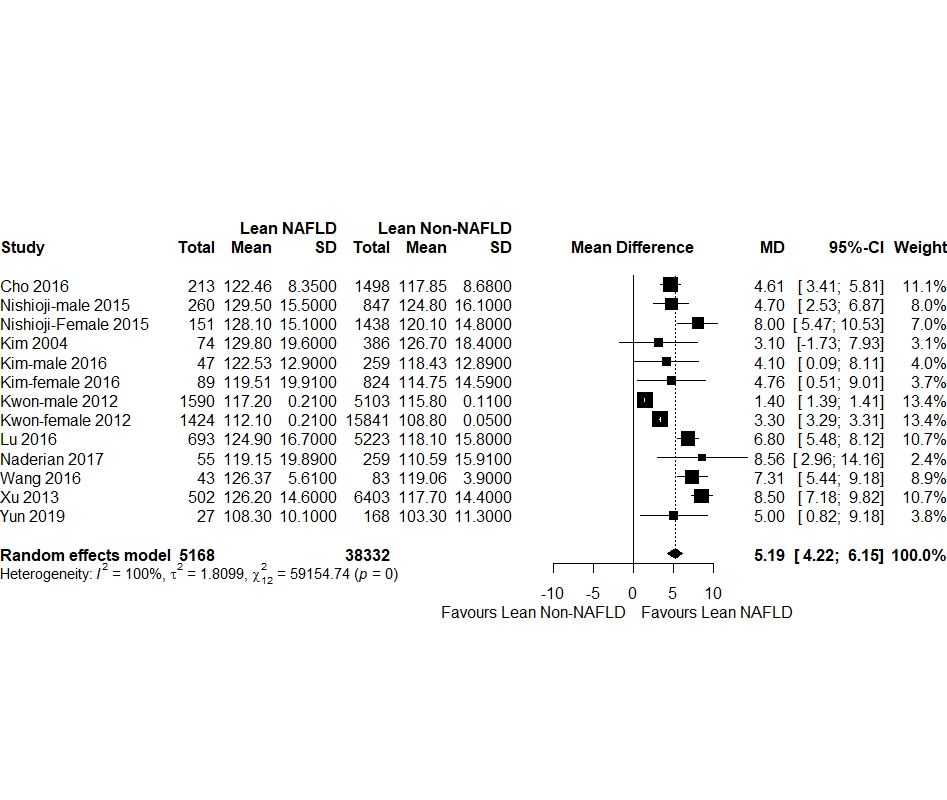


**Figure 5. Forest plot for systolic blood pressure**


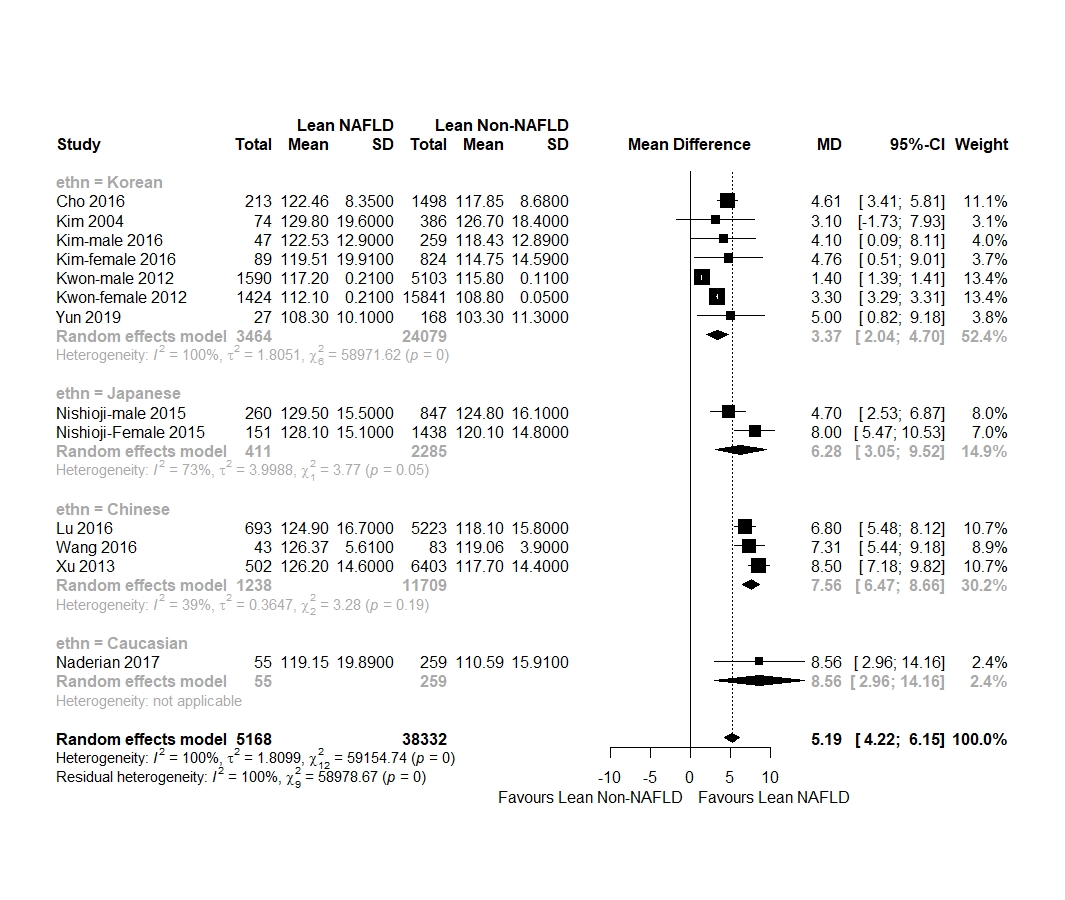


**Supplementary Figure 5A: Forrest plot analysis for Systolic Blood Pressure- Subgroup with respect to ethnicity.**


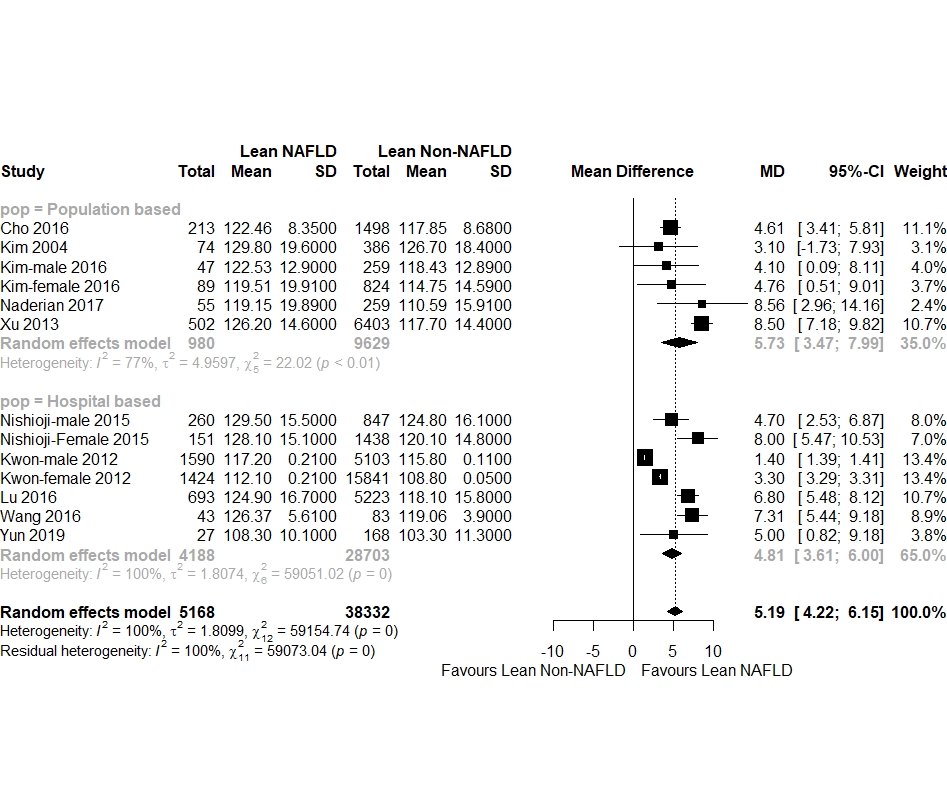


**Supplementary Figure 5B: Forrest plot analysis for Systolic Blood Pressure -Subgroup with respect to population.**


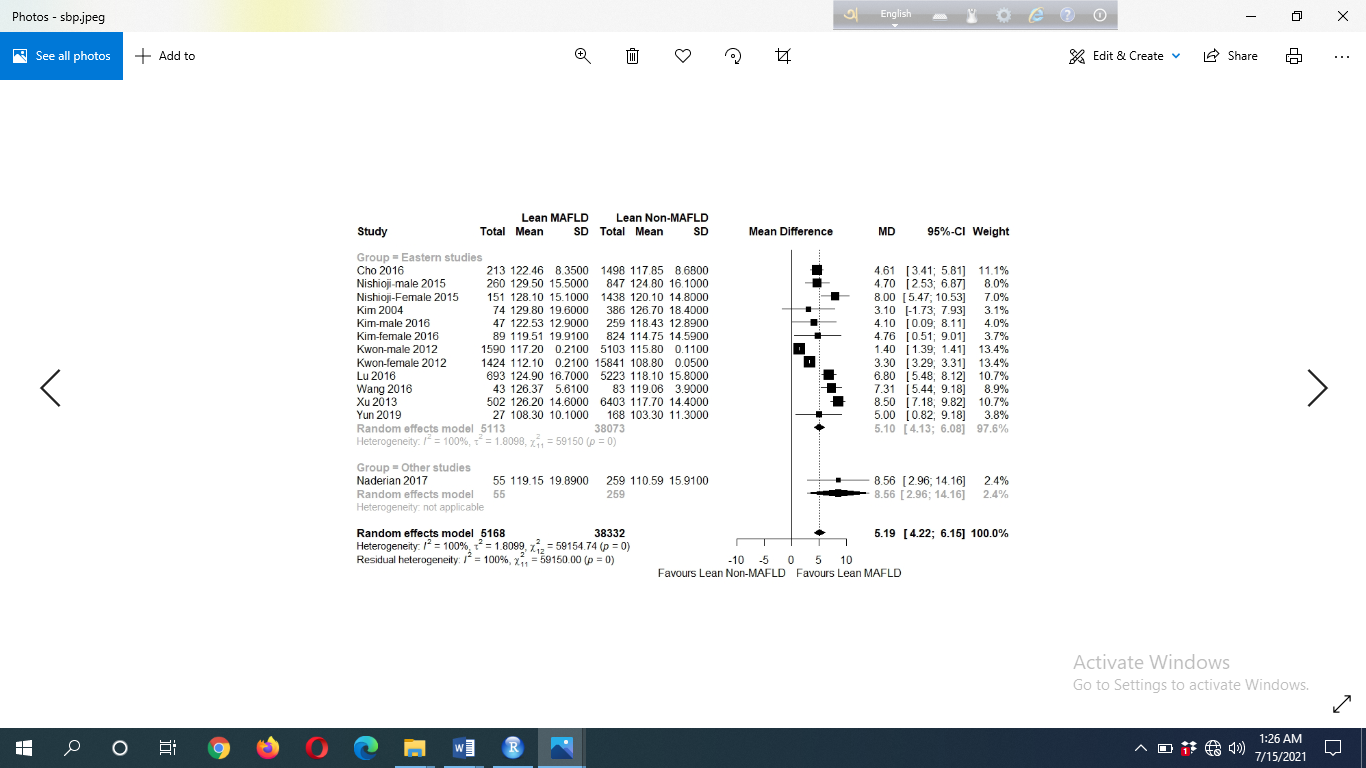


**Supplementary Figure 5C: Forrest plot analysis for Systolic Blood Pressure -Subgroup with respect to Eastern and other studies.**


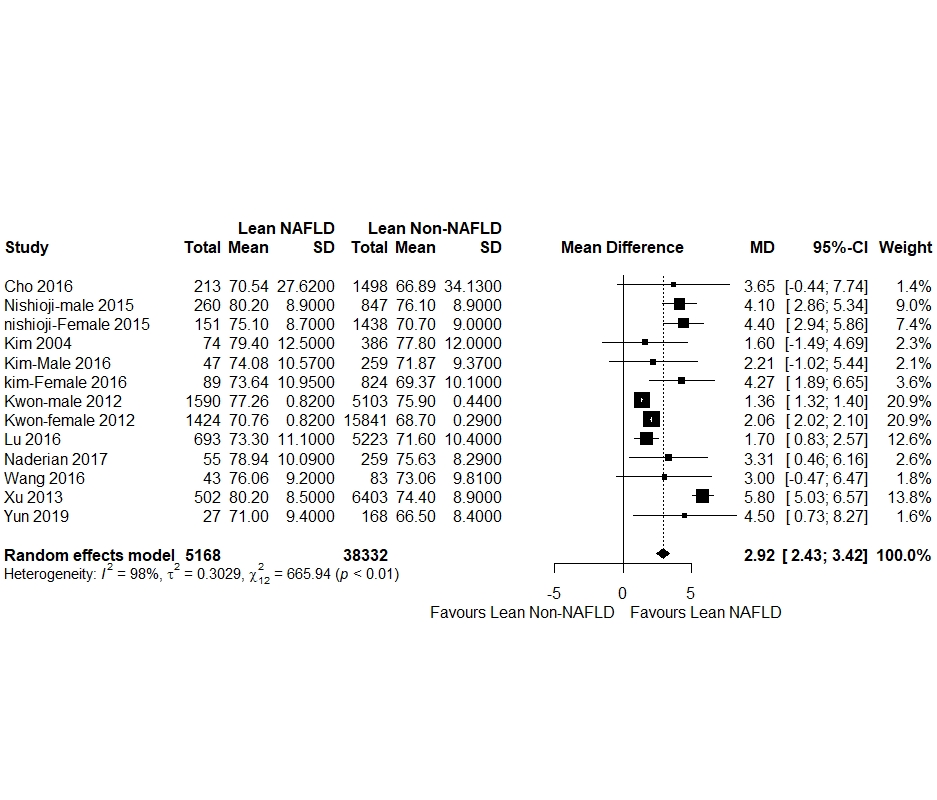


**Figure 6. Forrest plot analysis for diastolic blood pressure**


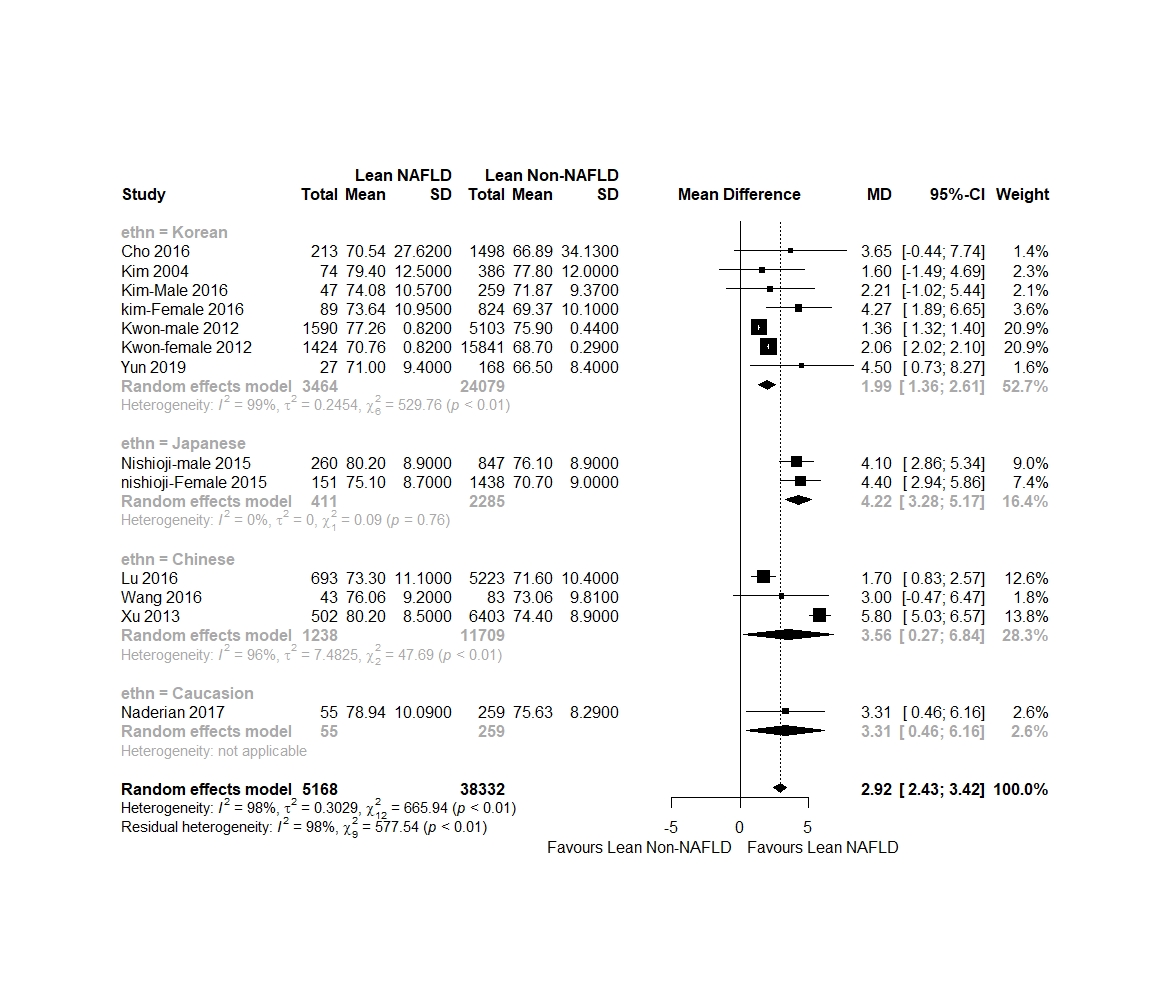


**Supplementary Figure 6A. Forrest plot analysis for diastolic blood pressure- Subgroup analysis with respect to ethnicity**


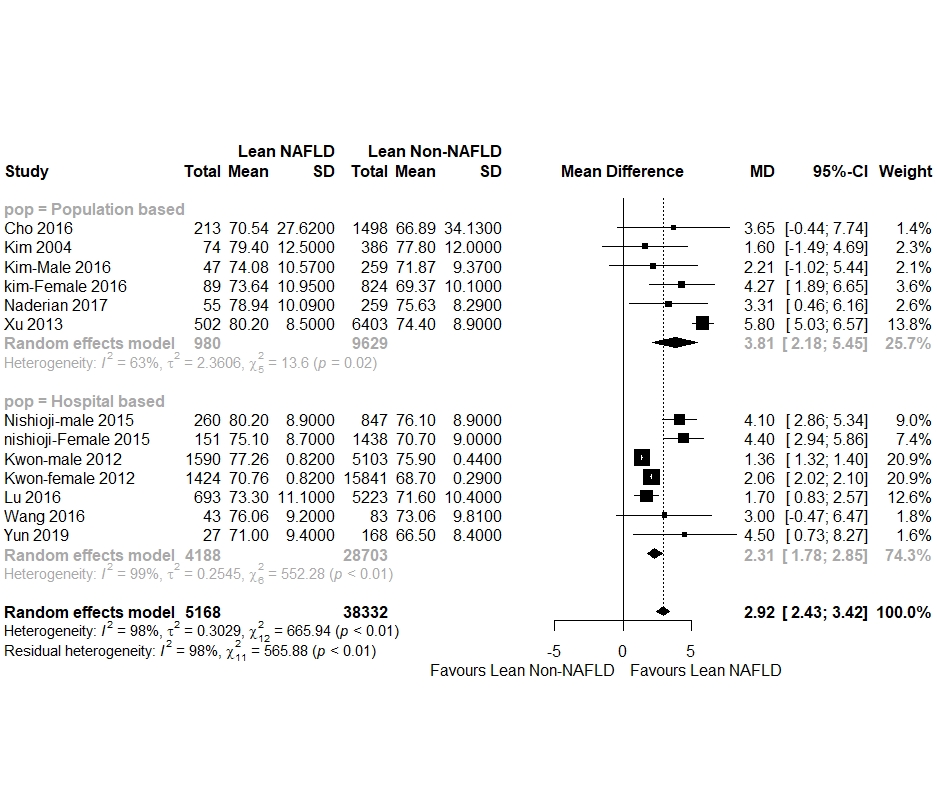


**Supplementary Figure 6B. Forrest plot analysis for diastolic blood pressure- Subgroup with respect to population.**


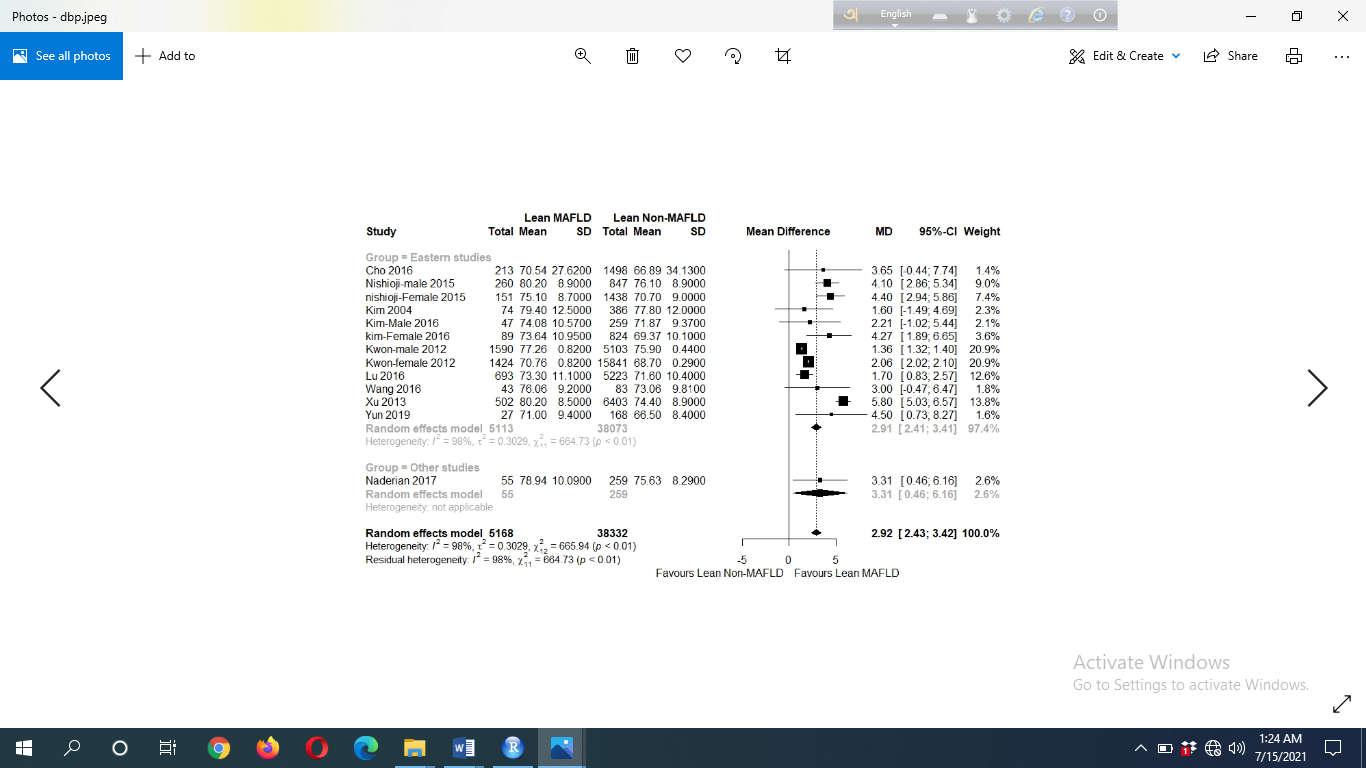


**Supplementary Figure 6C. Forrest plot analysis for diastolic blood pressure- Subgroup with respect to Eastern and other studies.**


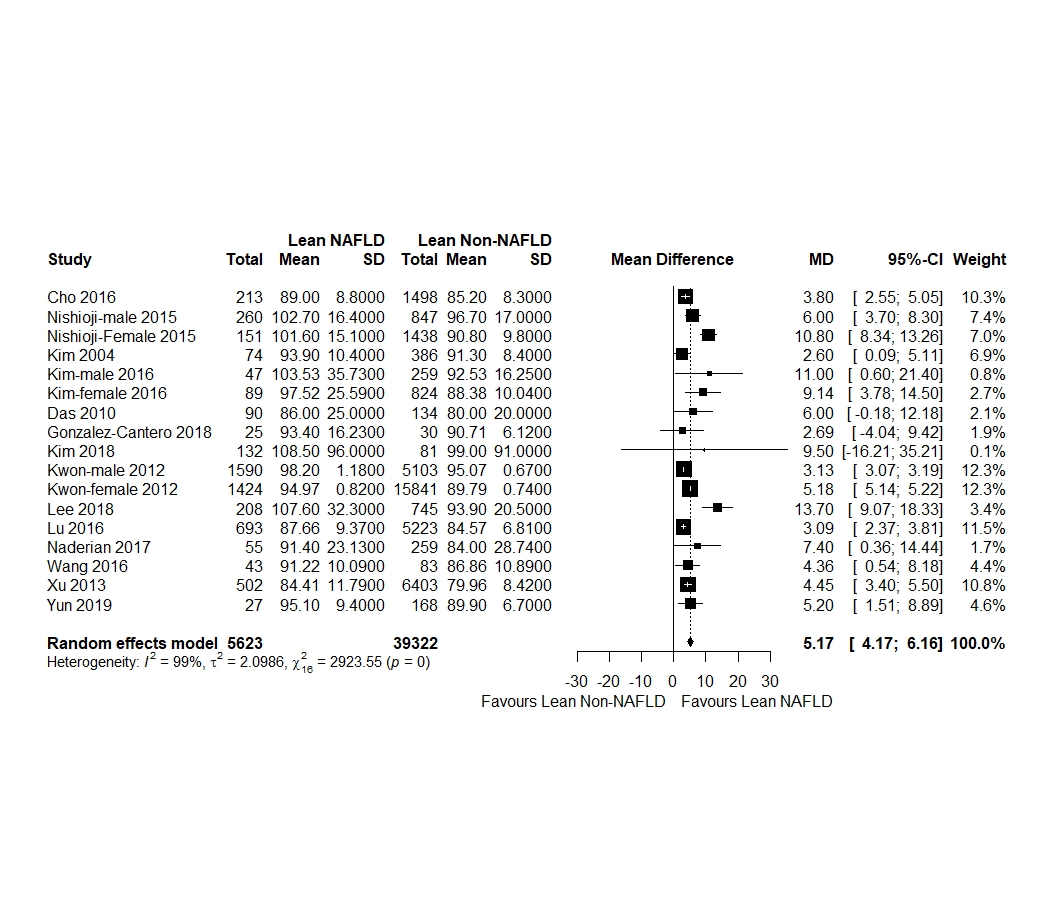


**Figure 7. Forest plot for fasting blood sugar.**


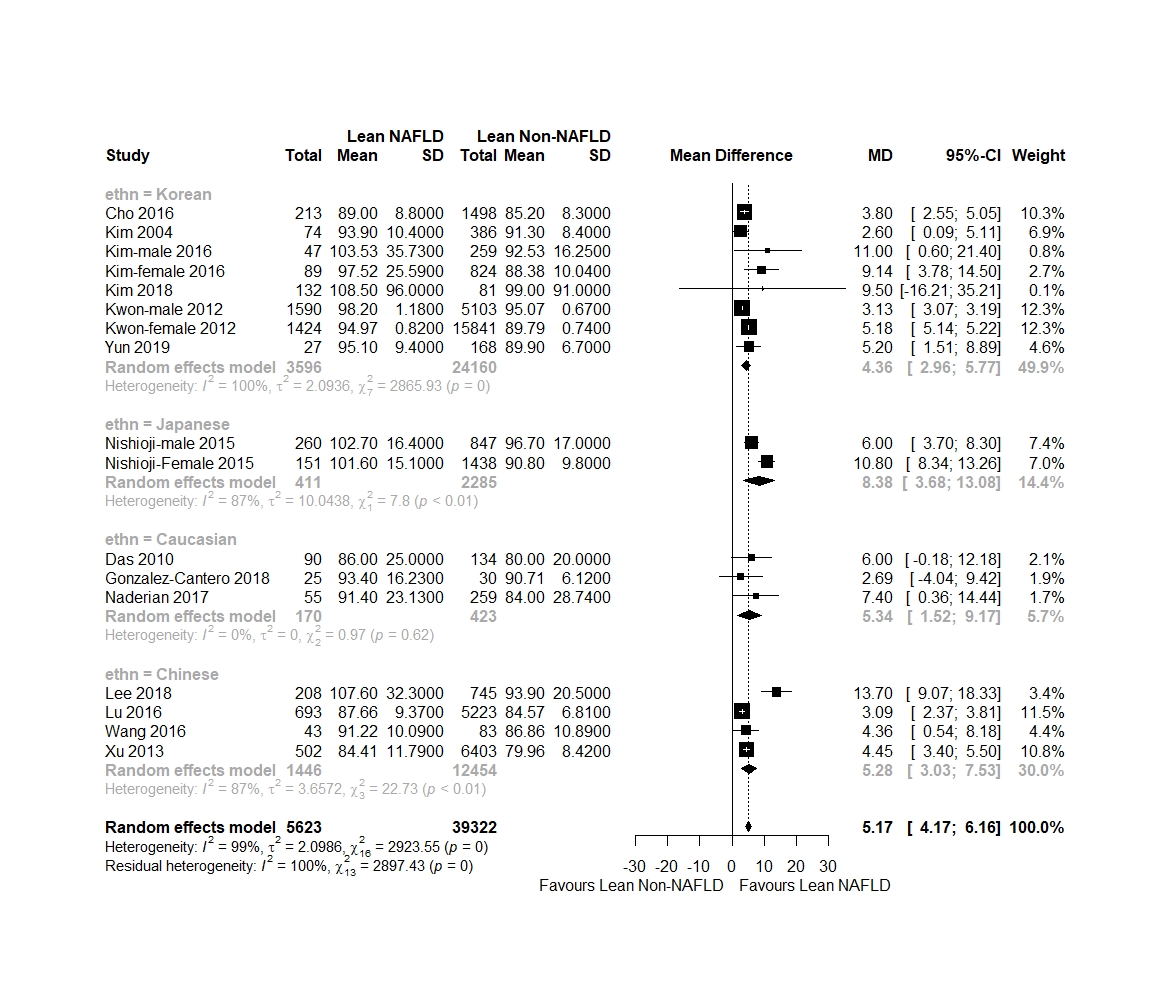


**Supplementary figure 7A. Forrest plot analysis for fasting blood sugar- Subgroup with respect to ethnicity**


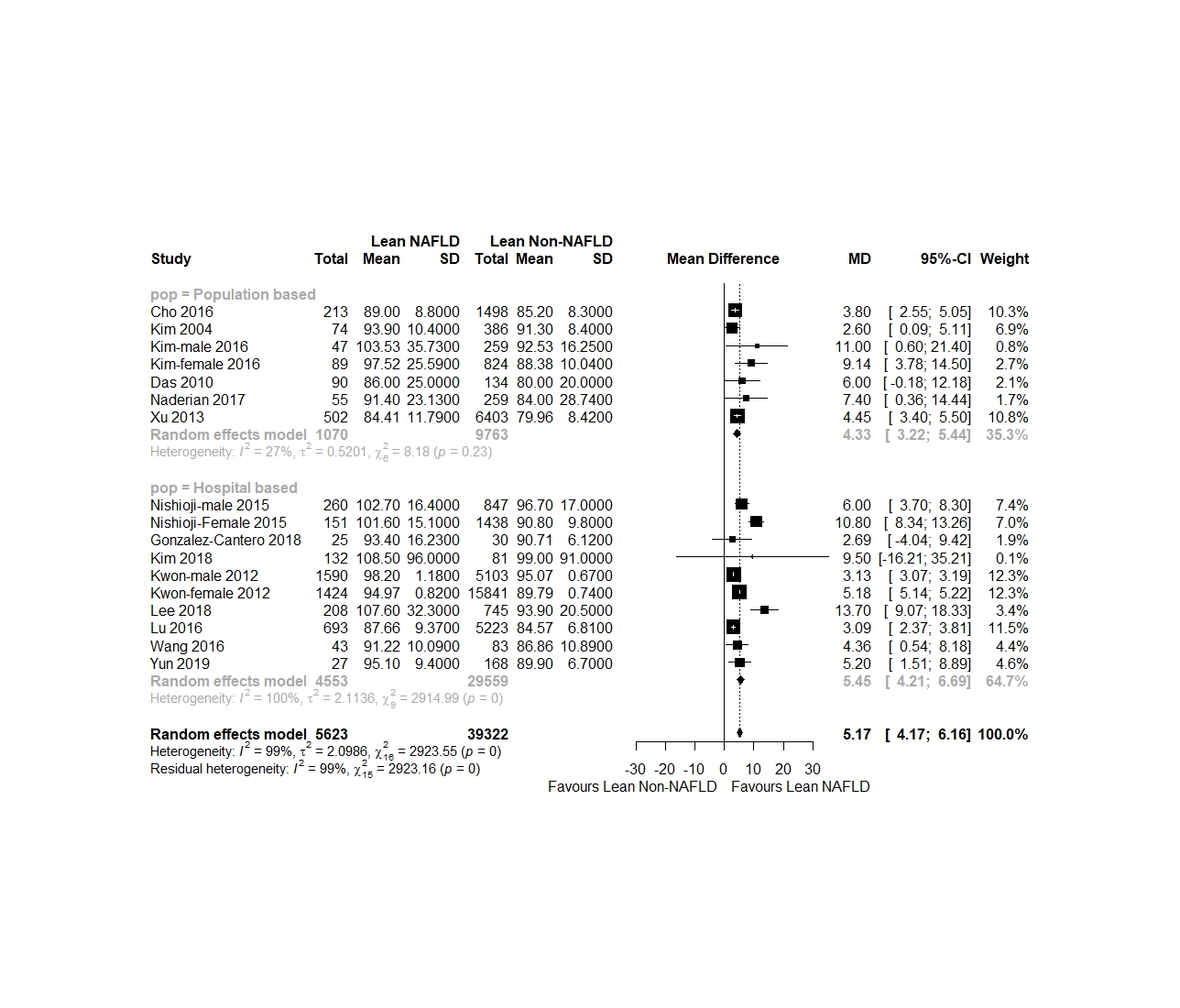


**Supplementary figure 7B. Forrest plot analysis for fasting blood sugar- Subgroup with respect to population**


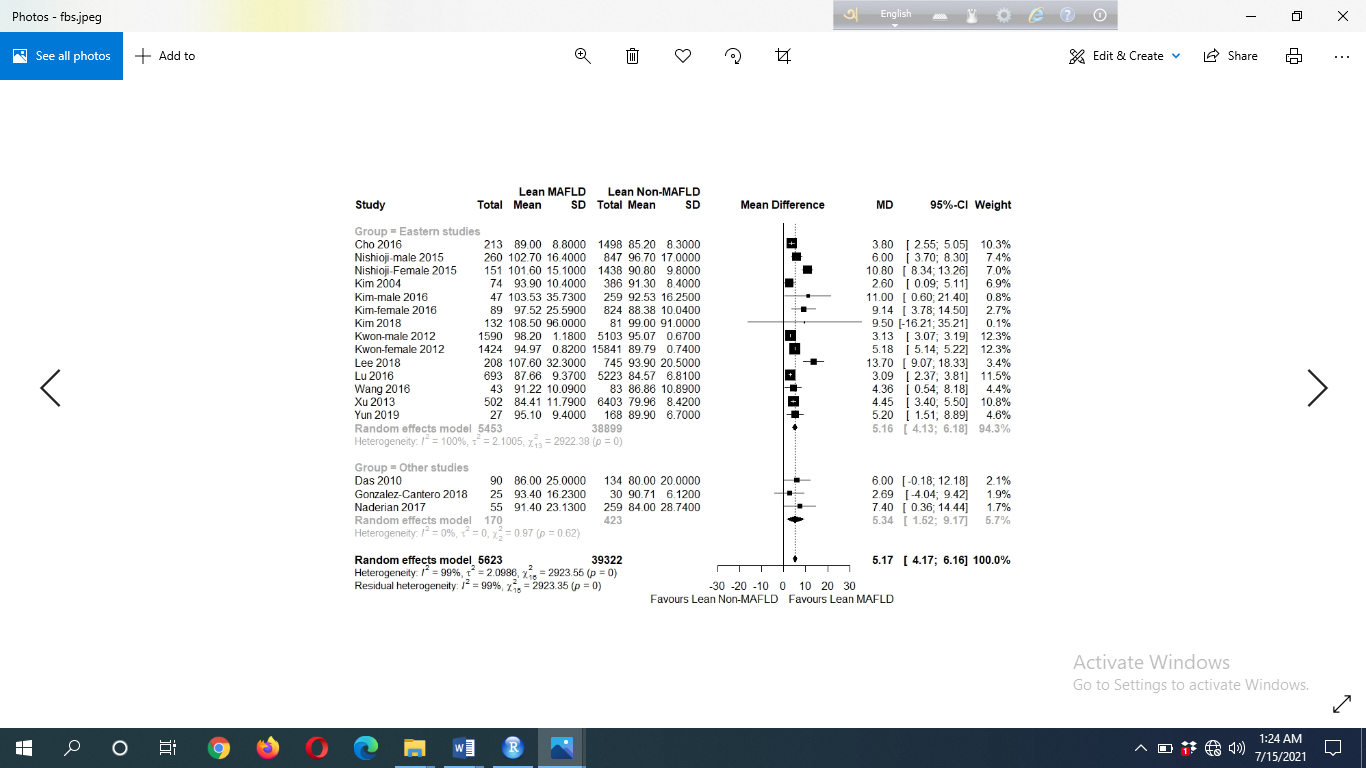


**Supplementary figure 7C. Forrest plot analysis for fasting blood sugar- Subgroup with respect to Eastern and other studies.**


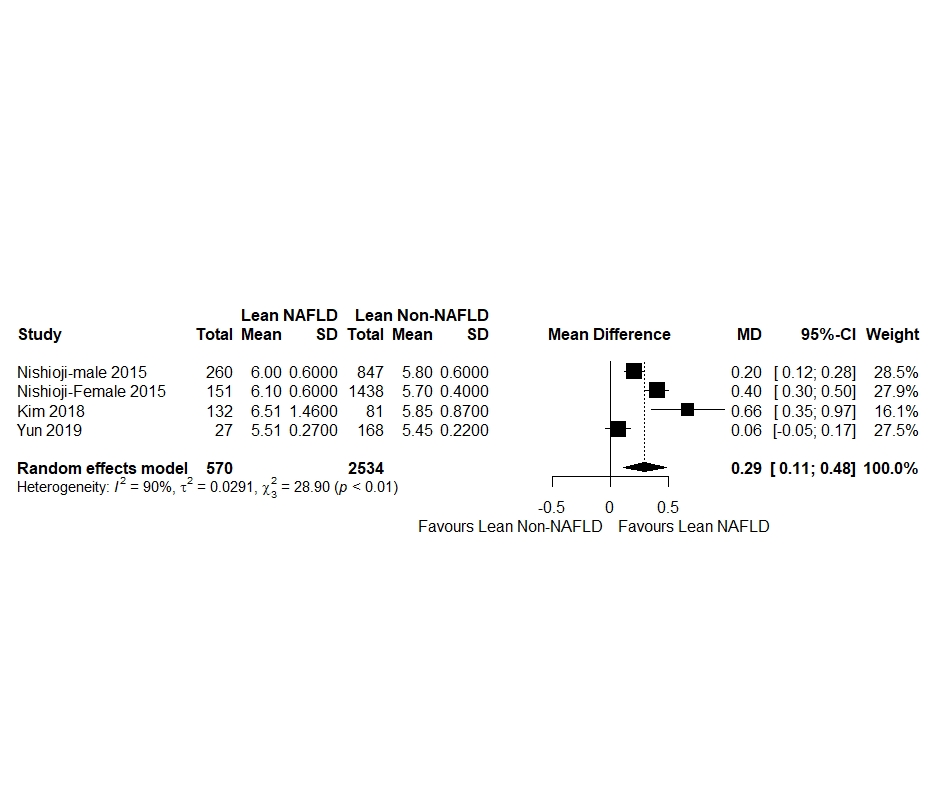


**Figure 8: Forest plot for HbA1c**


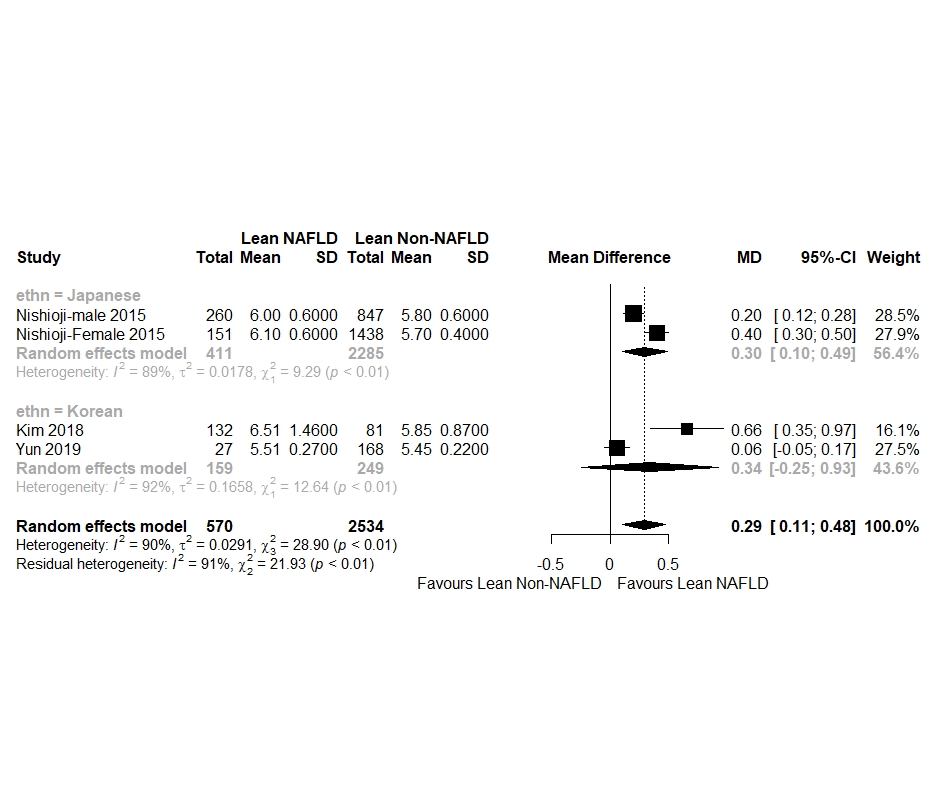


**Supplementary figure 8A. Forrest plot for HbA1c- Subgroup analysis with respect to ethnicity**


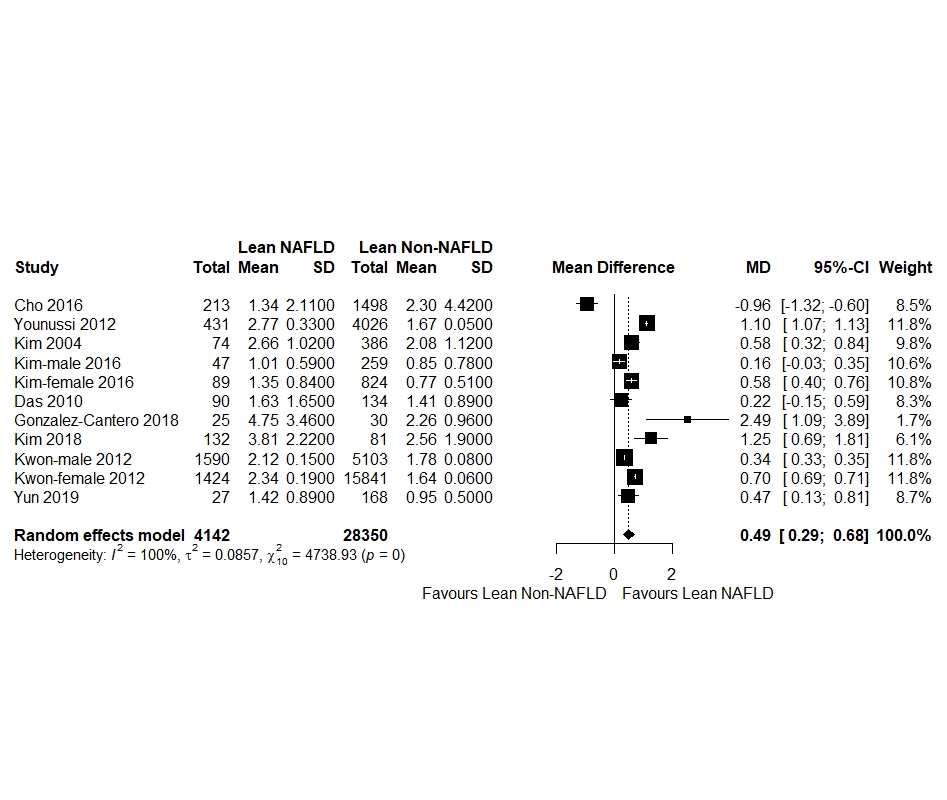


**Figure 9. Forest plot for HOMA-IR**


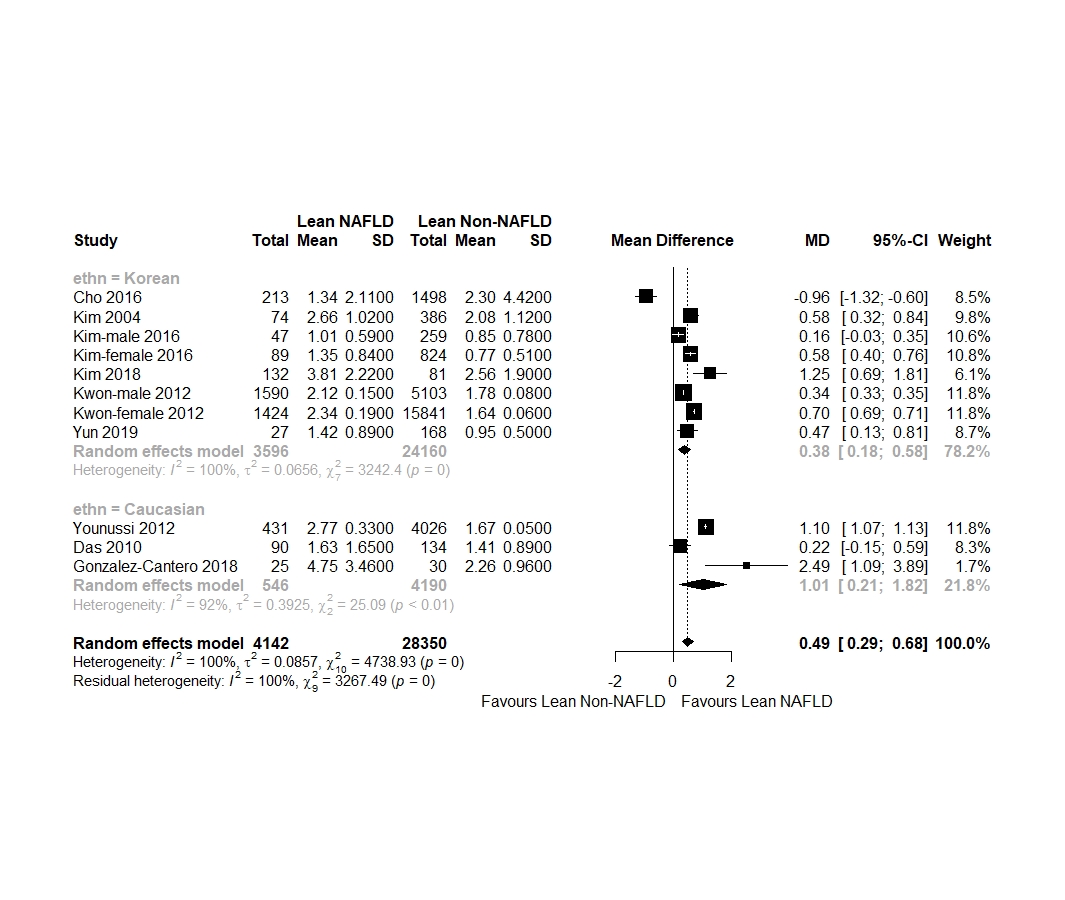


**Supplementary figure 9A. Forest plot for HOMA-IR- Subgroup with respect to ethnicity.**


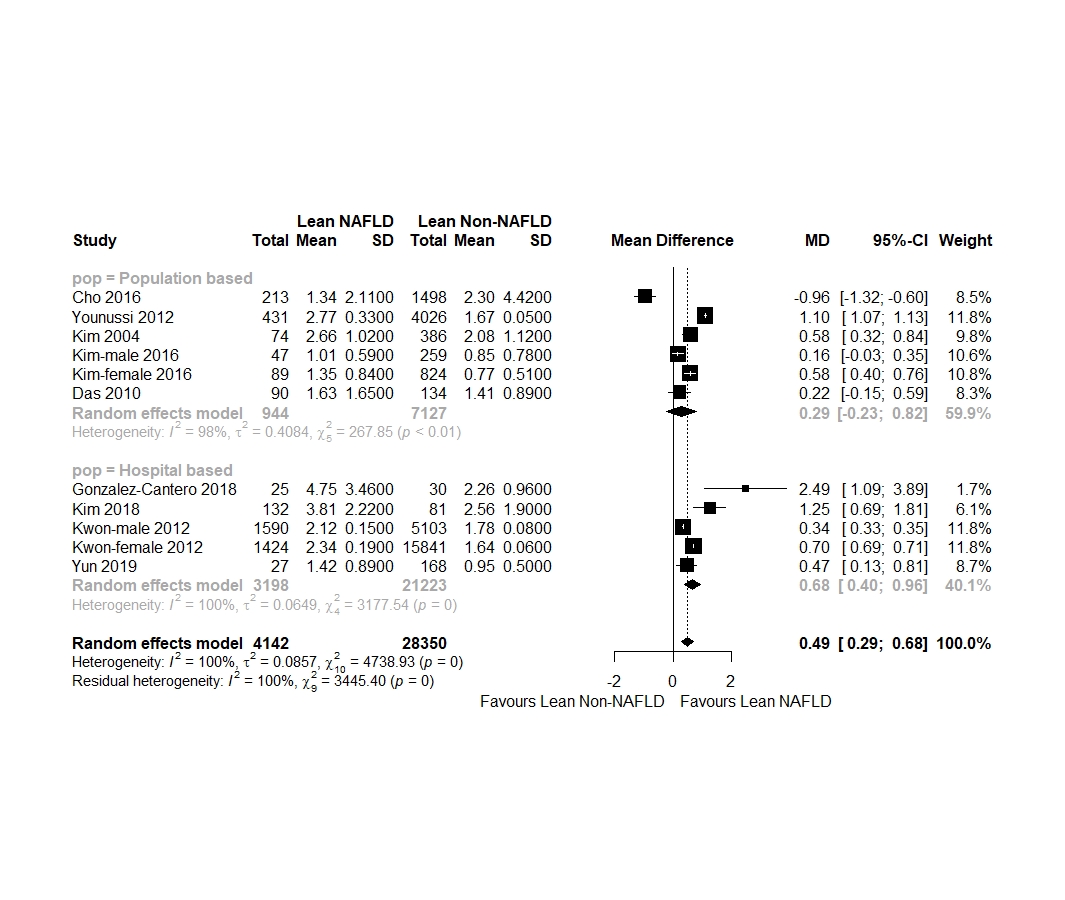


**Supplementary figure 9B. Forest plot for HOMA-IR-Subgroup with respect to population.**


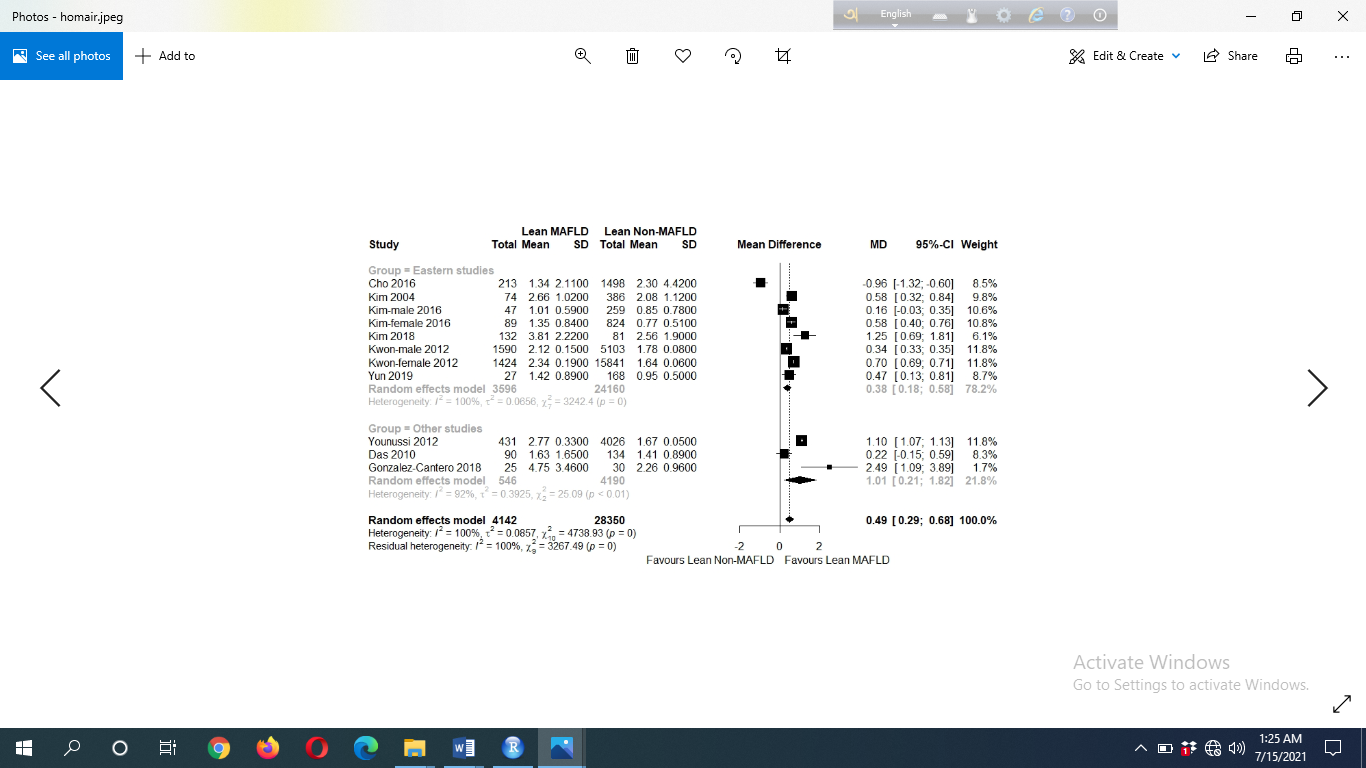


**Supplementary figure 9C. Forest plot for HOMA-IR-Subgroup with respect to Eastern and other studies.**


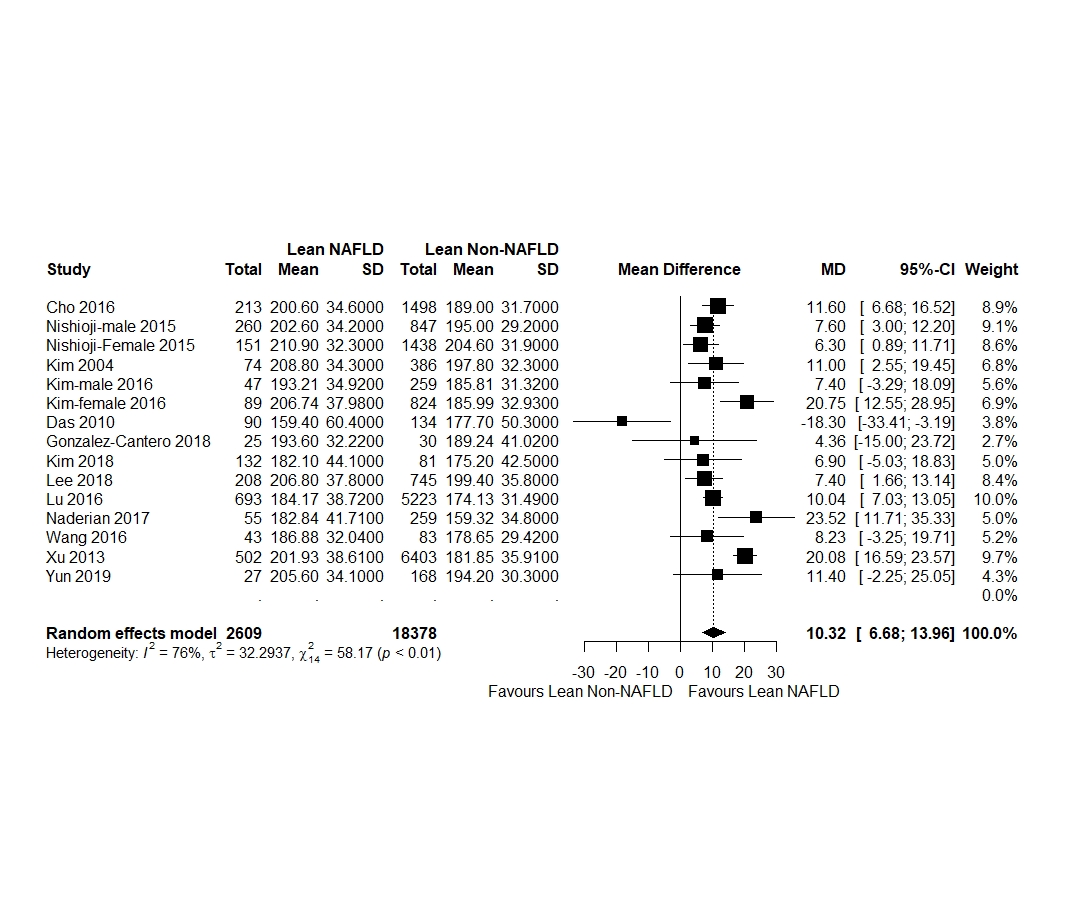


**Figure 10. Forest plot for total cholesterol**


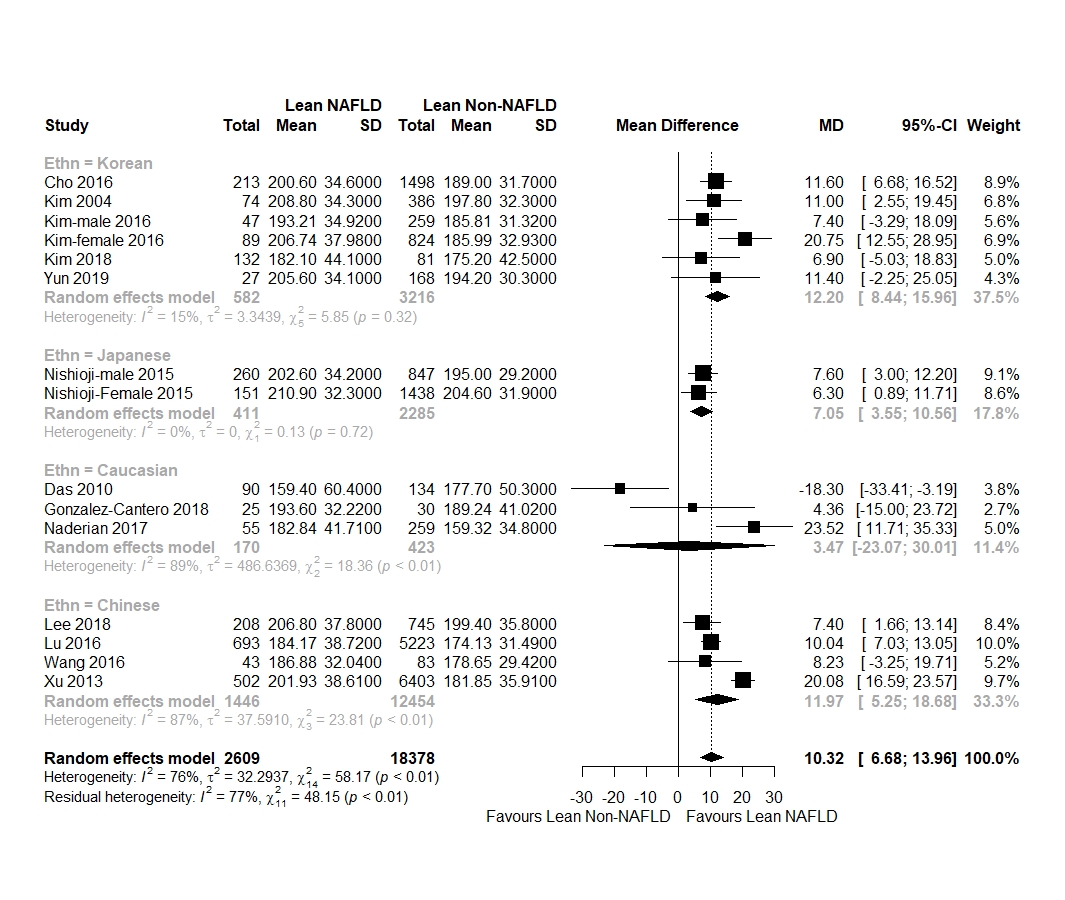


**Supplementary Figure 10A. Forrest plot for total cholesterol- Subgroup with respect to ethnicity.**


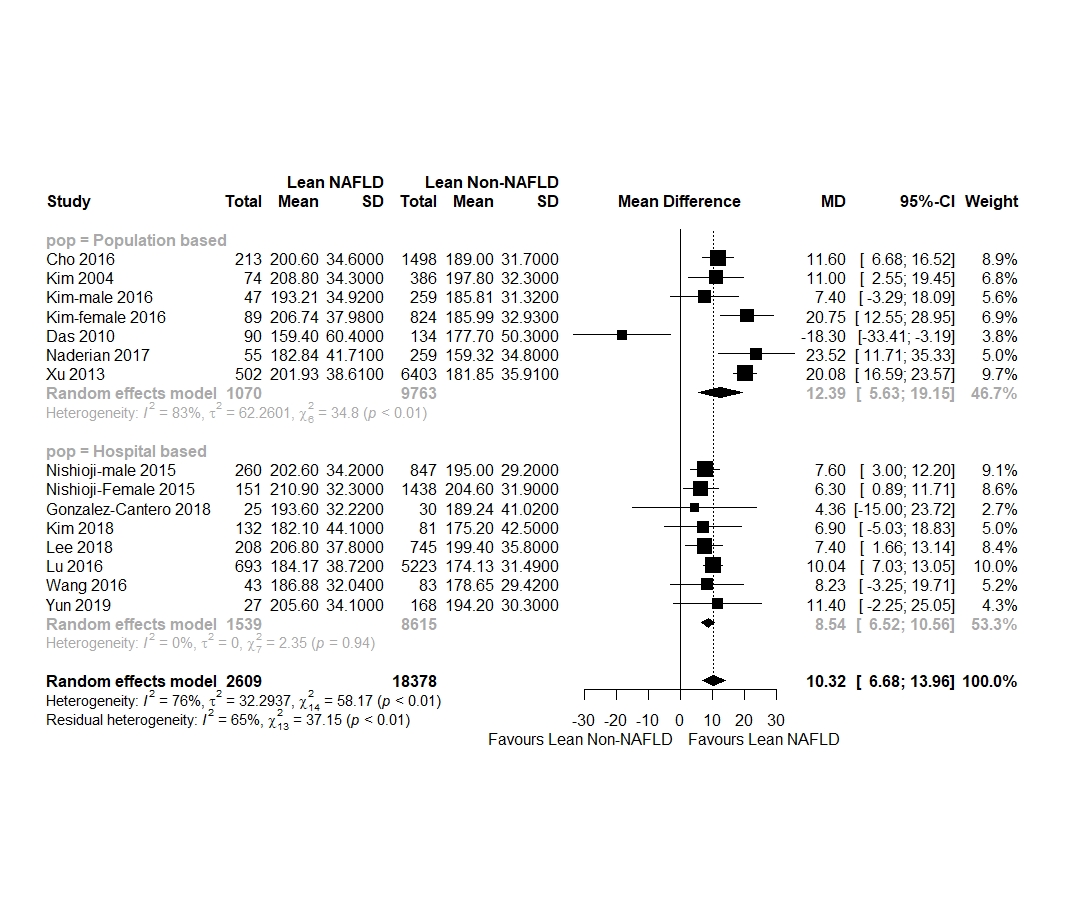


**Supplementary Figure 10B. Forrest plot for total cholesterol- Subgroup with respect to population**.


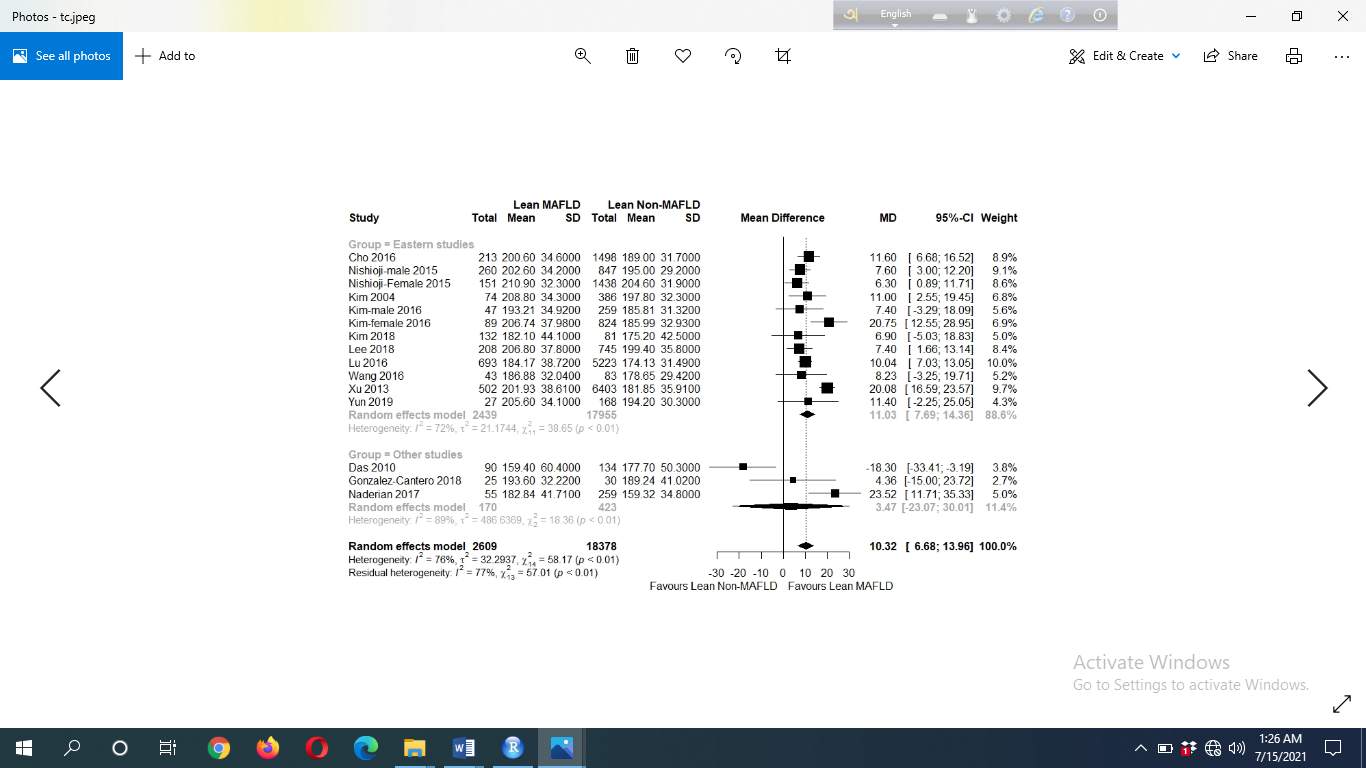


**Supplementary Figure 10C. Forrest plot for total cholesterol- Subgroup with respect to Eastern and other studies**


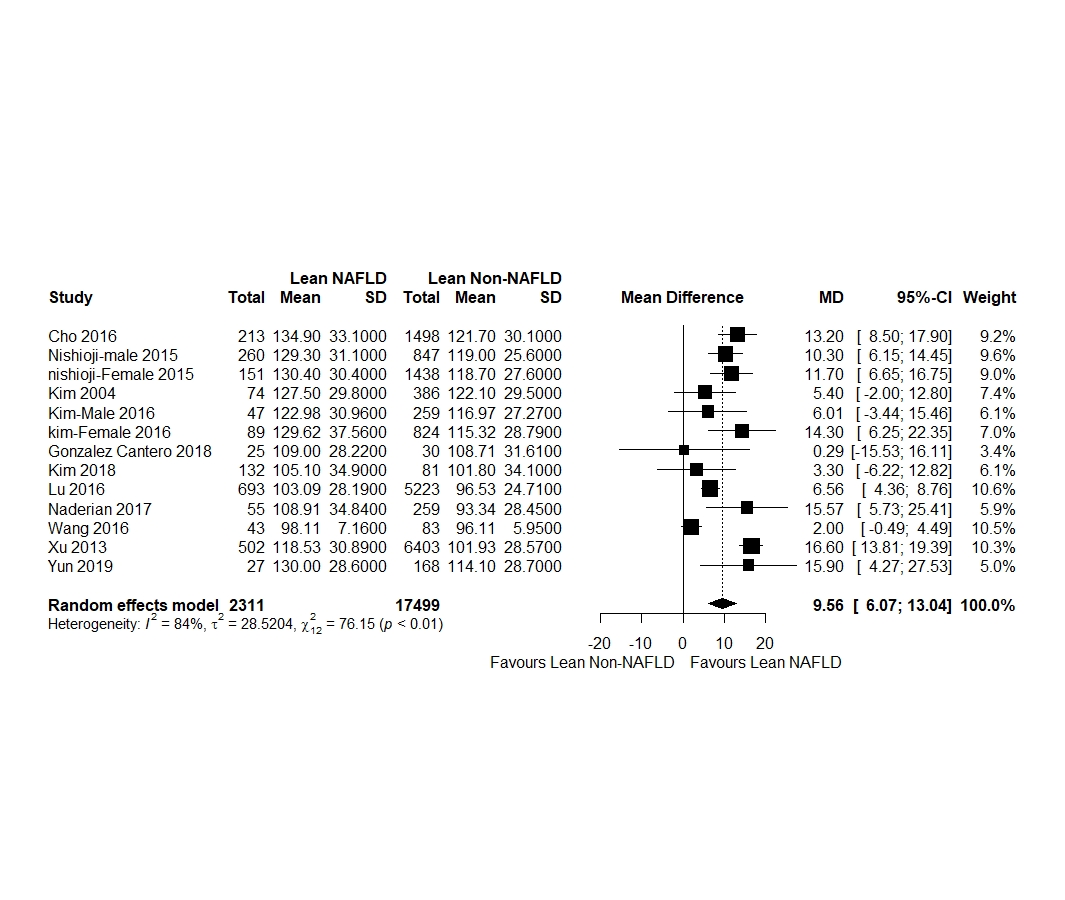


**Figure 11. Forrest plot for LDL**


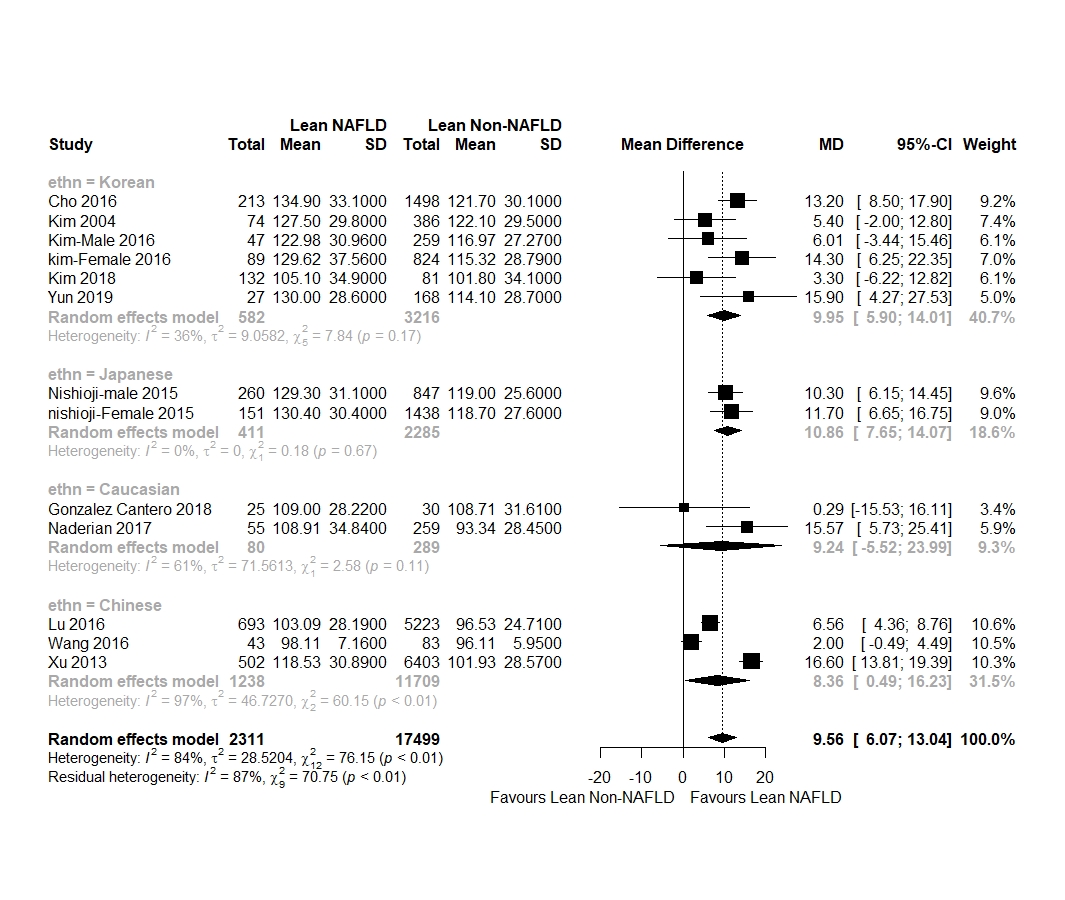


**Supplementary Figure 11A. Forrest plot for LDL- Subgroup with respect to ethnicity.**


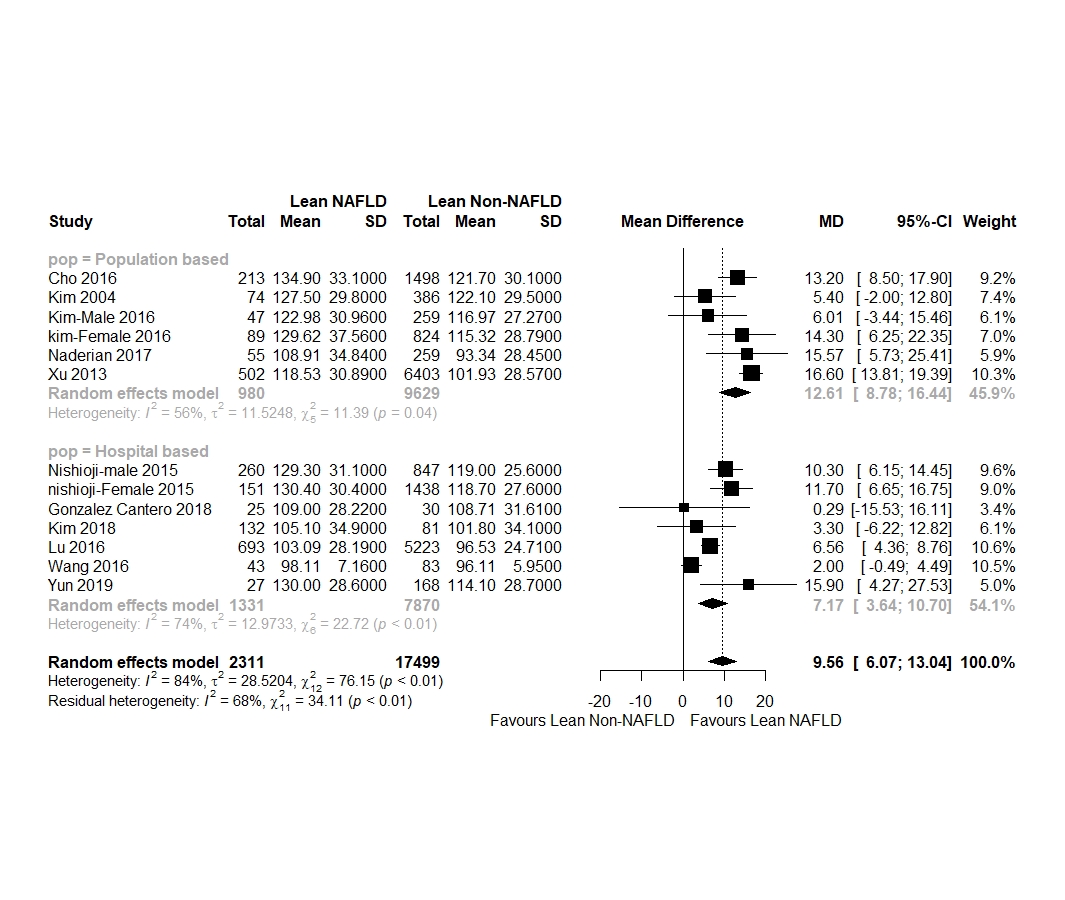


**Supplementary Figure 11B. Forrest plot for LDL- Subgroup with respect to population.**


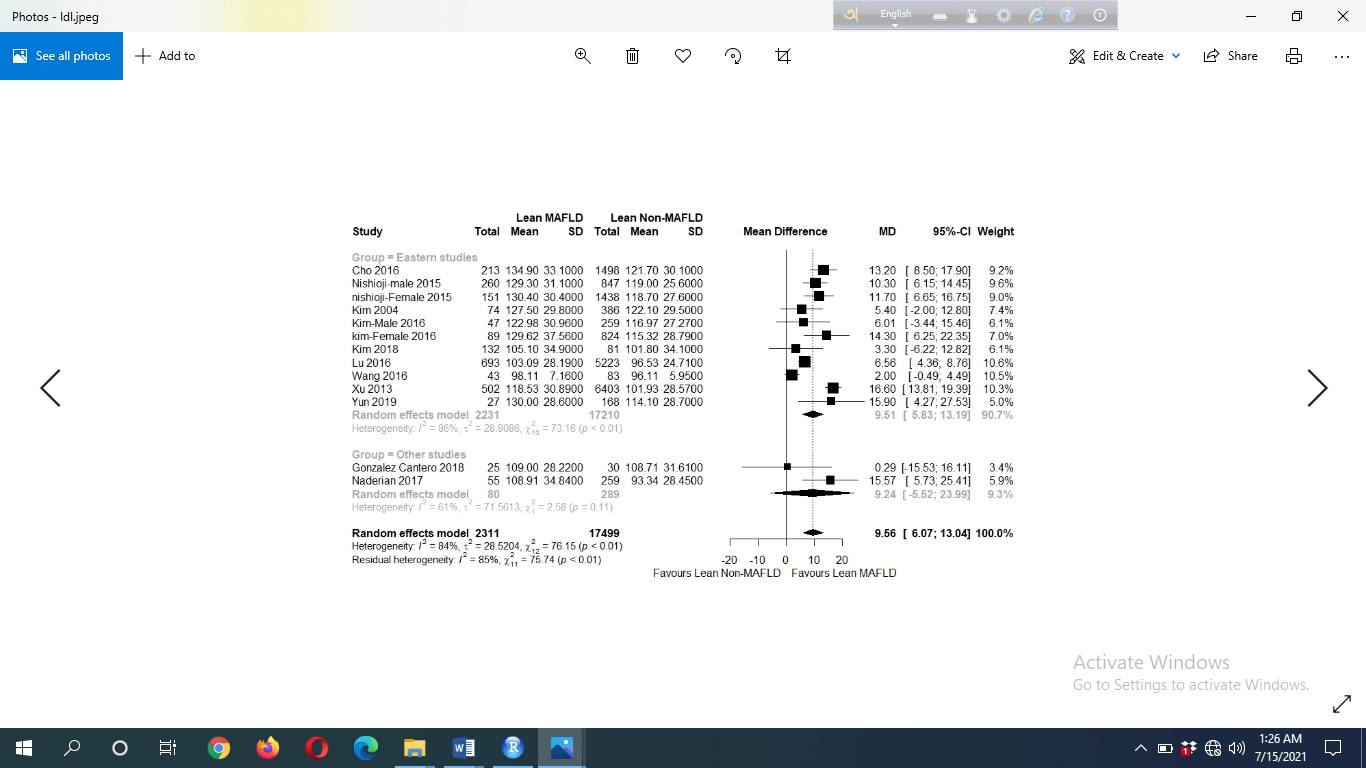


**Supplementary Figure 11C. Forrest plot for LDL- Subgroup with respect to Eastern and other studies.**


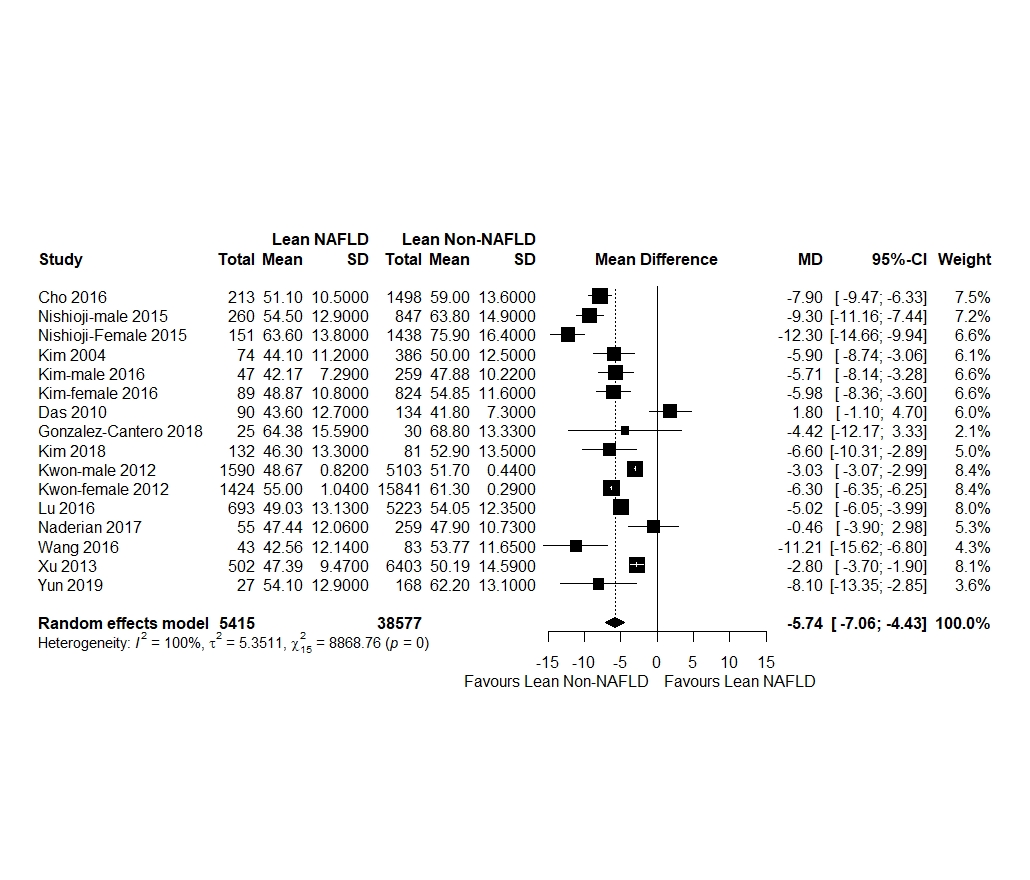


**Figure 12. Forest plot for HDL**


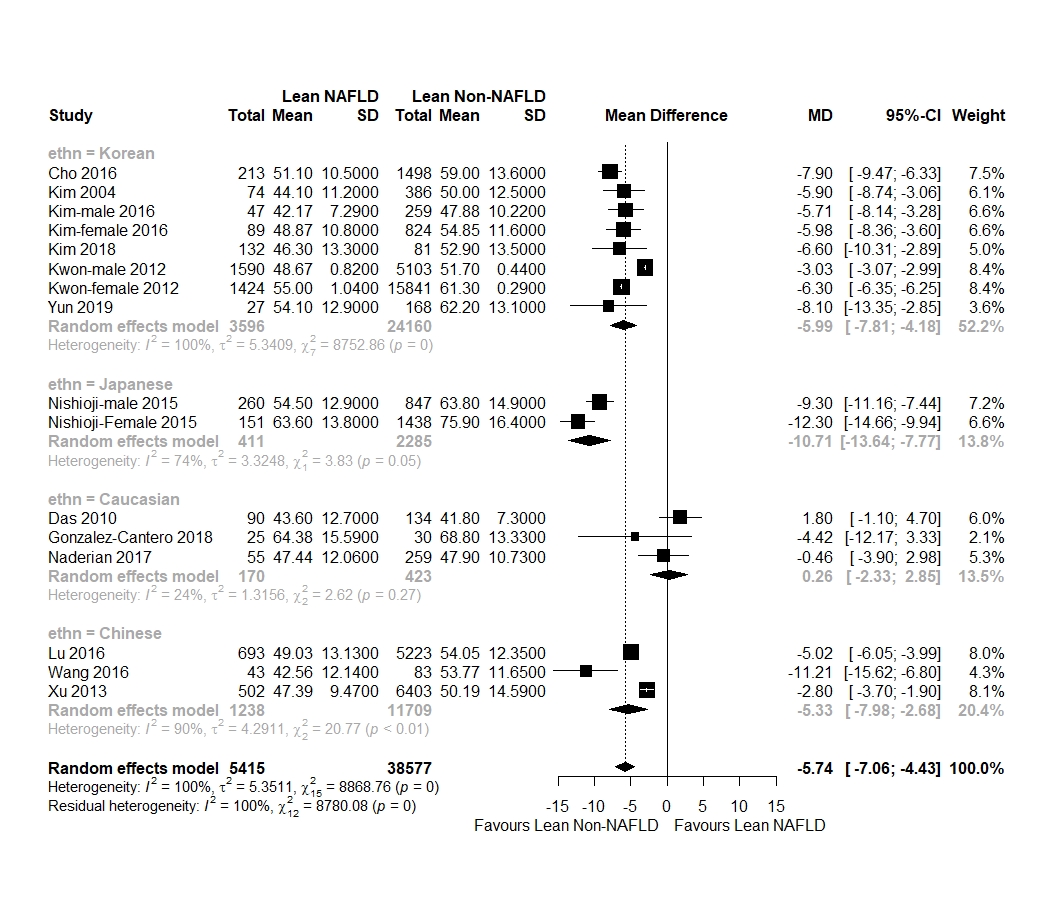


**Supplementary Figure 12A. Forest plot for HDL - Subgroup with respect to ethnicity**.


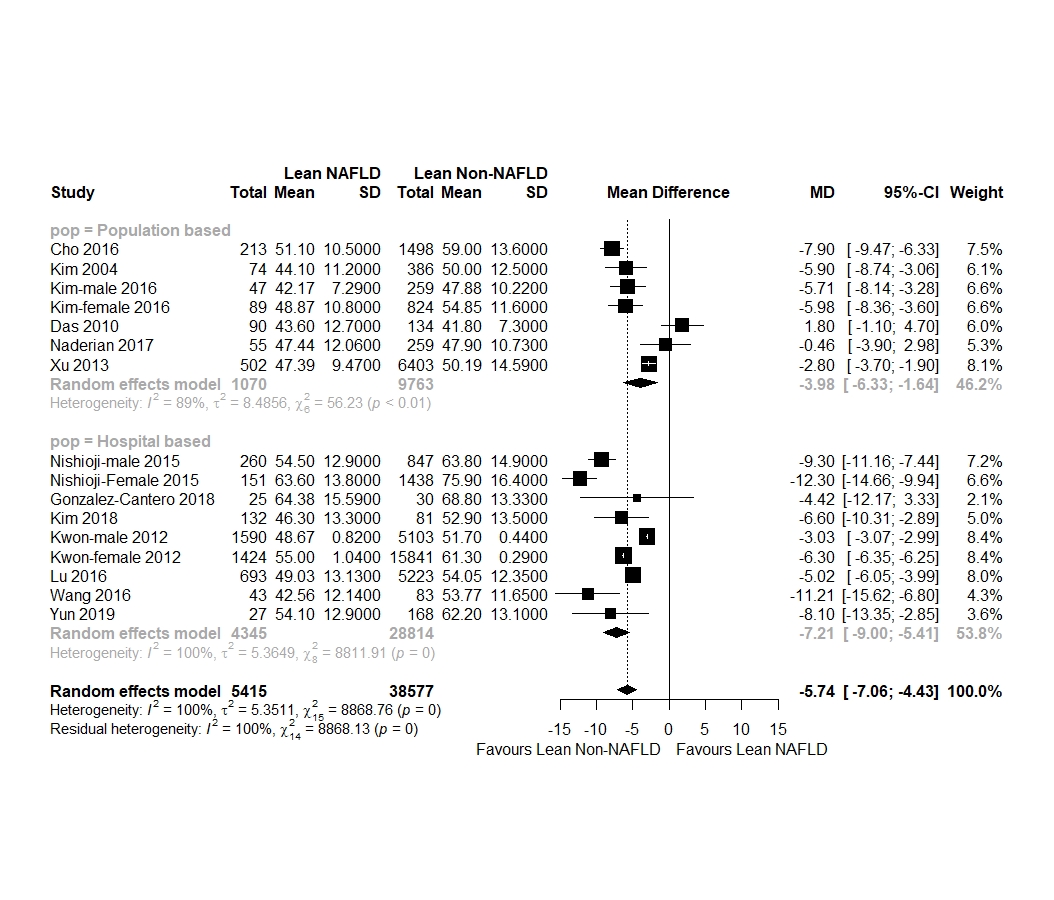


**Supplementary Figure 12B. Forest plot for HDL - Subgroup with respect to population.**


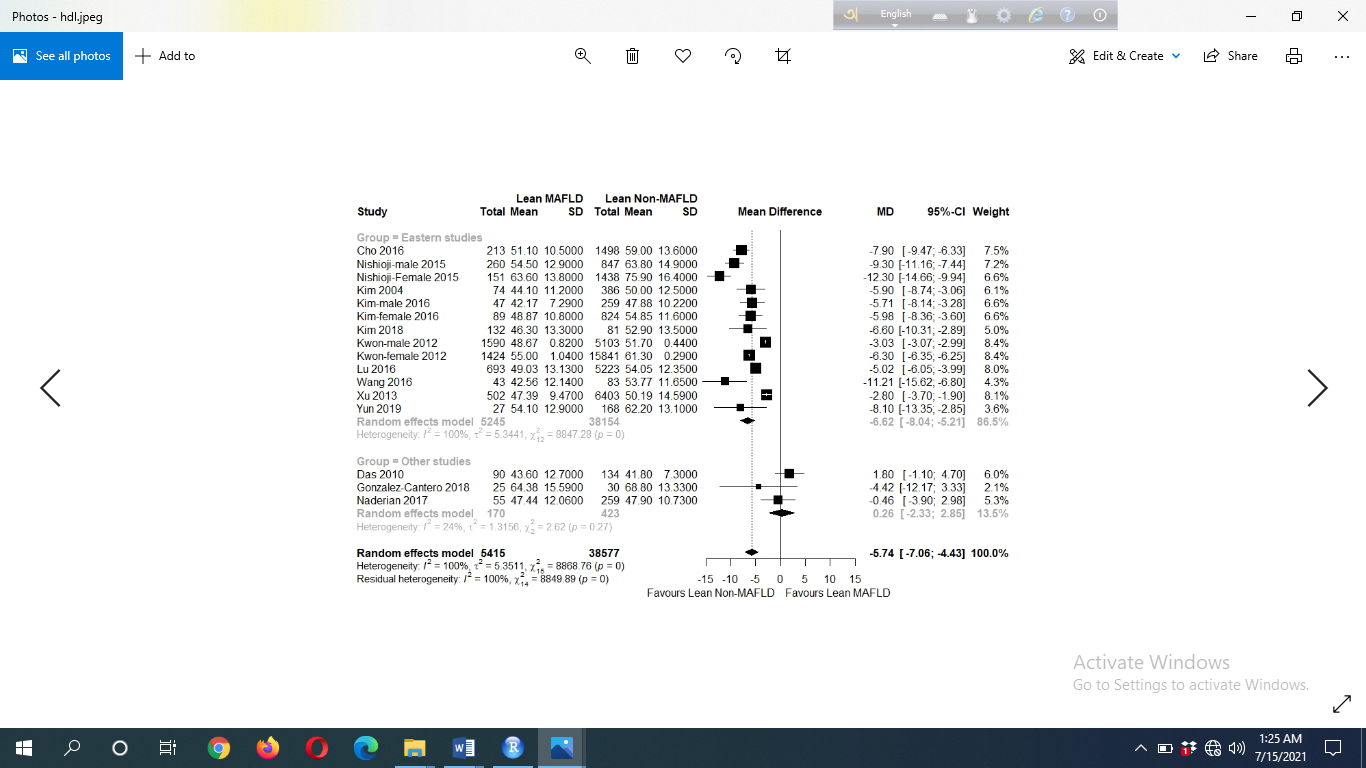


**Supplementary Figure 12C. Forest plot for HDL - Subgroup with respect to Eastern and other studies.**


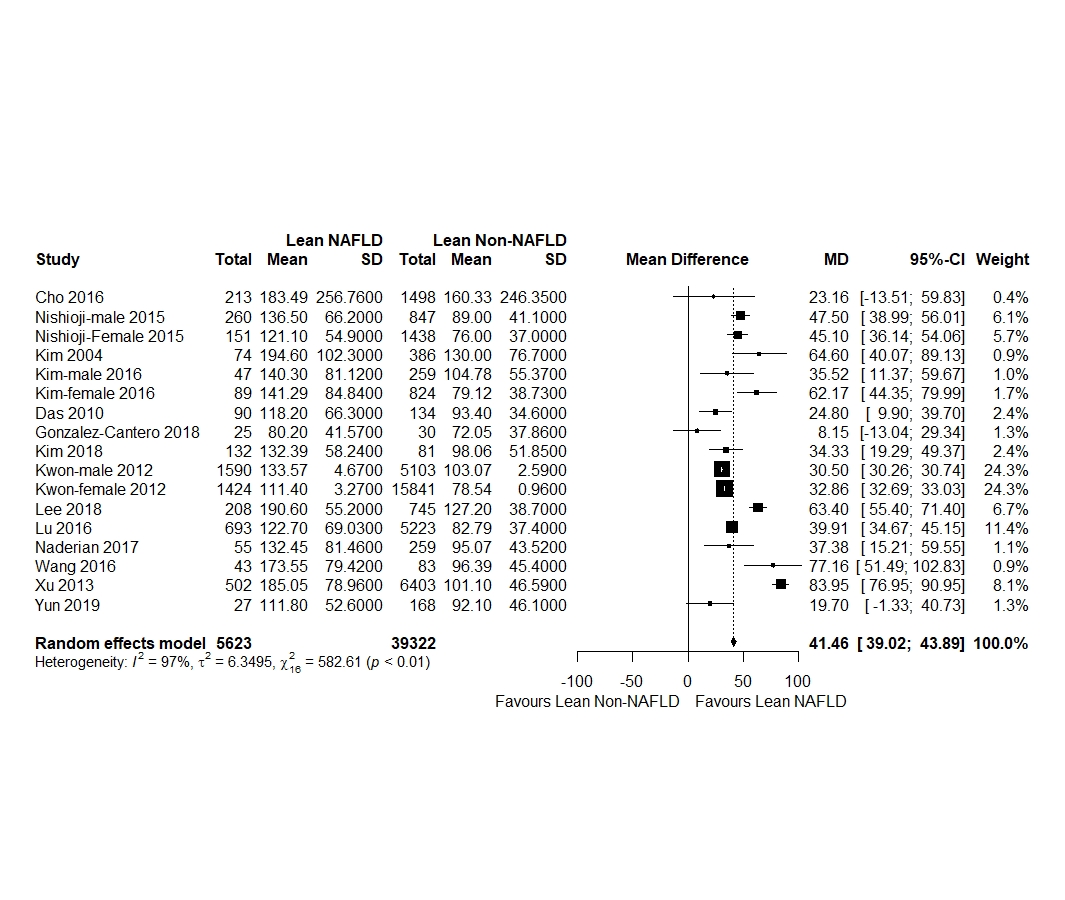


**Figure 13. Forest plot for TG**


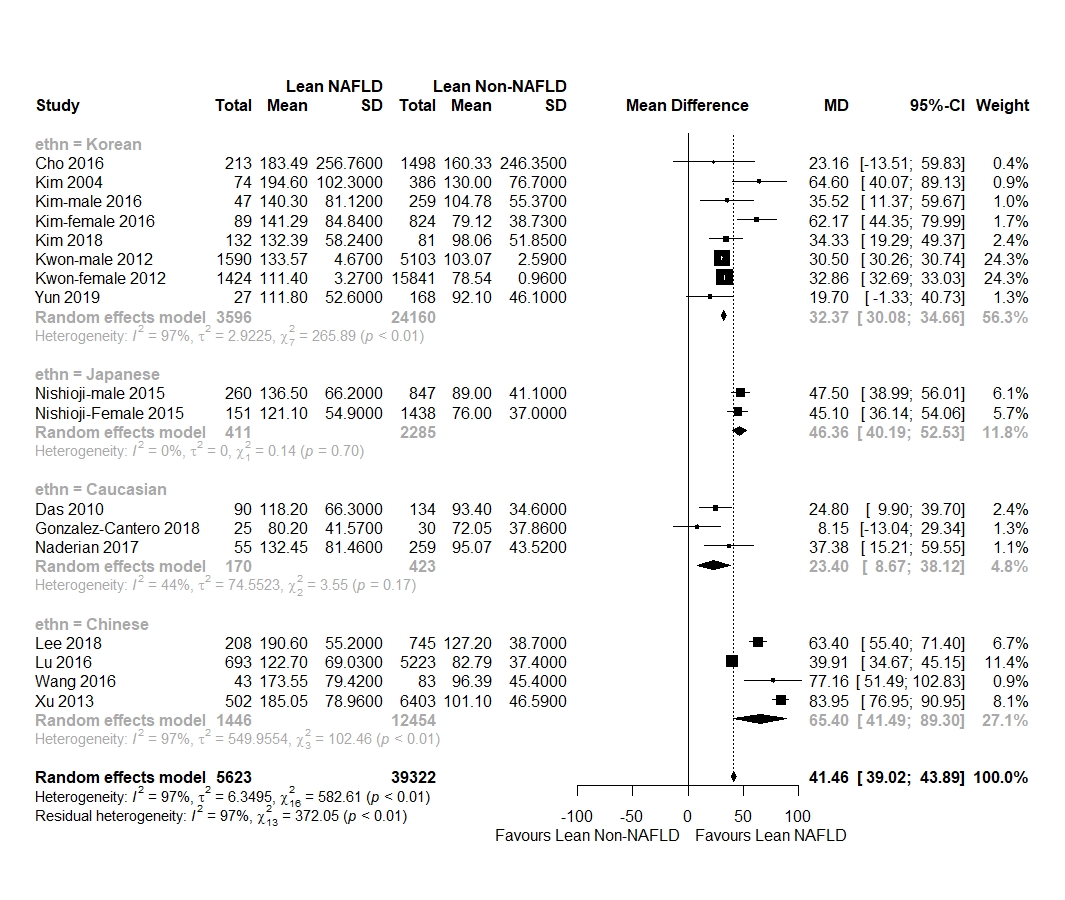


**Supplementary Figure 13A. Forrest plot for TG - Subgroup with respect to ethnicity.**


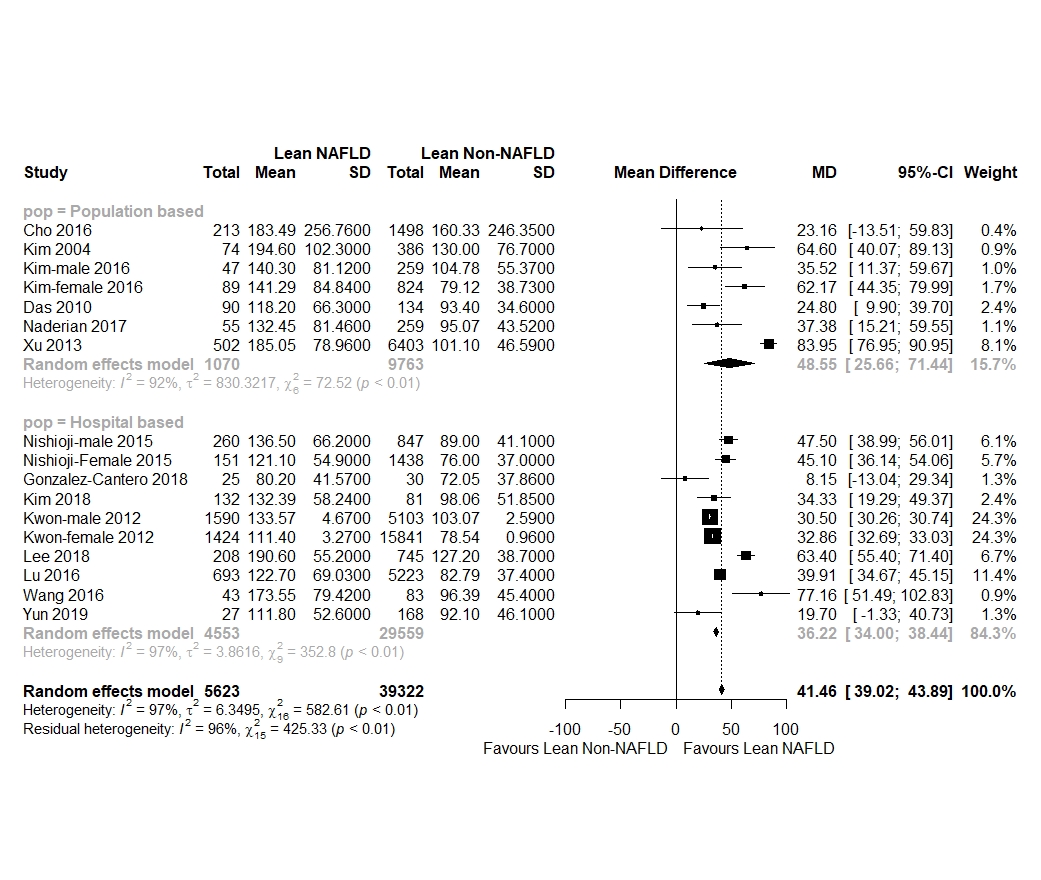


**Supplementary Figure 13B. Forrest plot for TG - Subgroup with respect to population.**


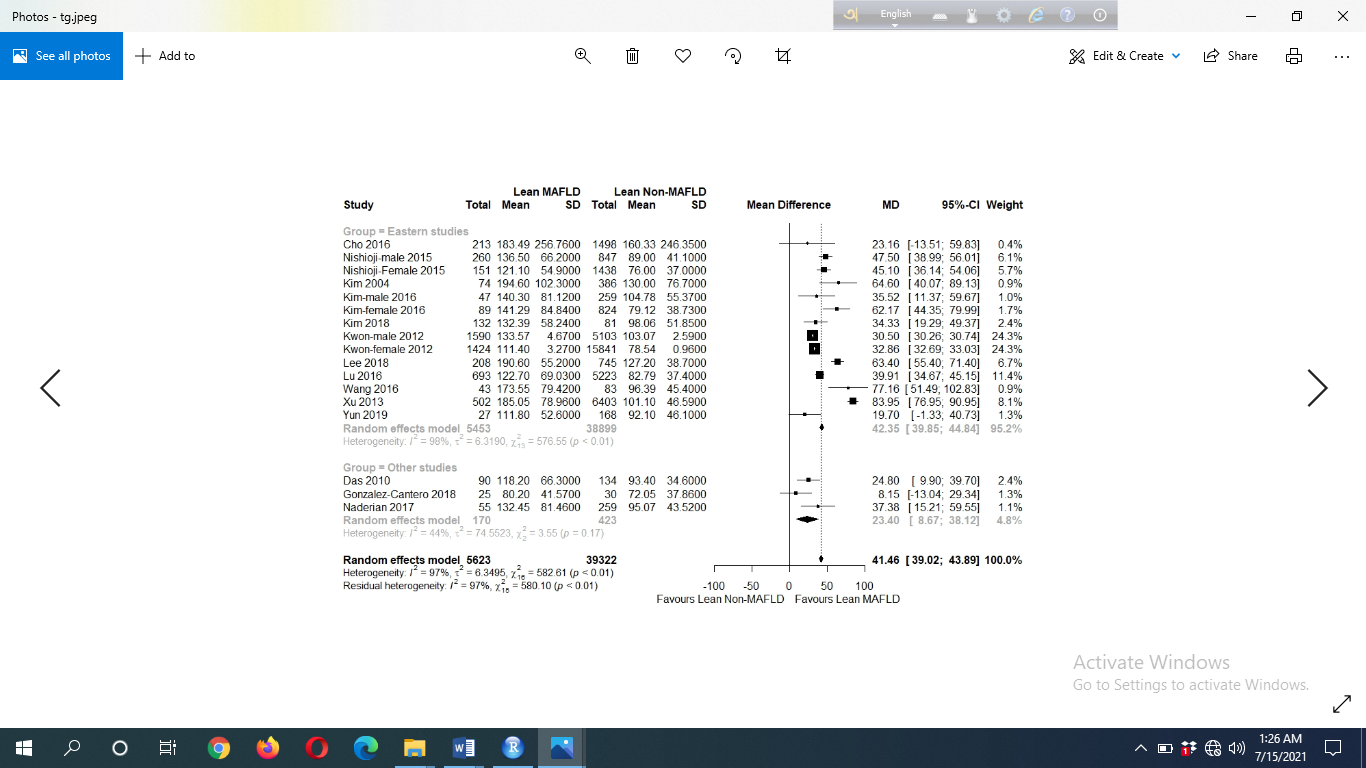


**Supplementary Figure 13C. Forrest plot for TG - Subgroup with respect to Eastern and other studies.**


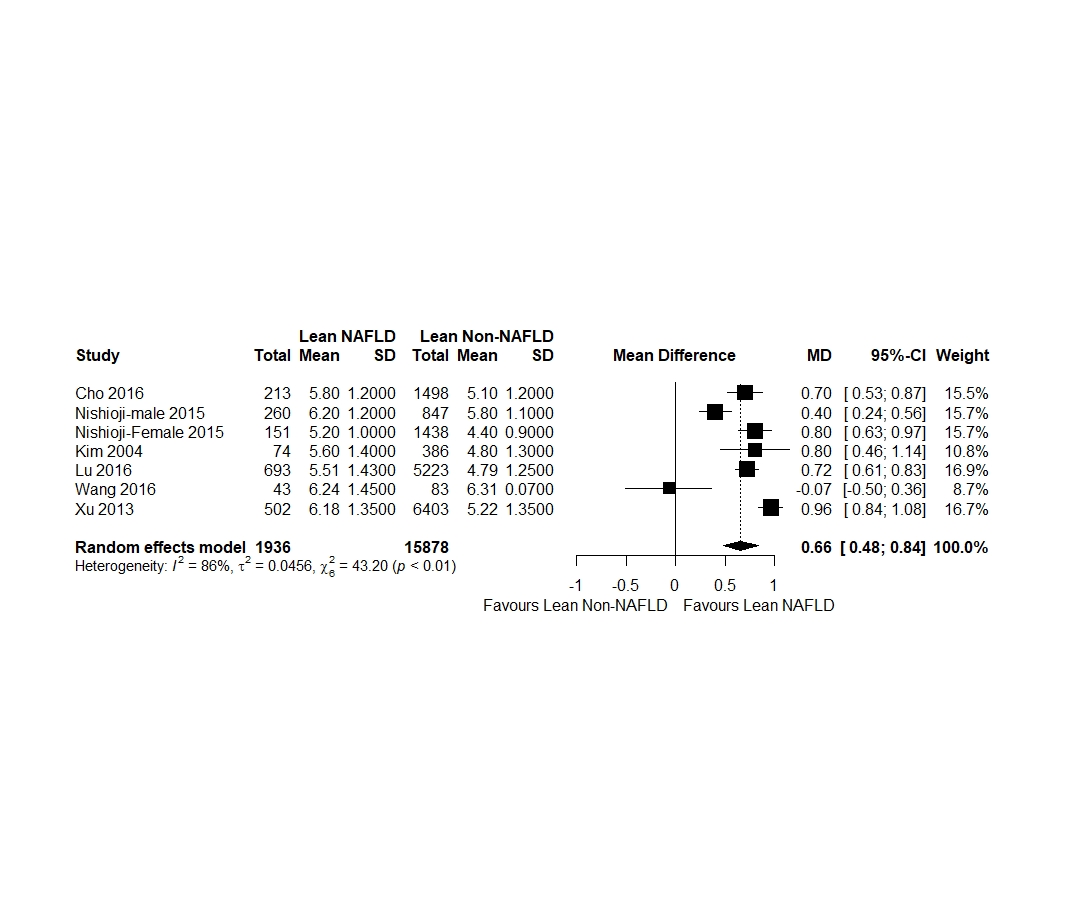


**Figure 14. Forest plot for uric acid.**


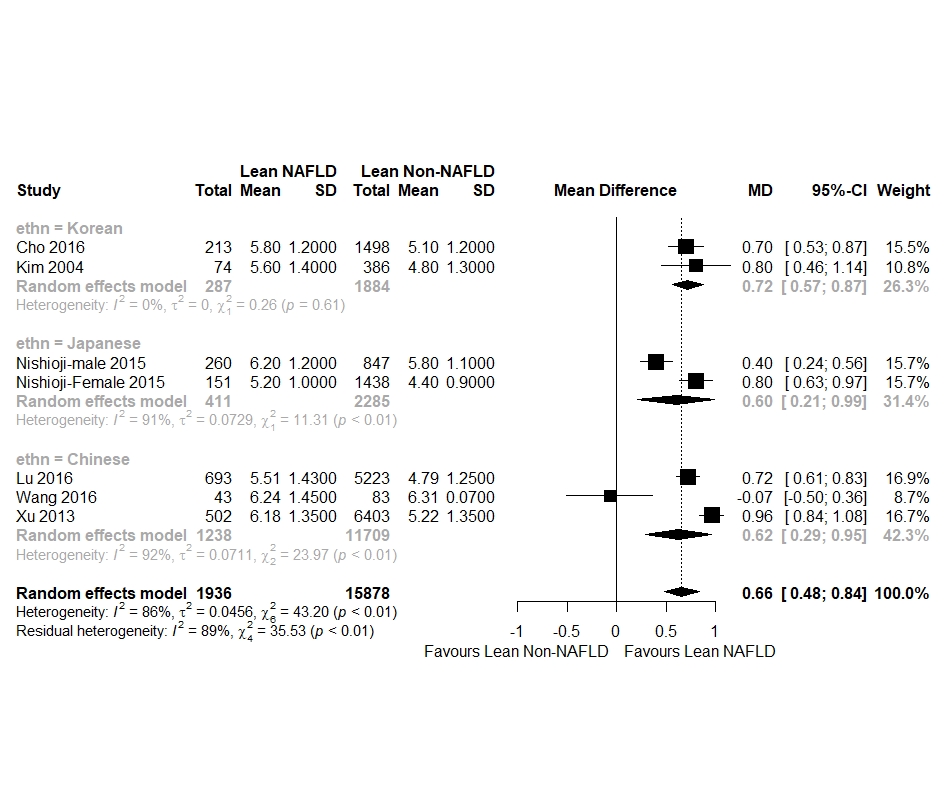


**Supplementary Figure 14A. Forrest plot for uric acid- Subgroup with respect to ethnicity.**


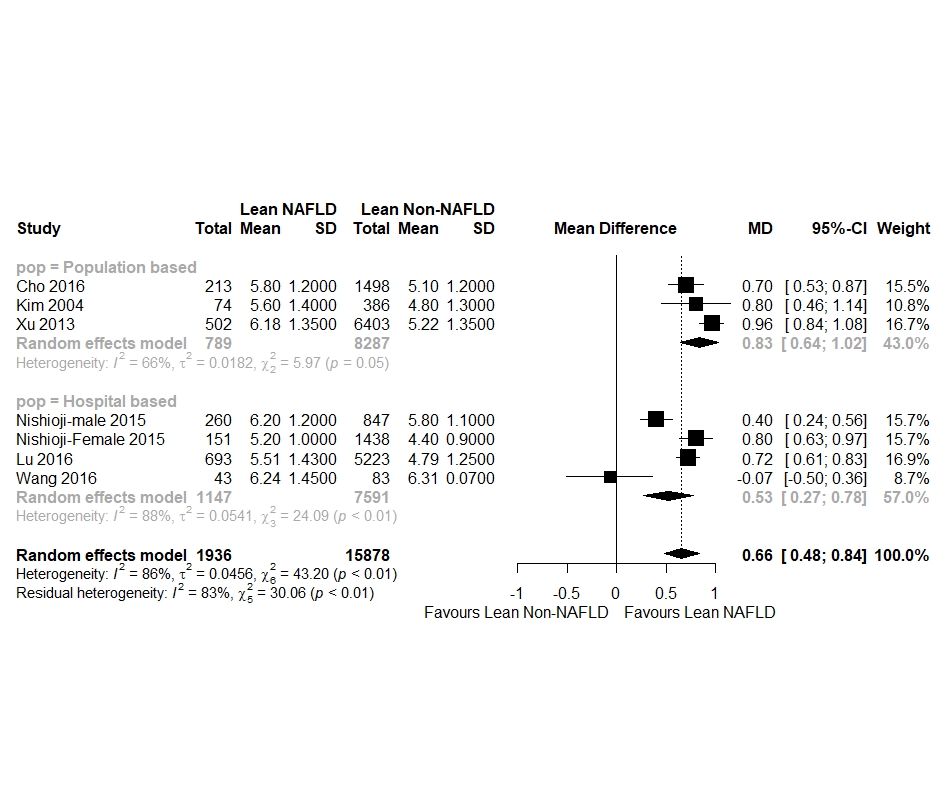


**Supplementary Figure 14B. Forrest plot for uric acid- Subgroup with respect to population.**


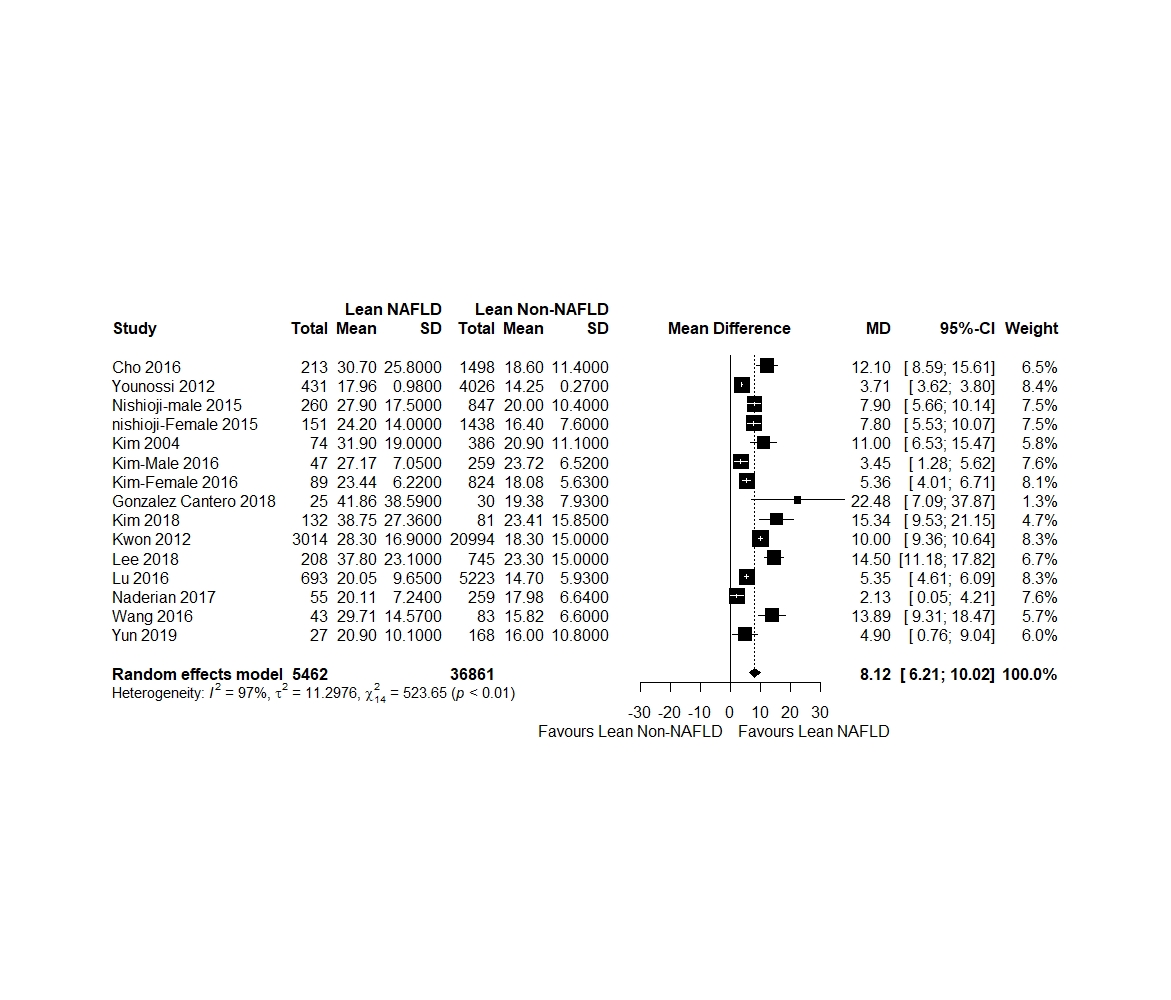


**Supplementary Figure 15A. Forest plot for ALT.**


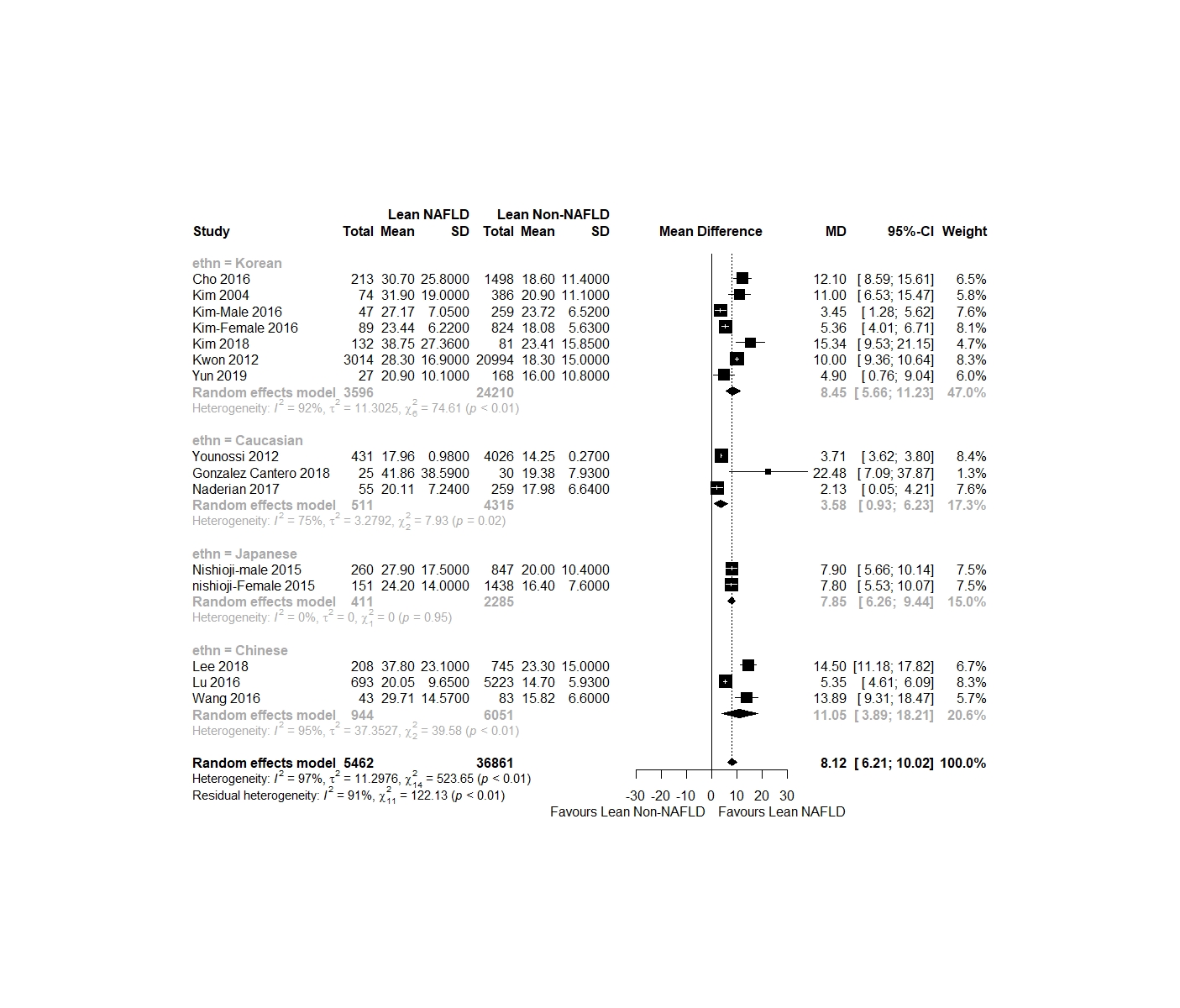


**Supplementary figure 15B. Forrest plot for ALT - Subgroup with respect to ethnicity.**


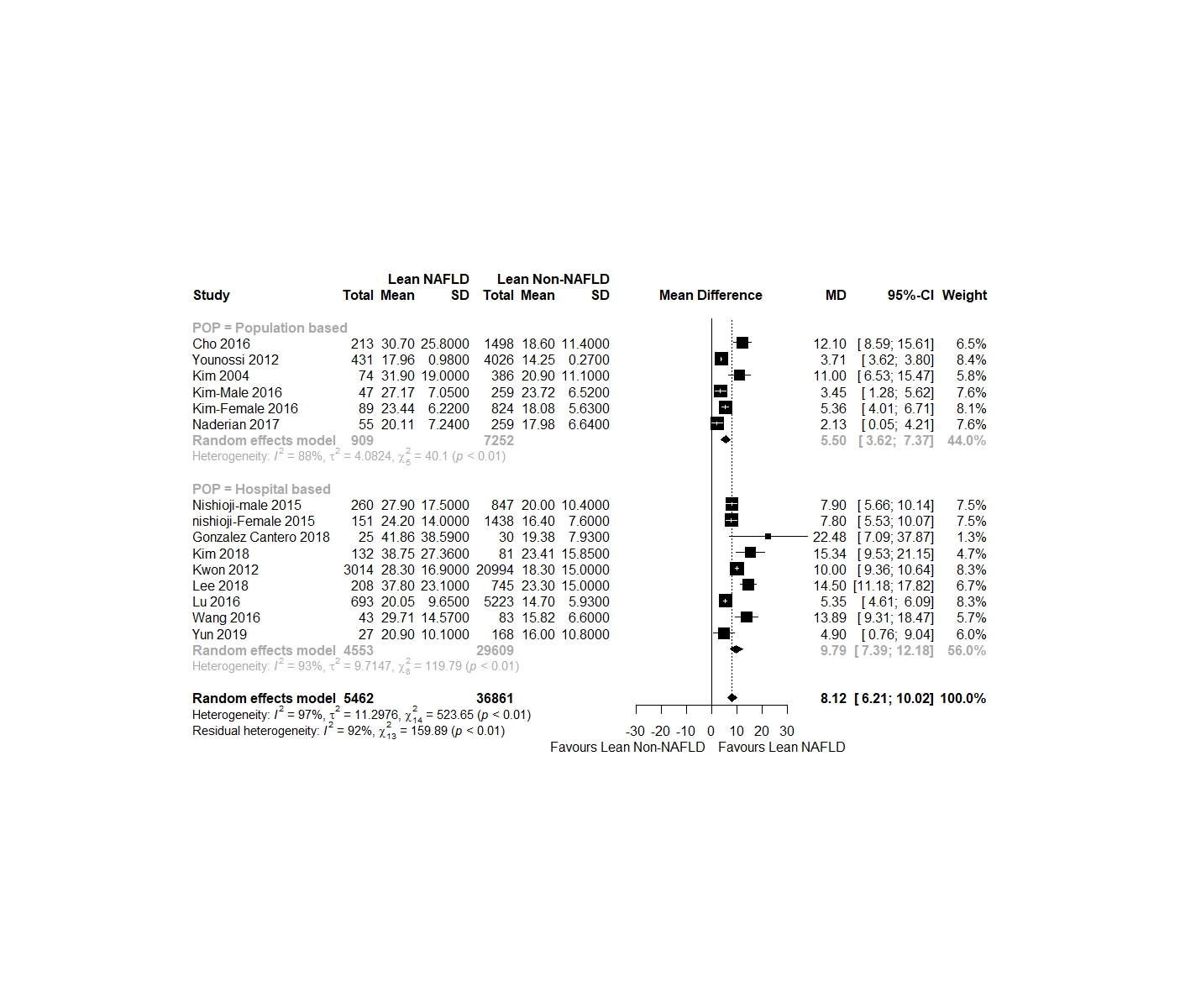
 **Supplementary figure 15C. Forrest plot for ALT- Subgroup with respect to population.**


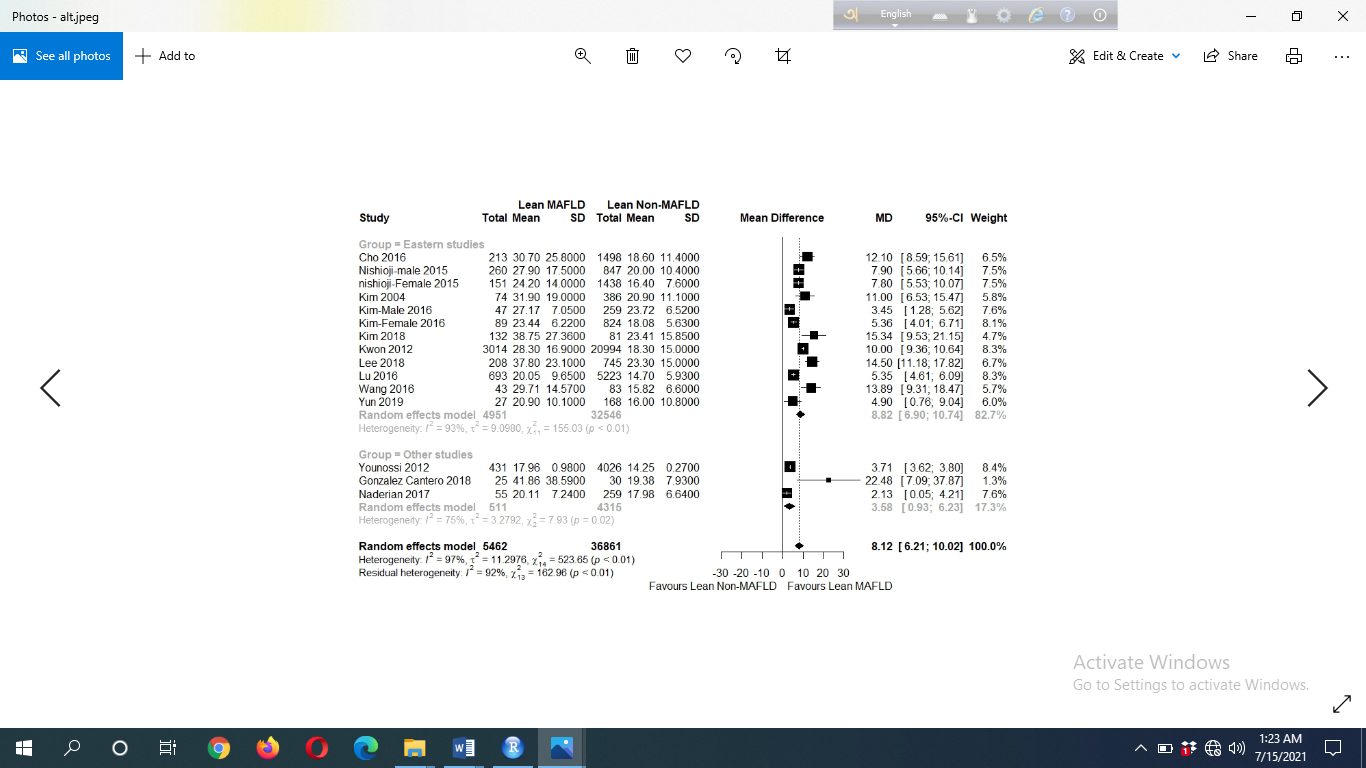


**Supplementary figure 15D. Forrest plot for ALT- Subgroup with respect to Eastern and other studies.**


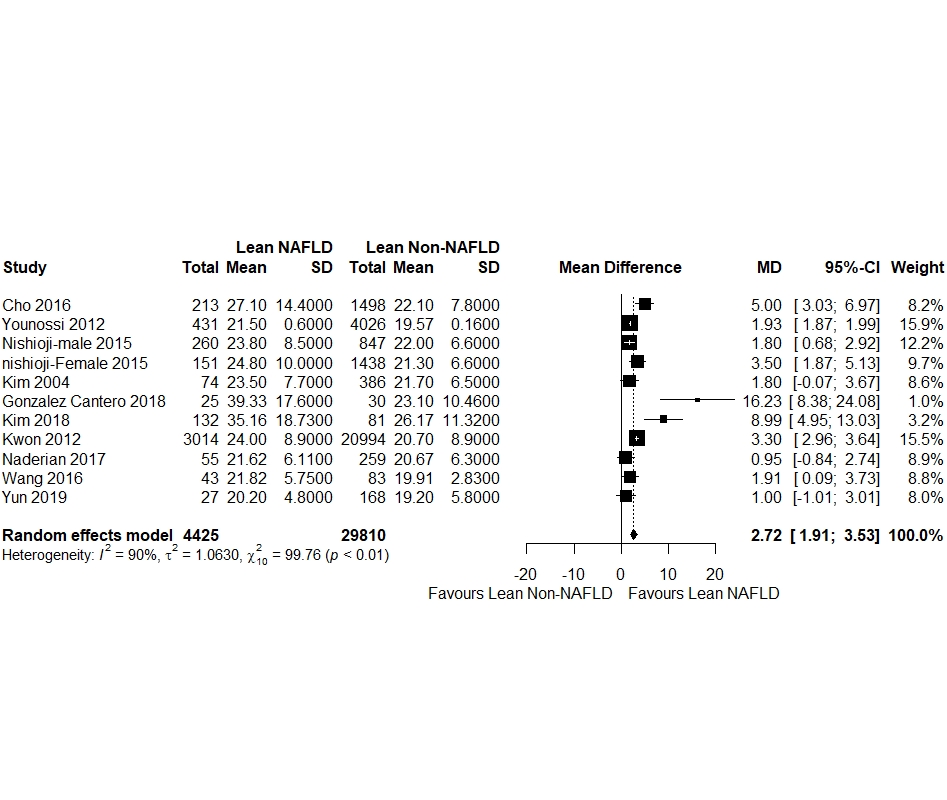


**Supplementary Figure 16A. Forest plot for AST.**


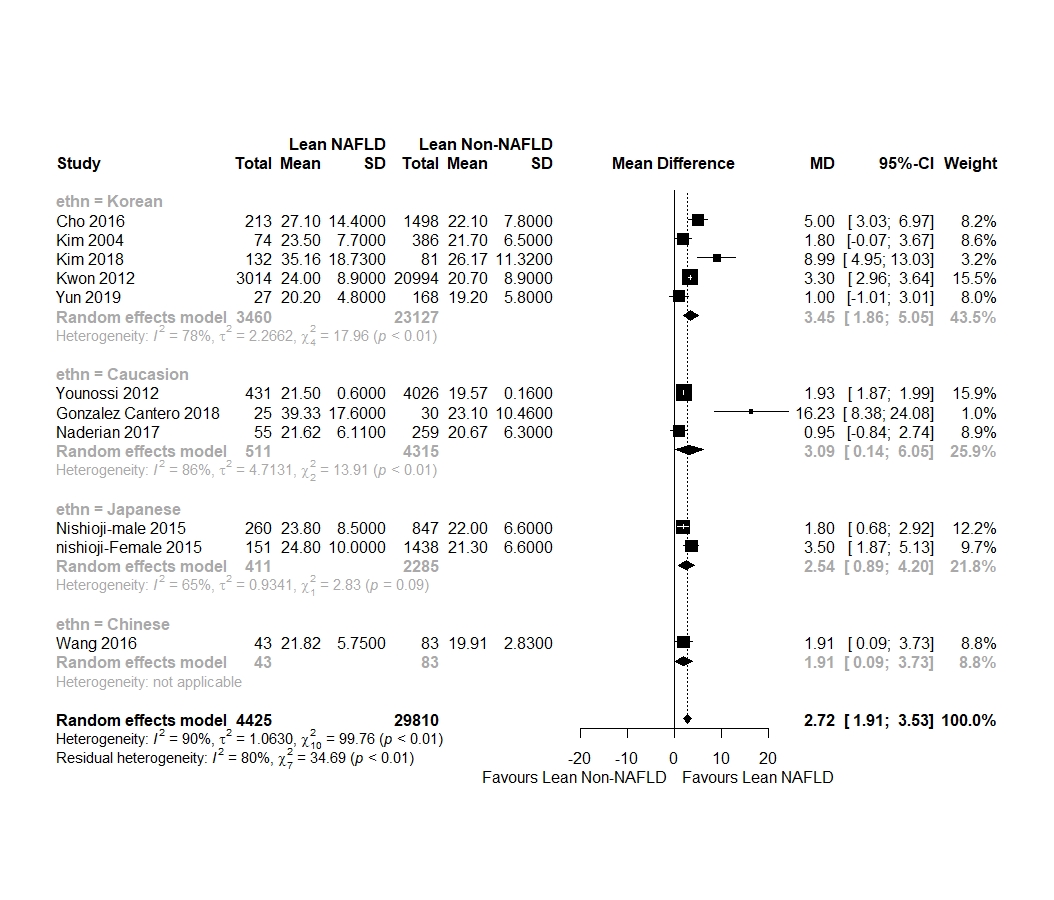


**Supplementary figure 16B. Forrest plot for AST- Subgroup with respect to ethnicity**.


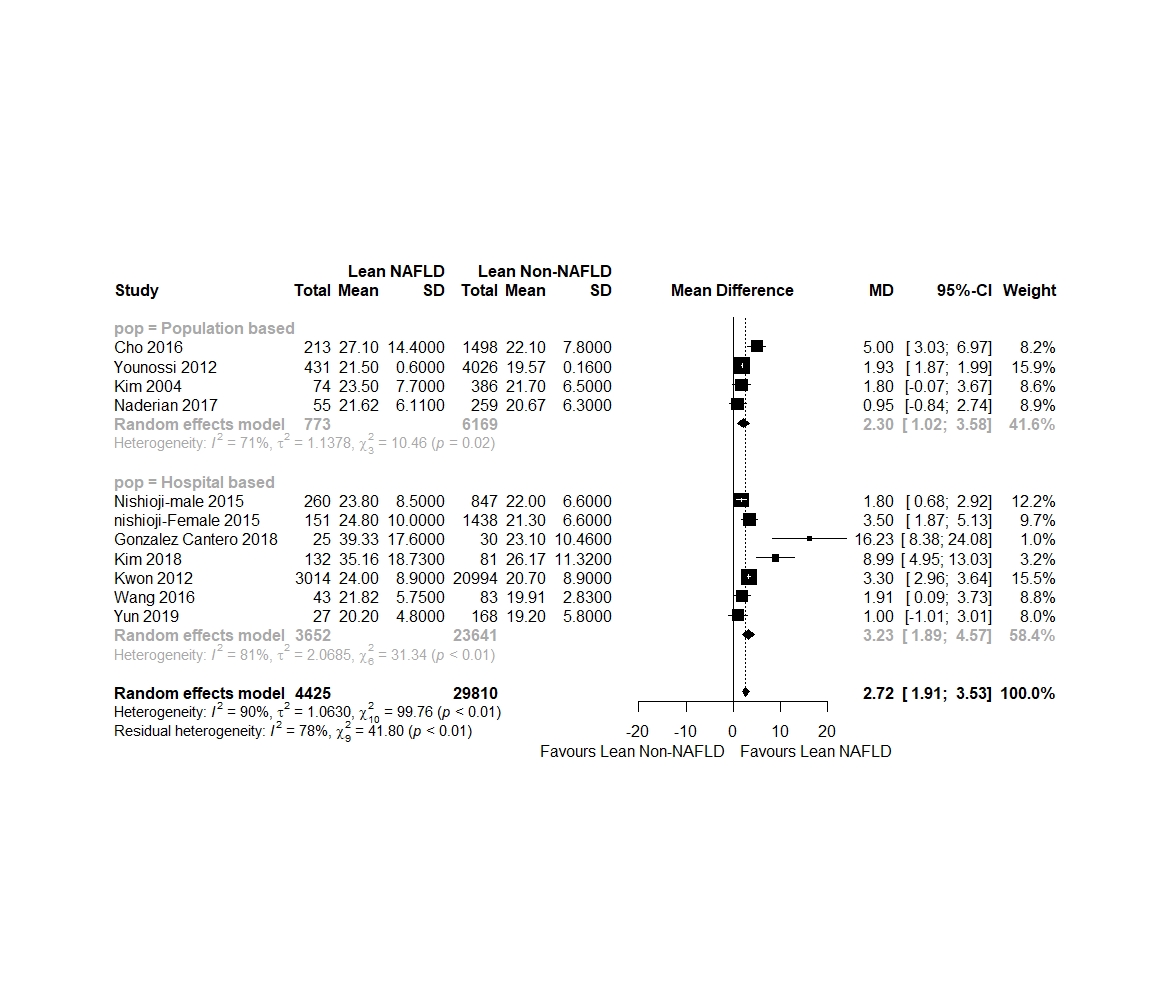


**Supplementary figure 16C. Forrest plot for AST- Subgroup with respect to population.**


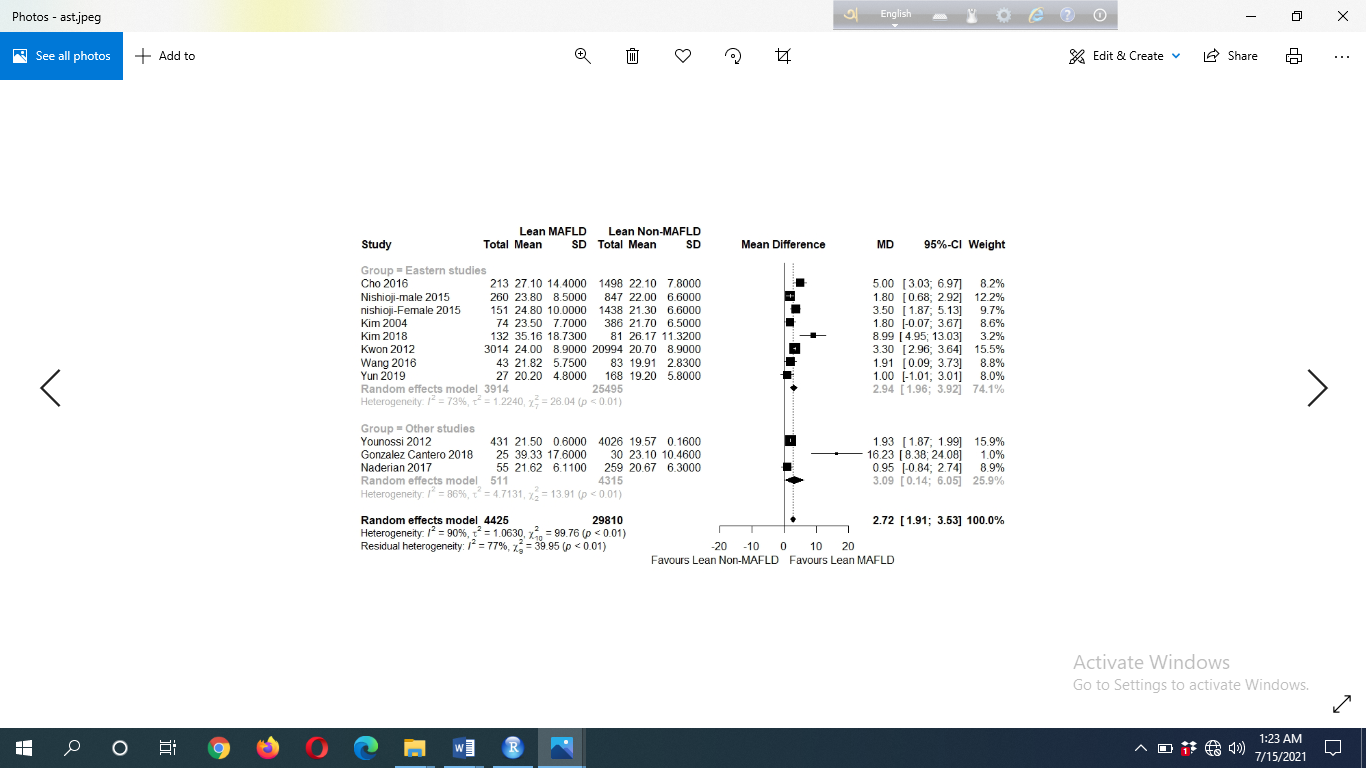


**Supplementary figure 16D. Forrest plot for AST- Subgroup with respect to Eastern and other studies.**


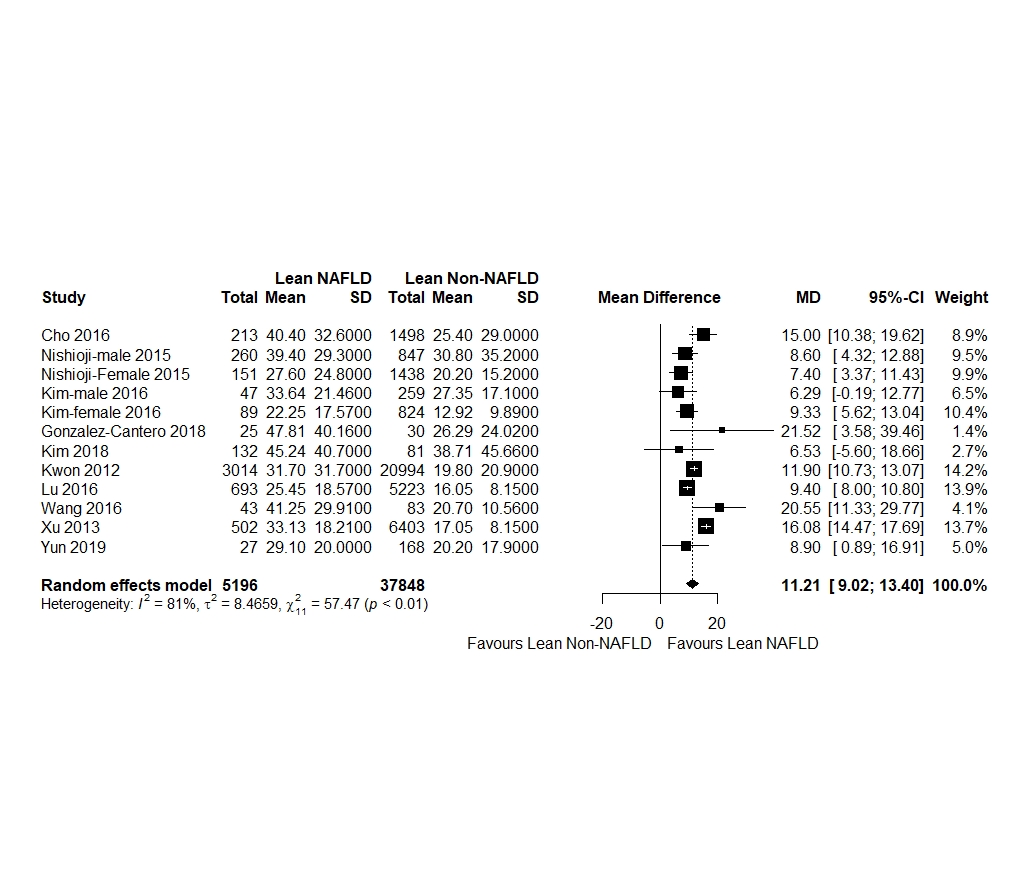


**Supplementary Figure 17A. Forest plot for GGT.**


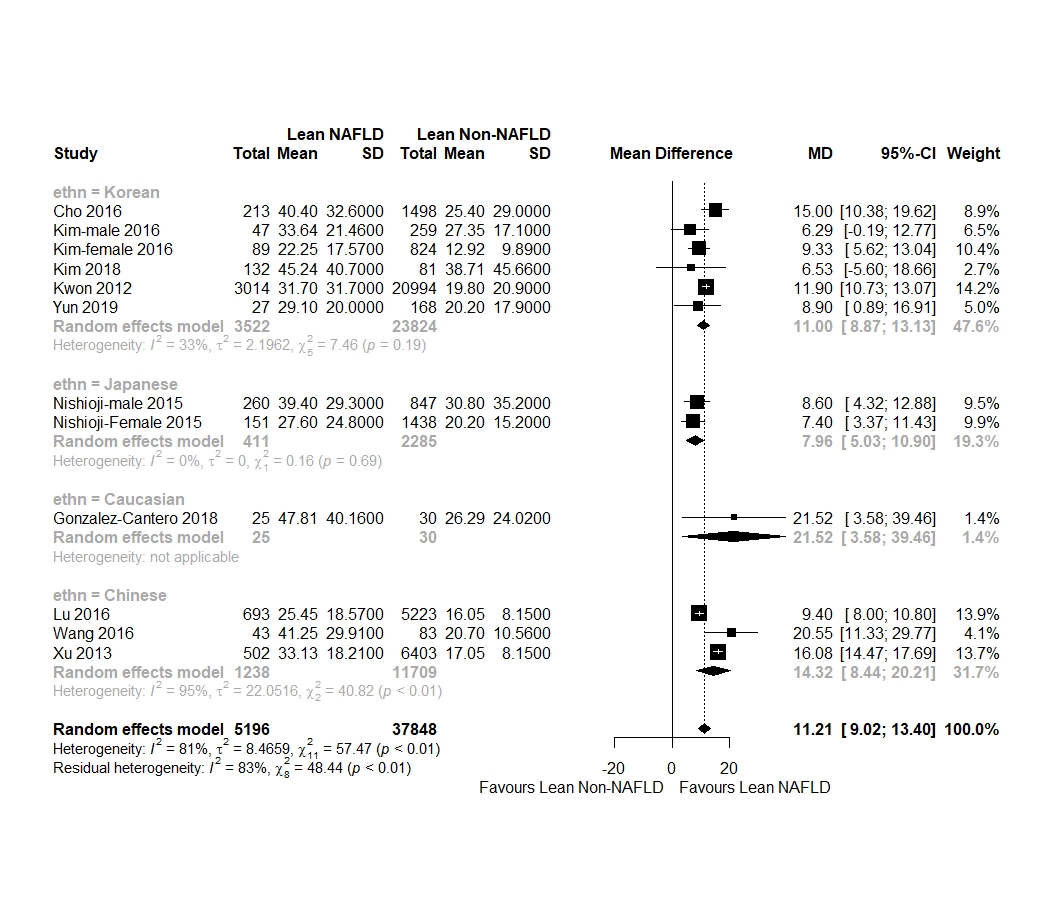


**Supplementary figure 17B. Forrest plot for GGT- Subgroup with respect to ethnicity.**


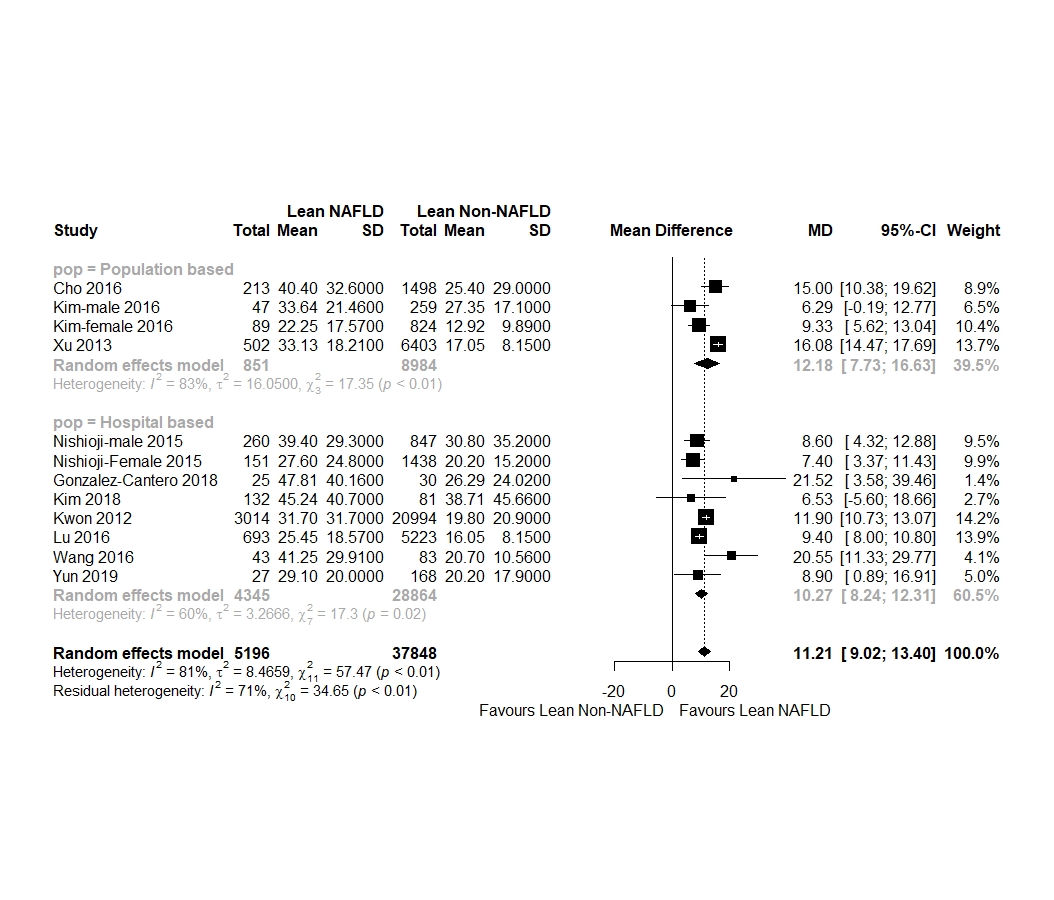


**Supplementary figure 17C. Forrest plot for GGT- Subgroup with respect to population**.


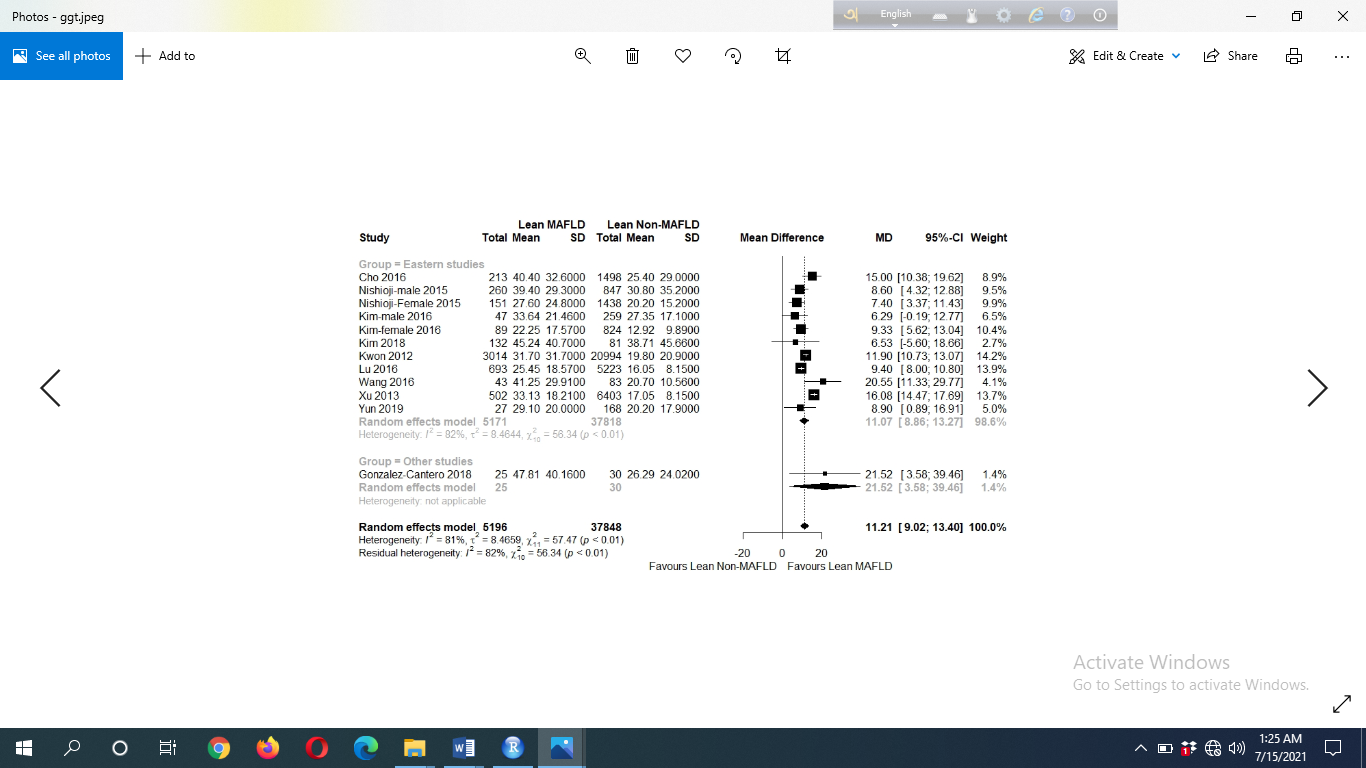


**Supplementary figure 17D. Forrest plot for GGT- Subgroup with respect to Eastern and Other studies**


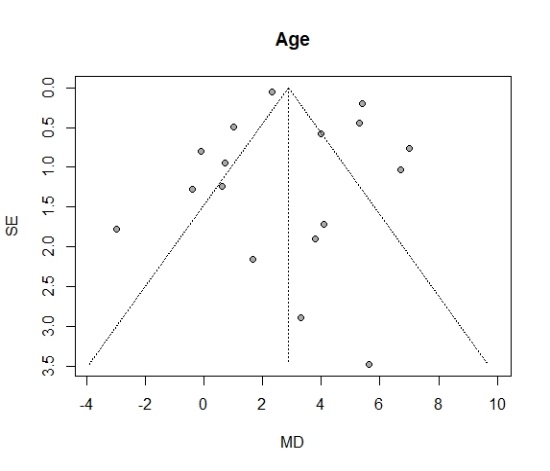

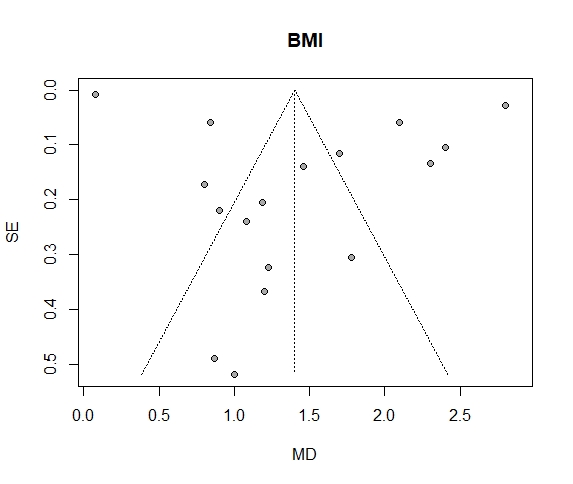

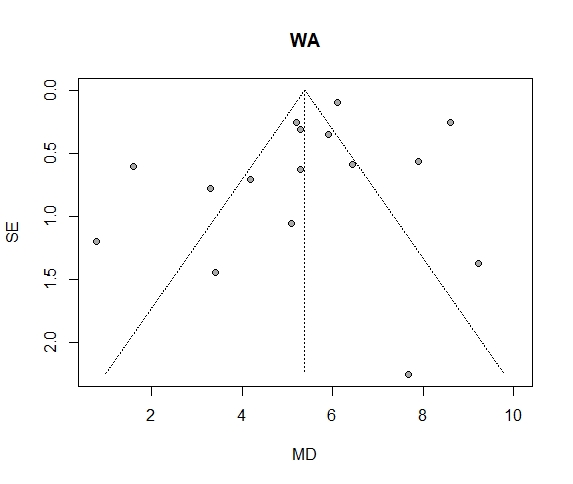

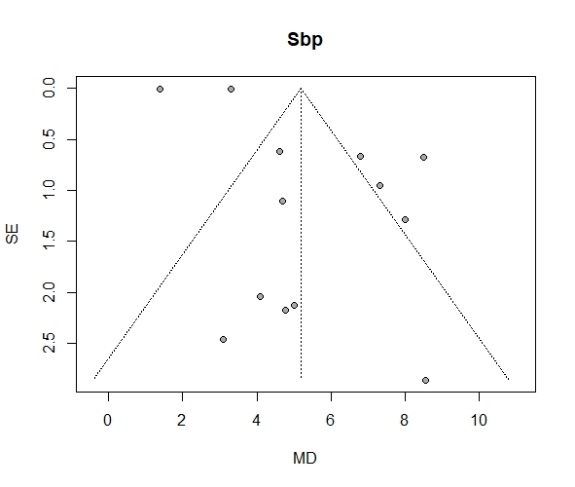


WC

SBP

Age

BMI


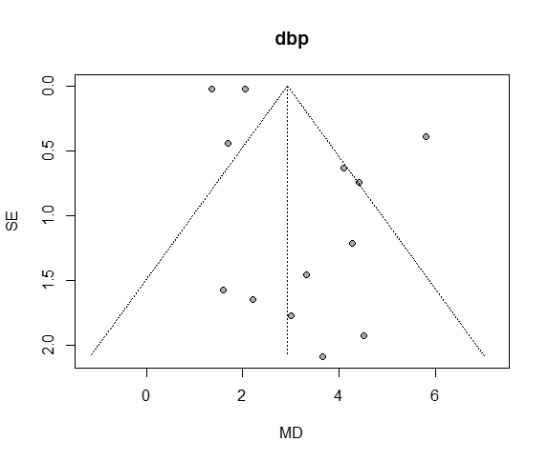

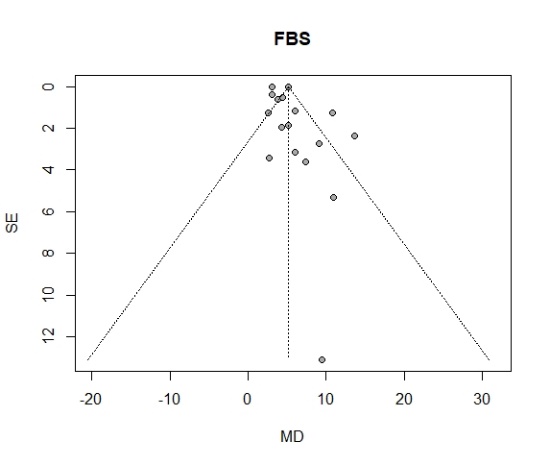

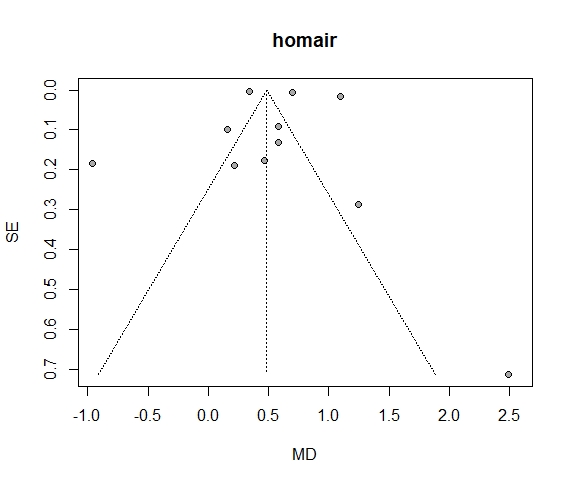


DBP

FBS

HOMA-IR

**Supplementary Figure 18. Funnel plots for different variables**


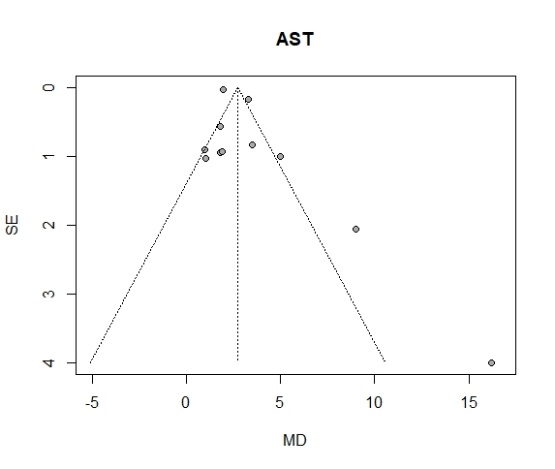

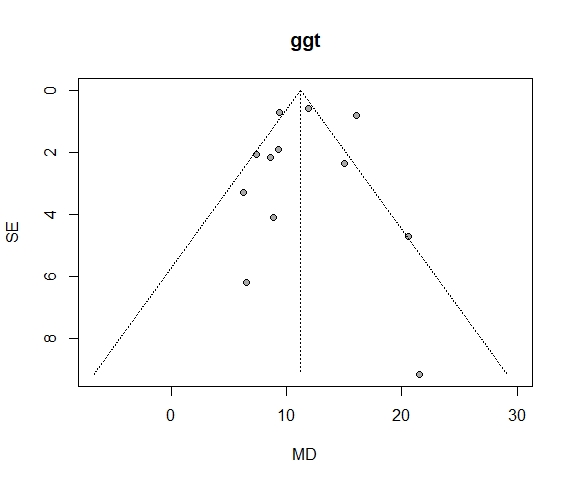

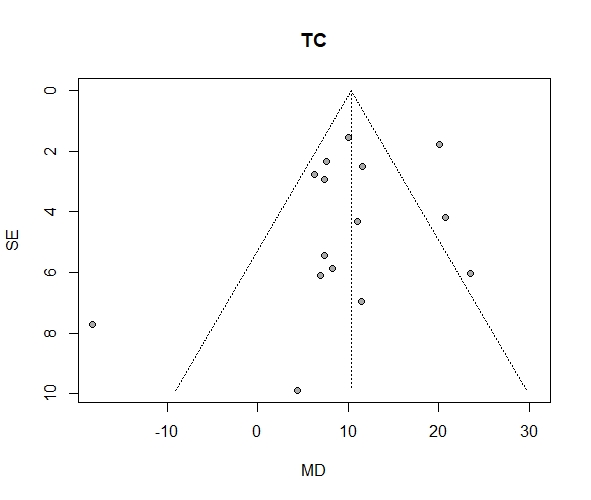


AST

GGT

TC


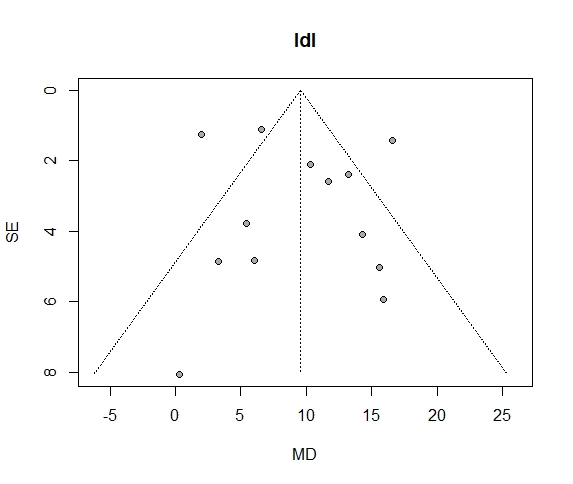

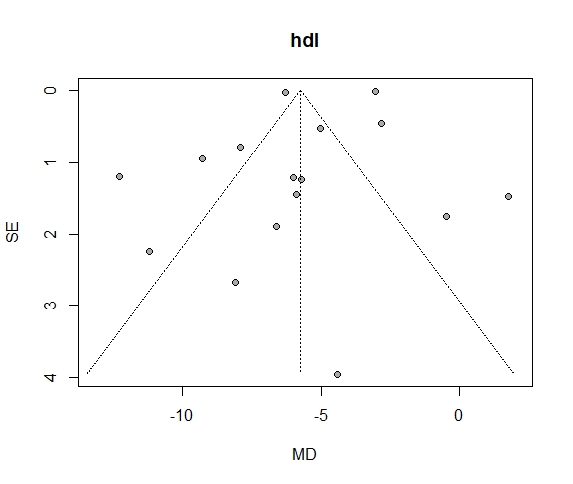

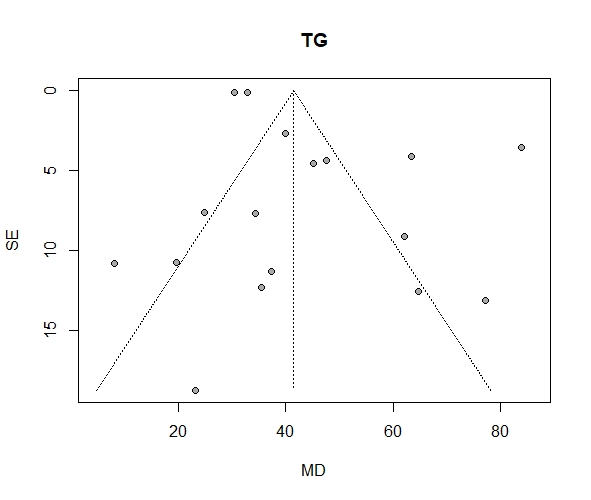


LDL

HDL

TG


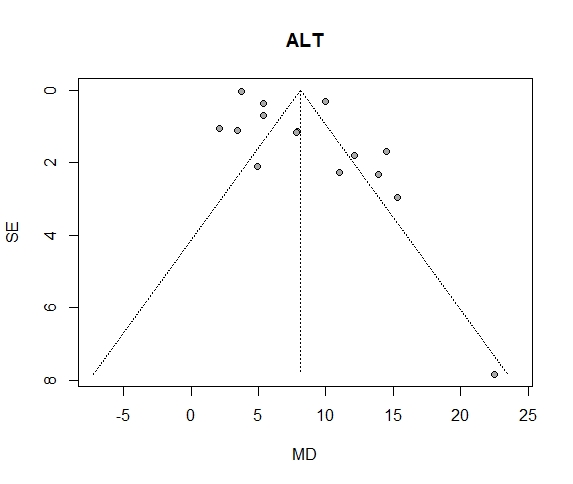


ALT

**Supplementary Figure 18 (continued). Funnel plots different variables**
